# Synthesis and Characterization of New Triazole-Bispidinone Scaffolds and Their Metal Complexes for Catalytic Applications

**DOI:** 10.3390/molecules28176351

**Published:** 2023-08-30

**Authors:** Arianna Rossetti, Alessandro Sacchetti, Fiorella Meneghetti, Greta Colombo Dugoni, Matteo Mori, Carlo Castellano

**Affiliations:** 1 Department of Chemistry, Materials and Chemical Engineering “G. Natta”, Politecnico di Milano, Via Mancinelli 7, 20131 Milano, Italy; alessandro.sacchetti@polimi.it (A.S.); greta.colombodugoni@polimi.it (G.C.D.); 2 INSTM—Local Unit c/o Politecnico di Milano, Via Mancinelli 7, 20131 Milano, Italy; 3 Department of Pharmaceutical Sciences, University of Milan, Via L. Mangiagalli 25, 20133 Milano, Italy; matteo.mori@unimi.it; 4 Department of Chemistry, University of Milan, Via C. Golgi 19, 20133 Milano, Italy; carlo.castellano@unimi.it

**Keywords:** bispidine, click chemistry, metal coordination, NMR titration, crystal structure, triazoles, catalysis, Henry reaction

## Abstract

Bispidines are a family of ligands that plays a pivotal role in various areas of coordination chemistry, with applications in medicinal chemistry, molecular catalysis, coordination polymers synthesis, and molecular magnetism. In the present work, triazole moieties were introduced using the CuAAC click-reaction, with the aim of expanding the number of coordination sites on the bispidine core. The 1,2,3-triazole rings were thus synthesized on propargyl-derived bispidines after reaction with different alkyl azides. The new class of triazole-bispidines was characterized, and their chelation capabilities were evaluated with different metals through NMR titration, ESI-MS spectrometry, and single-crystal X-ray diffraction (SC-XRD). Finally, the suitability of these molecules as metal ligands for the catalytic Henry reaction was demonstrated with copper and zinc.

## 1. Introduction

Bispidines are a family of ligands that have been extensively applied in the last two decades in various areas of coordination chemistry due to their interesting properties. They are based on the 3,7-diazabicyclo[3.3.1]nonane scaffold and are found in the structure of some natural products, such as sparteine and cytisine [1]. The diazabicyclo[3.3.1]nonane core is a bi-dentate ligand, relying on the coordination activity of the two nitrogen atoms. It can be conveniently prepared by a classical multi-Mannich reaction approach, allowing for the obtainment of many differently decorated structures capable of efficiently coordinating metals in a highly preorganized way. By the introduction of specific substituents, many tetra-, penta-, hexa-, hepta-, and octadentate ligands have been reported in the literature [1,2,3,4,5,6,7,8]. Most of their applications are related to the rigidity of the bispidine backbone, which confers unique structural and electronic properties to the ligand, namely a relatively large cavity allowing for efficient metal ion encapsulation. In this respect, bispidines are expected to lead to very stable complexes with a pronounced selectivity for the size (and electronic requirements) of the metal ion. Hence, these ligands have been successfully applied in many fields, including medicinal chemistry [5,9], molecular catalysis [6,10], coordination polymers synthesis [7,11], and molecular magnetism [12].

To expand the number of coordination sites on the bispidine core, a classical approach is to introduce additional sp^2^ nitrogen atoms, usually by adding one or more pyridine rings in different positions of the diazabicyclo scaffold [2,13]. For the same purpose, pyrazole rings, macrocyclic amides, and oxygen-containing substituents such as alcohols or carboxylate groups have also been proposed [2,13]. Recently, the introduction of the picoline residue by Comba allowed coordination numbers higher than eight to be achieved [5]. Additionally, the use of different coordinating atoms can be useful in tuning the interaction with the cation towards an increased selectivity of the ligands for the metals.

In our ongoing studies on the properties and applications of bispidines [2,6,11], we decided to introduce the 1,2,3-triazole ring on the bispidine core to expand the coordination capabilities of this ligand. The 1,2,3-triazole has gained great interest in the last decades due to the success of the Cu alkyne-azide cycloaddition, the so-called CuAAC click-reaction [14]. These functional-group-tolerant and easily synthesizable heterocycles have gradually become important building blocks in organic synthesis, not only as linkers between chemical or biological components, but also as efficient coordinating agents for metal ions [15,16,17]. The importance of 1,2,3-triazole-based ligands in catalyzed chemical transformations has recently been reported in the literature [18]. Moreover, the presence of three consecutive sp^2^ nitrogen atoms has been shown to confer to the 1,2,3-triazole ring particular electron-donating capabilities [19].

In the present work, we synthesized the 1,2,3-triazole ring on a propargyl-derived bispidine core through a copper-catalyzed azide-alkyne Huisgen 1,3-dipolar cycloaddition (CuAAC, click reaction) with different alkyl azides. Hence, a small library of symmetrical tetradentate bispidines was prepared with an efficient reaction protocol involving the use of the microwaves, in order to reduce the reaction time while improving the yields. The chelating properties of the triazole-based bispidines thus synthesized were deeply investigated by means of SC-XRD and NMR. Finally, the efficacy of these molecules as metal ligands for the catalytic Henry reaction was demonstrated with two different metals: copper and zinc.

## 2. Results and Discussion

### 2.1. Synthesis of the Bispidine-Triazole Ligands

**Scheme 1:**
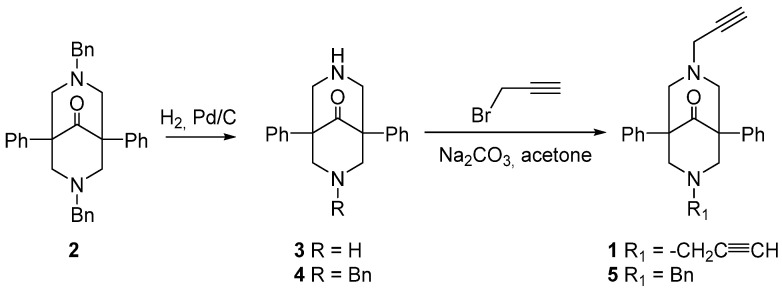
Synthesis of propargyl precursors **1** and **5**.

The key intermediate for the preparation of the new bispidine ligands was the bis-propargyl derivative **1** (Scheme 1). In a first attempt, we tried to obtain this compound by reacting 1,3-diphenylacetone with two equivalents of propargylamine and an excess of formaldehyde by a classical Mannich reaction. However, the very low yields prompted us to try a different approach, starting from the preparation of the dibenzyl analogue **2**, which was easily synthesized in high yields according to a procedure inspired by Black and co-workers [3]. The removal of the benzyl group through a palladium-catalyzed hydrogenation afforded the bispidine **3**, together with a lower amount of mono-benzylated derivative **4**, which were separated through column chromatography. The further reaction of **3** with propargyl bromide and sodium carbonate as an inorganic base afforded the desired intermediate **1**. Product **4**, obtained by the partial hydrogenation of **2**, was also employed in basic conditions with propargyl bromide to prepare the unsymmetrical bispidine **5**.

**Scheme 2:**
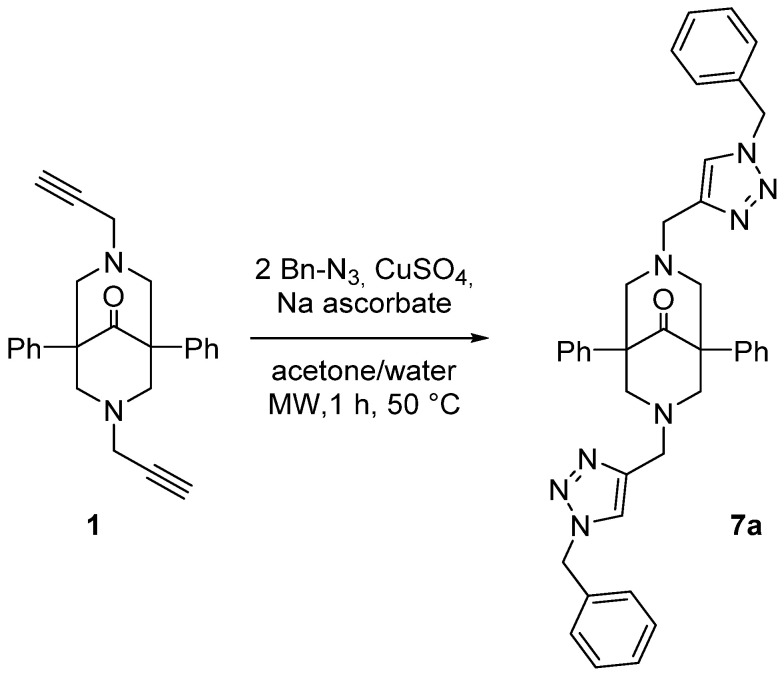
Click reaction of **1** with azide **6a**.

Once intermediates **1** and **5** were achieved, we studied the CuAAC 1,3-dipolar cycloaddition on our scaffolds. As a reference reaction for establishing the best set-up conditions, we employed **1** and the benzyl azide **6a**. Firstly, the CuAAC reaction was performed with 2 eq. of the azide in a 1:1 acetone/water mixture, using CuSO_4_ and sodium ascorbate at 50 °C. After 24 h, the reaction of **1** with the benzyl azide **6a** afforded the desired product with a poor yield (23%). When the reaction was performed under microwave (MW) irradiation at 50 °C, the product was obtained after 1 h in 95% yield (Scheme 2).

The removal of the copper catalyst from the crude was quite troublesome, because, as expected, the newly formed bispidine ligand **7a** bound to the Cu cation very efficiently; therefore, a simple aqueous wash as work-up was not sufficient. Several attempts were made to remove the metal by ligand exchange, washing the organic layer with aqueous solutions of various amines, such as ammonia, trimethylamine, and ethylenediamine at different concentrations. In every instance, the desired bispidines were extracted in low yields, probably due to the degradation of the product in the basic conditions of the work-up. Finally, we managed to cleanly isolate the product by washing the organic phase with a 1 M aqueous solution of ethylenediaminetetraacetic acid (EDTA). With this protocol, the desired compound was isolated in high yields and without the need for further purifications.

**Scheme 3:**
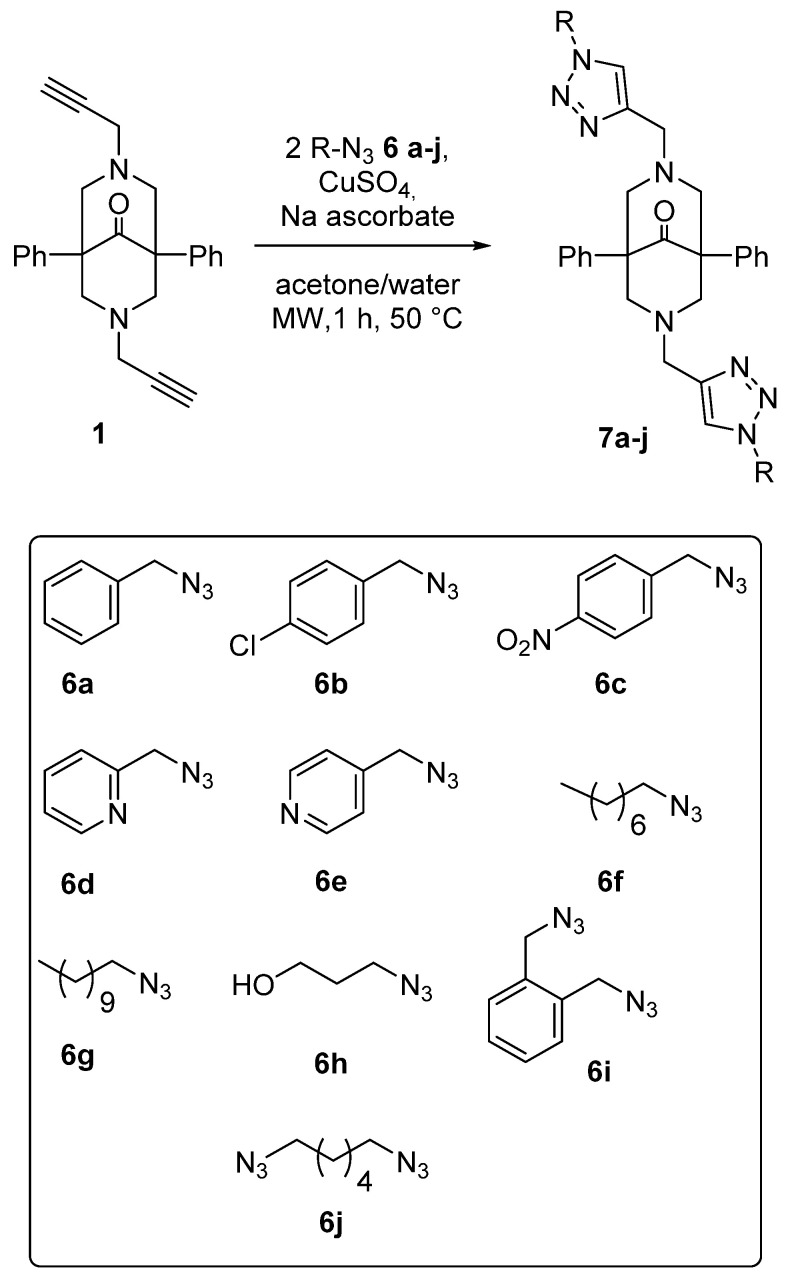
Click reaction of **1** with azides **6a**–**j**.

Once established the optimum for the reaction set-up, a library of aryl- and alkyl-azides was selected (**6a**–**j**, Scheme 3) to be reacted with **1** or **5** to evaluate the potential chelation capabilities of the resulting derivatives.

All the azido-derivatives **6a**–**j** were efficiently prepared by reaction of sodium azide with the corresponding alkyl or benzyl bromide in *N*,*N*-dimethylformamide (DMF) under MW irradiation at 100 °C for 15 min. The synthesized azides were selected to tune both the coordination properties and the solubility of the bispidine derivatives, thus allowing the design of efficient ligands for their final applications. For instance, two pyridine-based azides were prepared: the 2-(azidomethyl)pyridine **6d** and 4-(azidomethyl)pyridine **6e**; the presence of these aromatic rings was designed in an attempt to further expand the coordination capabilities of the final bispidine ligands. The aliphatic 1-azidooctane **6f** and 1-azidoundecane **6g** were considered to increase the lipophilicity of the final products and promote their solubility in poorly polar organic solvents. With the same purpose, in addition to the benzyl azide **6a**, the aromatic 1-(azidomethyl)-4-chlorobenzene **6b**, and 1-(azidomethyl)-4-nitrobenzene **6c** were also prepared. On the contrary, 3-azidopropan-1-ol **6h** was prepared to improve the solubility of the target ligand in protic polar solvents, such as alcohols and water. Finally, the bis-azido derivatives 1,2-bis(azidomethyl)benzene **6i** and 1,3-diazidohexane **6j** were investigated to evaluate the feasibility of obtaining macrocyclic ligands.

Following the optimized procedure for the CuAAC reaction, a family of triazole-bispidine derivatives (**7a**–**j**) was thus prepared by coupling **1** with the corresponding azides (**6a**–**j**).

As shown in Table 1, most of the bis-triazole bispidines were obtained in good/excellent yields with both aromatic and aliphatic compounds (**7a**–**h**). On the contrary, the use of diazide linkers to produce macrocycles was unsuccessful, and products 7**i**–**j** were detected in traces.

**Scheme 4:**
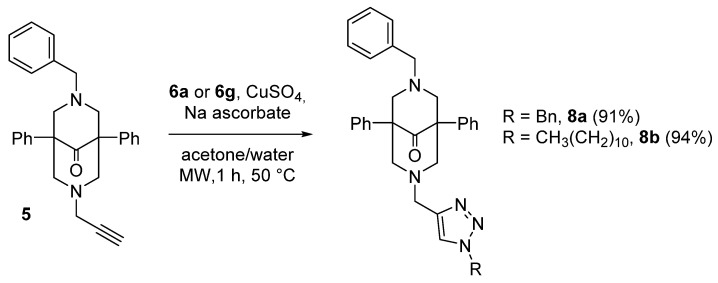
Click reaction of **5** with azides **6a** or **6g**.

The unsymmetrical propargyl-bispidine **5** was employed as a substrate for the CuAAC cycloaddition in the same reaction conditions (Scheme 4). Azides **6a** and **6g** were used as reference compounds, and the subsequent products **8a** and **8b** were isolated in high yields.

### 2.2. NMR Studies on the Coordination Chemistry

An extensive investigation of the coordination chemistry was conducted by performing ^1^H-NMR titrations of the synthesized bispidines with various metals (such as Zn, La, and Pd). The objective was to analyze how the chelation number changed based on the different substituents incorporated into the frameworks. The chelation number, which represents the atoms directly coordinating to the central metal ion in a chelate complex, is evidently influenced by the geometry and electron configuration of the metal ion, as well as the coordinating sites of the ligand.

**Figure 1:**
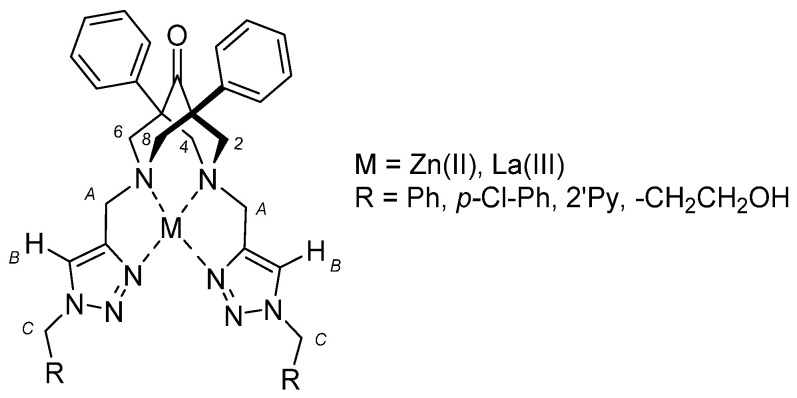
Coordination geometry of ligands **7a**,**b**,**d**,**h** and their metal complexes.

As a start, we studied the formation of the complexes of compounds **7b** and **7d** with Zn(II), and of **7a**,**d**,**h** with La(III) (Figure 1). These cations were selected as representative of bi- and trivalent metals.

**Figure 2:**
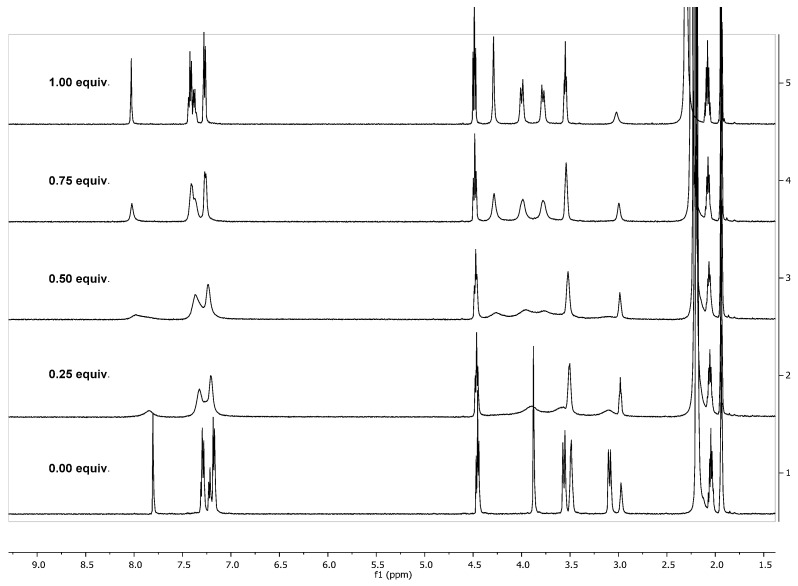
^1^H-NMR titration of compound **7h** with La(NO_3_)_3_.

To investigate the coordination chemistry, ^1^H-NMR titration studies were performed in CD_3_CN as the deuterated solvent. Spectra were recorded after the addition of precise aliquots of the metal into the ligand solution. Upon addition of the salt, a progressive minor broadening of some signals was observed, with the concomitant appearance of new peaks (Figure 2). In all the tested samples, the addition of an equivalent of metal resulted in the formation of a single new species, which was attributed to the ligand-metal complex in a 1:1 ratio; further addition of the metal did not cause any significant change in the spectrum. This behavior is typical of very tight complexes (i.e., showing a very low dissociation constant) in a slow exchange regime between the complex and the free-ligand species.

Once established the stoichiometry of the complexes via NMR titration, the products were also prepared by stirring a solution of the ligand in acetonitrile with a stoichiometric amount of Zn(NO_3_)_2_ or La(NO_3_)_3_ for 24 h. The resulting precipitate was filtered and analyzed by ^1^H-NMR in CD_3_CN. In Table 2, the comparison of the chemical shifts of the free ligands and their complexes is reported.

Concerning compound **7b** and its Zn(II)-complex **7b·Zn(II)**, the most evident shifts were detected for the methylene hydrogens of the bispidine core (H_2,4,6,8_), with values of ∆δ = 0.44 ppm and ∆δ = 0.46 ppm towards low fields for axial and equatorial hydrogens, respectively (Table 2, entry 1). Similarly, the triazole hydrogen H_B_ was deshielded by ∆δ = 0.50 ppm. The benzylic methylene group’s H_C_ also showed a ∆δ = 0.15 ppm shift. These data suggest a coordination with the metal cation involving both the sp^3^ nitrogen of the bispidine core and one of the sp^2^ nitrogen atoms of the 1,2,3-triazole system. The result is a tetracoordinated metal center, formed by the four nitrogen atoms of the bispidine and triazole rings (Figure 1).

Similarly, the metal coordination of ligand **7d** was also investigated with Zn(II). In this case, it was interesting to evaluate whether the pyridine ring could be involved in the coordination of the zinc ion. In Table 2 (entry 2), the comparison of the free ligand **7d** and its 1:1 Zn(II)-complex **7d·Zn(II)** is displayed. The most evident shifts were for the methylene hydrogens of the bispidine core, with values of ∆δ = 0.50 ppm and ∆δ = 0.61 ppm towards low fields for equatorial and axial hydrogens, respectively. Similarly, the triazole hydrogen H_B_ was deshielded by ∆δ = 0.24 ppm. The methylene group’s H_C_ also showed a ∆δ = 0.15 ppm shift. The pyridine hydrogens showed no significant shift; a deshielding of ∆δ = 0.05–0.10 ppm was observed, the same value measured for all the aromatic protons. This result indicated that the pyridine nitrogen was not involved in the coordination of the metal ion, since no important deshielding effects could be observed on the neighboring hydrogens. Again, the coordination resulted in a tetracoordinated metal center, formed by the four nitrogen atoms of the bispidine and triazole rings (Figure 1).

**Figure 3:**
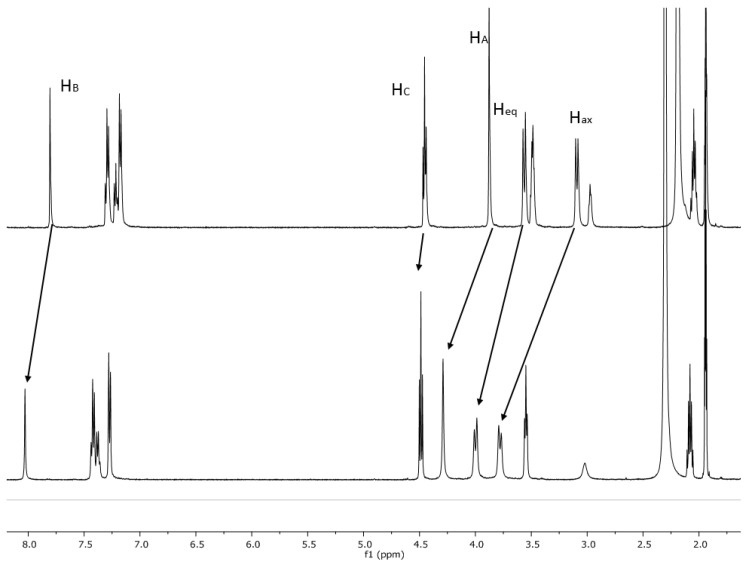
^1^H-NMR spectrum of compound **7h** and its La(III)-complex **7h·La(III)**.

A ^1^H-NMR titration experiment was also performed for compound **7h** in CD_3_CN by adding aliquots of La(NO_3_)_3_ into the ligand solution. After the first additions, a strong broadening of the signals was observed in the whole spectrum, with the exception of the 3-hydroxypropyl portion, thus indicating that no coordination occurred at this part of the molecule. After the addition of 1 equivalent of metal, a single species formed, and the resolution of the spectrum improved (Figure 2 and Figure 3). The pattern of the titration suggests that with less than 1 equivalent of metal, more species are present, indicating that in these conditions, the coordination is not complete, but a rapid exchange regime between complexed and free ligand is established.

To summarize, very similar effects were observed for the complexation of La(III) with ligands **7a**,**d**,**h**. Compared to the Zn(II) complexes, the methylene protons H_A_ showed a higher value of ∆δ, whereas a smaller value was observed for H_C_.

Among the analyzed products, complexes **7d**,**h-La(III)** have an extra coordinating atom, namely the pyridine nitrogen in **7d**, and the hydroxy group of the 3-hydroxypropyl chain in **7h**. As for the complex **7d·Zn(II)**, these atoms did not seem to be involved in the coordination of the metal, considering that no significative shifts were observed for the adjacent hydrogen atoms.

**Figure 4:**
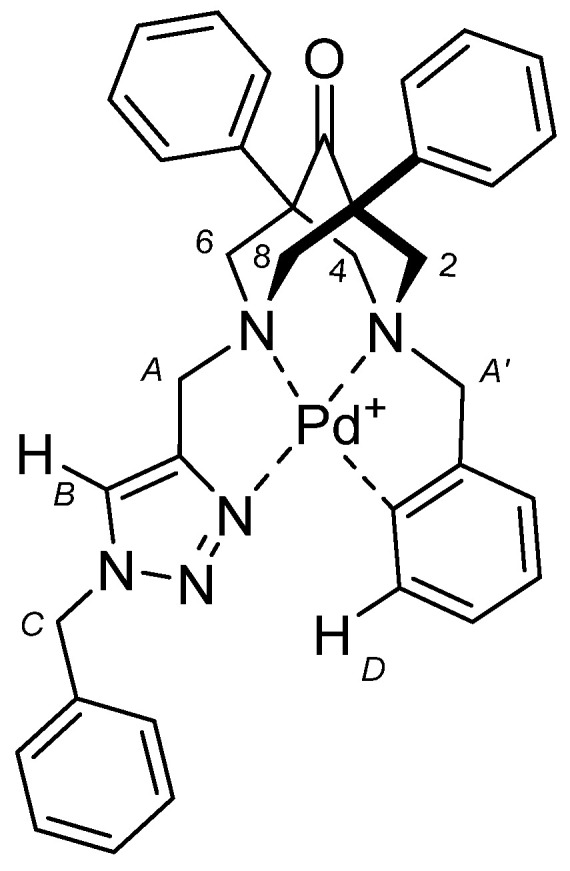
Coordination geometry of ligand **8a** complexed with Pd(II).

**Figure 5:**
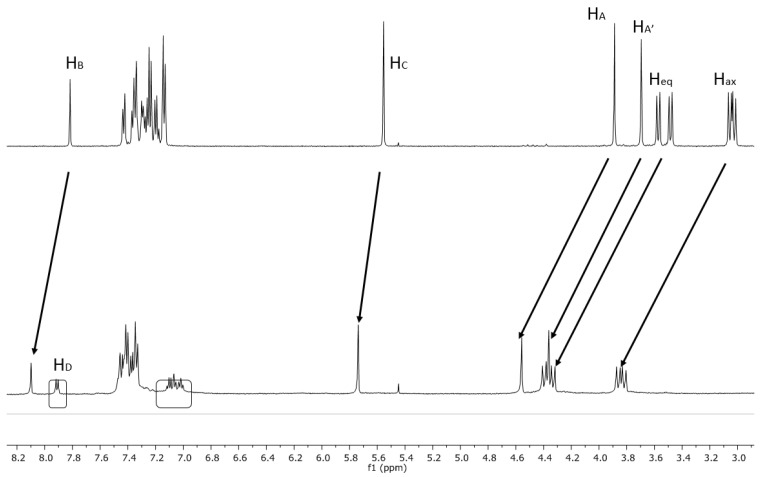
^1^H-NMR spectrum of compound **8a** and its Pd(II) complex **8a·Pd(II)**. The benzyl aromatic portion of the complex is highlighted in squares.

**Figure 6:**
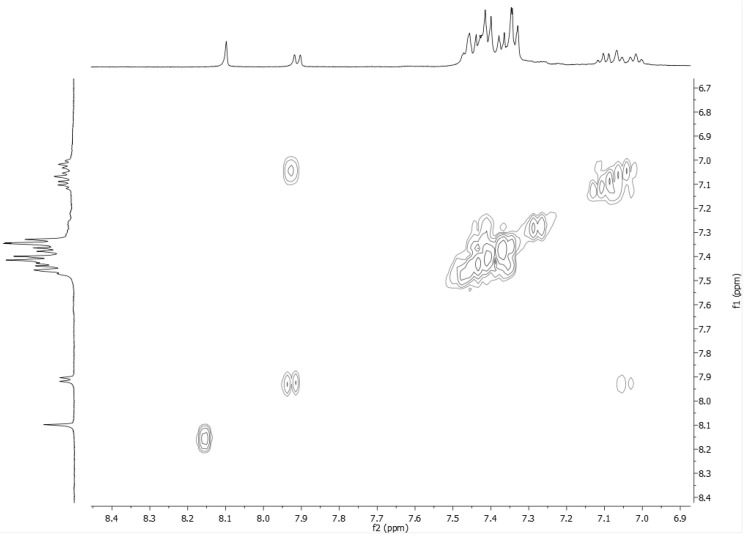
Aromatic portion of the ^1^H-^1^H COSY of compound **8a·Pd(II)**.

Concerning the mono-substituted triazole-bispidines, the complexation of bispidine **8a** with PdCl_2_ was investigated (Figure 4, Table 3). In this case, we were interested in exploring the possibility of obtaining a so-called ‘Pincer-like’ complex through the formation of a C–Pd bond with the aromatic ring of the benzyl residue. This kind of complexes have been previously reported by Bulygina et al. [20]. Worth notice is that the palladacycles are stable compounds, characterizable by NMR, and are efficient catalysts in C–C bond-forming reactions. The complexation reaction of **8a** with PdCl_2_ was performed in acetonitrile at room temperature for 48 h. The obtained solid **8a·Pd(II)** was characterized by ^1^H-NMR in CD_3_CN, similar to the previous ones (Figure 5). As observed for the other ligands, all the signals of the hydrogen atoms adjacent to the chelating centers were shifted upfield as a consequence of the formation of the complex (Table 3). The hydrogens neighboring the bispidine nitrogen atoms showed the highest shifts (∆δ = 0.66–0.84 ppm). The presence of the newly formed C–Pd bond was confirmed by the fact that in the spectrum of the complex, a doublet appeared at 7.91 ppm (J = 7.5 Hz, 1H). As indicated by the COSY spectrum (Figure 6), this signal correlates with a group of peaks at 7.00–7.12 ppm (a doublet and two triplets, J = 7.0–7.5 Hz), typical of an ortho-disubstituted aromatic spin system. These data suggest the formation of a C–Pd bond in the ortho position of the benzyl substituent, with the proton in ortho to the palladium center shifted downfield to 7.91 ppm as a doublet; conversely, the other three hydrogen atoms of the phenyl ring are shifted upfield. These observations are in agreement with what previously reported in the literature for similar benzyl bispidine–palladium complexes [21].

### 2.3. ESI-MS Studies on the Coordination Chemistry

**Figure 7:**
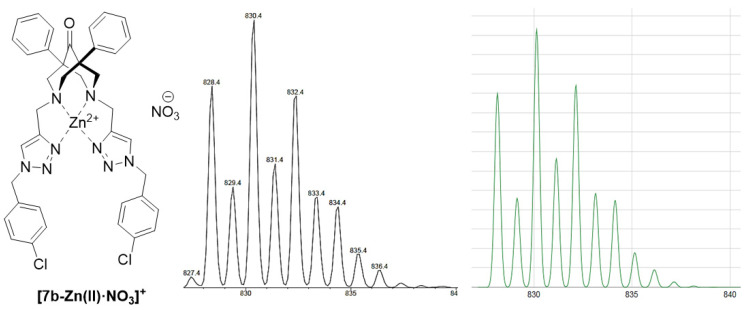
Comparison of the experimental ESI-MS spectrum of compound **[7b·Zn(II)·NO_3_]^+^** (**left**) and the simulation of the isotopic pattern for the elemental composition [C_39_H_36_Cl_2_N_9_O_4_Zn]^+^ (**right**).

**Figure 8:**
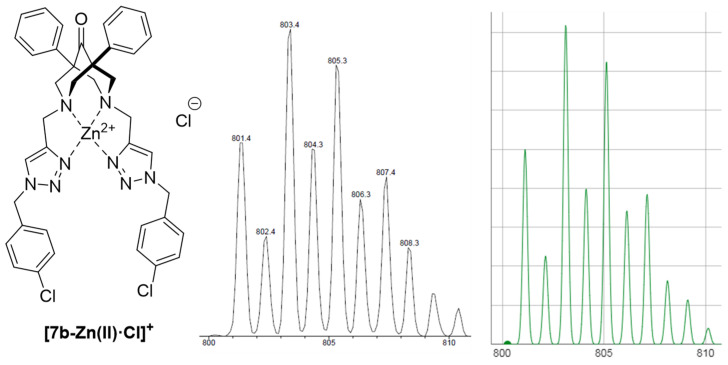
Comparison of the experimental ESI-MS spectrum of compound **[7b·Zn(II)·Cl]^+^** (**left**) and the simulation of the isotopic pattern for the elemental composition [C_39_H_36_Cl_3_N_8_OZn]^+^ (**right**).

With regard to the zinc-based complexes, further items of information were collected from electrospray ionization mass spectrometry (ESI-MS) analyses. The spectrum of compound **7b·Zn(II)** (Figure 7, left) showed the presence of one main signal as a complex pattern composed of many peaks, with the base peak at *m*/*z* 830.4, which is consistent with the elemental composition [C_39_H_36_Cl_2_N_9_O_4_Zn]^+^, assignable to the complex [**7b·Zn(II)·NO_3_]^+^**. This observation was confirmed by the comparison of the experimental pattern with the simulated isotopic pattern (Figure 7, right). Other much less intense peaks were observed due to anion exchange during the electro nebulization process. For example, a pattern with the base peak at *m*/*z* 803.4, consistent with [C_39_H_36_Cl_3_N_8_OZn]^+^, was ascribed to the formation of the [**7b·Zn(II)·Cl]^+^** cation (Figure 8).

Similar patterns were observed for the complexes **[7d·Zn(II)·NO_3_]^+^**, [C_37_H_36_N_11_O_4_Zn]^+^, base peak at *m*/*z* 762.22., and **[7d·Zn(II)·Cl]^+^**, [C_37_H_36_ClN_10_OZn]^+^, base peak at *m*/*z* 735.21. The spectra and the simulation of the isotopic patterns, consistent with the elemental compositions reported above, are shown in Figures S30 and S31, respectively.

The ESI-MS analysis was also crucial to confirm the formation of the Pincer-like complex for **8a·Pd(II)**. In this case, the base peak was found at *m*/*z* 658.5. The observed value was consistent with the elemental composition [C_36_H_34_N_5_OPd]^+^, corresponding to the monocationic complex. Again, a very good agreement between the experimental and the predicted peak pattern was observed (Figure S32).

### 2.4. Single-Crystal X-ray Characterization of the Free Ligand ***8a***

Among the synthesized products, we analyzed ligand **8a** by means of SC-XRD to provide an unambiguous description of its structure and derive useful information on the spatial arrangement of the N-benzyl triazole moiety with respect to the bispidine core. This feature influences the chiral space around the metal, determining the chelating capabilities of the ligand. These data helped us to rationalize the behavior of the compounds, also considering that the stereochemistry of these molecules is a major determinant of their chelating properties.

**Figure 9:**
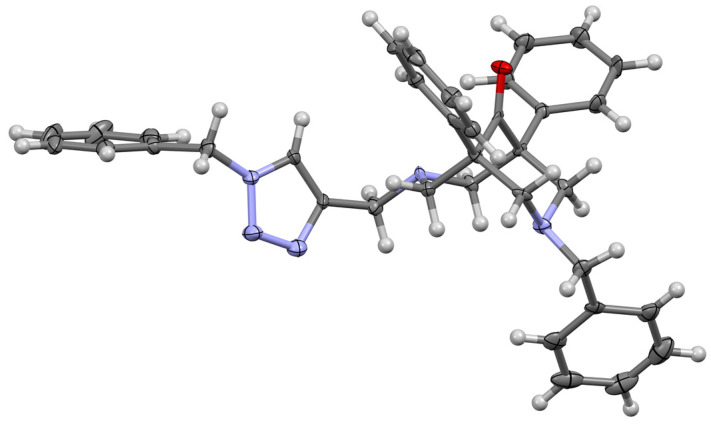
Thermal ellipsoid representation (40% probability) of one of the symmetrically independent molecules (**I**) in the crystal cell of **8a**. Color code: C = grey; H = white; N = blue; O = red.

Compound **8a** crystallized in the monoclinic space group Cc, with four crystallographically independent molecules (**I**, **A**, **B**, and **C**) related by pseudosymmetry in the asymmetric unit (Z′ = 4); conformer **I** is represented in Figure 9. Low-temperature (150 K) data were found to be pseudo-merohedrally twinned via twin law −1 0 0, 0 −1 0, 1 0 1, detected by the TwinRotMat routine in PLATON [22].

In the crystal, the conformation of the bispidine core was the same in all four independent molecules, whereas the benzyl substituent of the triazole ring was differently oriented. The similarity between the four molecules was calculated by using the “Molecule Overlay” routine in Mercury [23]: the RMSD for the non-hydrogen atoms of molecules **I** and **B** was 0.1589 Å, whereas for molecules **A** and **C**, a smaller value of 0.1454 Å was observed. The bispidine was in boat–chair conformation, with the portion bearing the triazole moiety in boat conformation, the two phenyl groups arranged in anti-geometry, and the benzyl ring in endo position. This finding was in agreement with our previous results [6], which indicated that the introduction of large substituents on the nitrogen promotes a boat conformation of the piperidine. The puckering parameters of the ring in chair conformation were: Q_T_ = 0.629(3) Å, φ = −151(3)°, and ϑ = 174(3)°. Conversely, the piperidine in boat conformation showed the following puckering parameters: Q_T_ = 2.442(3) Å, φ = −34.6(2)°, and ϑ = 156(1)° [24]. The crystal packing was consolidated by van der Waals contacts and weak Cπ-H···O-type interactions. Notably, the intermolecular contacts did not influence the conformation of the bicycle because the aromatic moieties were not involved in stacking interactions.

### 2.5. Molecular Modeling

**Figure 10:**
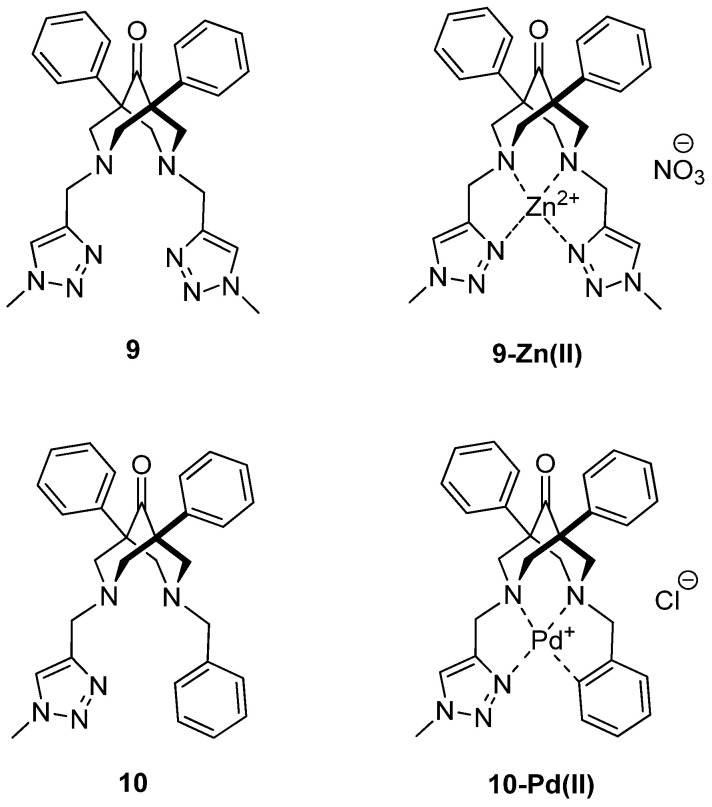
Model molecules **9** and **10** designed for conformational studies in silico.

To collect more information on the structure of both the ligands and the complexes, a computational study was performed. Firstly, the role of the triazole rings on the conformational behavior of the bispidine was considered. To this end, the model molecules **9** and **10** (Figure 10) were used to shorten the computational times, with the approximation that the residues on the triazole ring would not significantly affect the conformation of the fused piperidine rings of the bispidine core. Three possible arrangements of this core can exist, namely the chair–chair (cc), the boat–chair (bc), and the boat–boat (bb) conformations. A conformational analysis (Monte Carlo search with Molecular Mechanics energy minimization) was performed; the most favorable conformers were then optimized at the DFT-B3LYP/6-311G(d) level in vacuo.

To obtain more information on the coordination capability of the ligands, the complexes **9·Zn(II)** and **10·Pd(II)** were also submitted to energy optimization at the DFT-B3LYP/LANL2DZ level. In both cases, the presence of the anion (nitrate and chloride anion, respectively) in the first coordination sphere was also considered.

**Figure 11:**
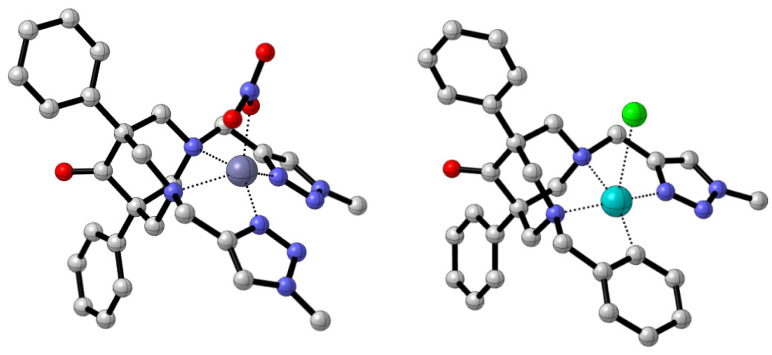
Optimized structures of **9·Zn(II)** and **10·Pd(II)** complexes. Color code: C = grey; O = red; N = blue; Cl = green; Zn = violet; Pd = light blue.

In compound **9·Zn(II)**, the four coordinating nitrogen atoms lay on the same plane, forming a square of about 3 Å, with the Zn-N bond lengths in the range 2.1–2.3 Å (Figure 11). The metal ion occupies the apex of the resulting square-based pyramid, and the angles of inclination of this pyramid are in the range of 17–21°. The nitrate anion is 2.1 Å far from the metal. Similarly, for **10·Pd(II)** a square-based pyramid was found (Figure 11), even if in this case the angle of inclination was only 8.2°, very close to a square planar geometry. The chloride anion is at 3.0 Å from the palladium ion, and the C–Pd bond length is 2.0 Å. The Pd-N bond lengths vary from 2.1 to 2.3 Å. In both structures, the bispidine skeleton is bent towards the opposite side of the apex of the pyramid.

These results are in agreement with the experimental data gathered by NMR, MS, and SC-XRD. Most notably, the modeled conformation of the complexes supported the chelation number definitions resulting from the NMR investigations.

### 2.6. Catalysis Applications of the Complexes

**Scheme 5:**
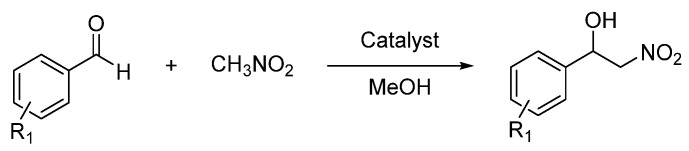
Scheme of a general Henry reaction.

Considering the extraordinary chelation capability of our library of triazole bispidines, tests were performed to investigate their possible use as catalysts. In detail, we focused on the Henry reaction for the copper- and zinc-based compounds. The Henry reaction is a very useful C–C bond-forming reaction, consisting in the nucleophilic addition of a nitronate (the carbanion of a nitroalkane) to an electrophilic aldehyde or ketone [25]. The main product is a β-nitro alcohol (Scheme 5), which can be further transformed into amino-alcohols, useful for the synthesis of many drugs and natural products [26,27].

The reaction occurs under basic conditions, or with the assistance of an organometallic catalyst. One of the most commonly used metals is copper, combined with different amino ligands [28]. More recently, the use of zinc in combination with nitrogen-based ligands has been demonstrated to be a valid alternative, with the great advantage of the much lower toxicity of this metal [29].

Ligand **7a** was thus selected as the reference compound for testing the reaction conditions, in combination with both zinc and copper as metals. The reactions were initially performed in ethanol at room temperature for 24 h with 5 equivalents of nitromethane with respect to the aldehyde. Different loadings of catalyst were thus screened, as well as the influence of the addition of triethylamine in the reaction mixture (Table 4, entries 1–4). Notably, the zinc complex with the bispidine proved to be active at only 2% mol loading of the catalyst, with a 79% conversion for p-nitrobenzaldehyde (Table 4, entry 2). The complete conversion was achieved with 15% mol of catalyst. The addition of triethylamine caused a reduction of the yield (Table 4, entry 4). This effect was probably due to triethylamine coordinating the catalyst, thus inhibiting its activity. It is important to underline that the copper-based complex proved to be less efficient in the same conditions, with only a 22% conversion for p-nitrobenzaldehyde, when the catalyst was used at 2% mol, and a 56% conversion instead of completeness when employed at 15% mol (Table 4, entries 5 and 6, respectively).

Later on, the best set-up was investigated to shorten the reaction time, testing classical heating against microwave irradiation. Under the optimized reaction conditions, namely **7a·Zn** 15% mol, 80 °C in MeOH for 1 h under microwave irradiation, a screening was performed using different aromatic aldehydes as substrates to exploit the scope of the reaction. The best results (Table 4, entries 7–11) were obtained with electron-poor aldehydes, whereas aliphatic and EDG aldehydes led to unsatisfactory yields.

**Scheme 6:**
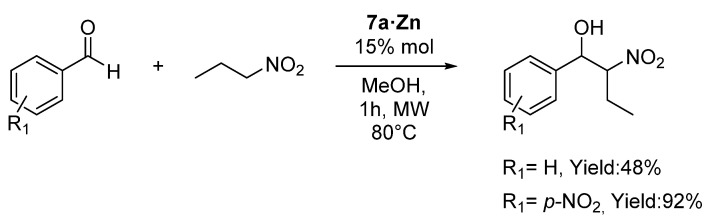
Henry reaction with nitropropane. Conditions: aldehyde (1 eq), nitropropane (5 eq), **7a·Zn** (15% mol), methanol (0.2 M), 1 h, 80 °C, MW irradiation.

Most notably, the efficiency of this reaction set-up was also retained with different nitro-compounds; for instance, when nitropropane was used instead of nitromethane in the best conditions (Table 4, entry 3), the product was achieved in good yield with the same substrate (Scheme 6).

## 3. Conclusions

The [3.3.1]-diazabicyclo core of bispidines plays a crucial role in defining their properties as ligands. Moreover, the substituents bound to it greatly influence their effectiveness as coordinating agents for various metal cations. In this work, a library of triazole bispidines bearing diverse substituents was designed and synthesized to explore additional chelation points with respect to known bispidines. In order to investigate the coordination chemistry of these distinct systems with different metals, the results obtained from structural, spectroscopic, and computational methods were compared, revealing a notable consistency between experimental observations and theoretical calculations. As expected from our previous work [6], where the presence of pyridine rings strongly stabilized the formation of metal complexes with the bispidine, the introduction of the triazole ring enables a strong coordination. Nevertheless, the further addition of pyridines to the triazole rings does not promote further coordination in those positions, as the pyridine nitrogens are distant from the chelation center. Interesting chelating properties were discovered for mono-triazole bispidines; in this respect, SC-XRD data of 8a allowed to obtain a 3D picture of the ligands in the solid state. The metal complexes were deeply characterized using ESI-MS spectrometry to calculate the molecular mass, and NMR titration to verify the formation of the complex in solution as well as its stoichiometry. The catalytic ability of the metal complexes was investigated in established C-C bond-forming reactions; in detail, the Henry reaction achieved excellent results with zinc-based bispidines as catalysts under microwave irradiation.

## 4. Materials and Methods

### 4.1. General Remarks

Chemicals and solvents were purchased from Merck KGaA (Darmstadt, Germany) and used without further purifications. Microwave reactions were conducted in a Biotage^®^ Initiator+ (Uppsala, Sweden). Reactions were monitored mostly by thin-layer chromatography (TLC), performed on Merck Kieselgel 60 F_254_ plates (Merck KGaA, Darmstadt, Germany). Visualization was accomplished by UV irradiation at 254 nm and subsequently by treatment with the alkaline KMnO_4_ reactant or with a phosphomolybdic reagent. GC-MS analyses were performed on an Agilent HP 6890 gas chromatograph equipped with a HP-5MS column (30 m × 0.25 mm × 0.25 µm), with injection temperature = 250 °C, injecting 1 µL of solution, and using He as a carrier gas (1.0 mL·min^−1^). The method used for the analyses was: 60 °C (1 min)/6 °C/min/150 °C (1 min)/12 °C/min/280 °C (5 min). NMR spectra were recorded (^1^H-NMR, 400 MHz; ^13^C-NMR, 101 MHz) on a Bruker 400 spectrometer (Bruker Italia Srl, Milan, Italy) in deuterated solvents, using TMS as an internal standard; chemical shifts (δ) in the spectra are reported in ppm, the coupling constants *J* are reported in Hz. The ESI-MS spectra were recorded on a Bruker Esquire 3000 plus spectrometer (Bruker Italia Srl, Milan, Italy), whereas the melting points were measured on a Reichert apparatus, equipped with a Reichert microscope. Elemental analyses were performed on a Costech ECS mod.4010 instrument (Costech Analytical Technologies, Inc., Valencia, CA, USA).

### 4.2. Synthetic Procedures

**1,5-Diphenyl-3,7-di(prop-2-yn-1-yl)-3,7-diazabicyclo[3.3.1]nonan-9-one (1)**

Compound **3** (1 eq., 0.1 M) was dissolved in acetone with an excess of sodium carbonate (5 eq.) as a heterogeneous inorganic base; the mixture was cooled to 0 °C and 2.5 eq. of propargyl bromide was added dropwise under stirring. The reaction was left stirring at room temperature for 24 h. The solid was filtered off on a Buchner funnel, and the filtrate was dried under vacuum, affording the desired product **1**. Yield 95%. Yellow oil. ^1^H-NMR (400 MHz, CDCl_3_) δ (ppm) 7.43–7.14 (m, 10H, Ar-H), 3.61 (d, *J* = 10.8 Hz, 4H, CH_2_-eq), 3.55 (d, *J* = 2.4 Hz, 4H, CH_2_-propargyl), 3.32 (d, *J* = 10.8 Hz, 4H, CH_2_-ax), 2.29 (t, *J* = 2.4 Hz, 2H, CH-propargyl). ^13^C-NMR (101 MHz, CDCl_3_) δ (ppm) 209.9 (1C, C=O), 142.3 (2C, Ph-*ipso*), 127.9 (4C, Ar), 126.9 (4C, Ar), 126.7 (2C, Ar), 78.2 (2C, propargyl), 73.7 (2C, propargyl), 64.0 (4C, CH_2_-bispidine), 54.0 (2C, C-Ph bispidine), 46.4 (2C, CH_2_-propargyl). Elemental analysis: C_25_H_24_N_2_O: (368.48); calculated (%): C, 81.49; H, 6.57; N, 7.60; found (%): C, 81.98 H, 6.97; N, 7.23.

**3,7-Dibenzyl-1,5-diphenyl-3,7-diazabicyclo[3.3.1]nonan-9-one (2)**

Benzylamine (2 eq.) was dissolved in methanol and cooled to 0 °C. Acetic acid (2% vol/vol), 1,3-diphenyl-2-propanone (1 eq., 0.2 M) and paraformaldehyde (4 eq.) were added, and the mixture was refluxed for 12 h. The yellow solution was cooled to room temperature before filtering the white precipitate on a Buchner funnel and washing it with cold methanol. The pure product **2** was obtained without any further purification. Yield 84%. White solid. ^1^H-NMR (400 MHz, CDCl_3_) δ (ppm) 7.44–7.11 (m, 20H, Ar-H), 3.73 (s, 4H, CH_2_-Bn), 3.54 (d, *J* = 10.8 Hz, 4H, CH_2_-eq), 3.12 (d, *J* = 10.8 Hz, 4H, CH_2_-ax). All the other data are in agreement with the literature (see Supplementary Materials for references).

**1,5-Diphenyl-3,7-diazabicyclo[3.3.1]nonan-9-one (3) and 3-benzyl-1,5-diphenyl-3,7-diazabicyclo[3.3.1]nonan-9-one (4)**

Compound **2** (2 g) was dissolved in ethyl acetate (100 mL). A catalytic amount of Pd/C was added to the reaction, and the mixture was stirred for 120 h at room temperature under hydrogen atmosphere. The work-up consisted of a filtration through a celite pad to remove the catalyst and washing with cold ethyl acetate and methanol. The liquid layer was collected in a round bottom flask to remove the solvent under vacuum. The crude was purified through column chromatography, starting from 1% of methanol in ethyl acetate, to 80% of methanol, to give the two compounds **3** and **4**.

**3:** Yield 63%. White solid. ^1^H-NMR (400 MHz, CDCl_3_) δ (ppm) 7.39–7.22 (m, 10H, Ar-H), 3.88 (d, *J* = 12.3 Hz, 4H, CH_2_-eq), 3.71 (d, *J* = 12.1 Hz, 4H, CH_2_-ax), 3.14 (br s, 2H, NH). All the other data are in agreement with the literature (see Supplementary Materials for references).

**4:** Yield 25%. Yellow oil. ^1^H-NMR (400 MHz, CDCl_3_) δ (ppm) 7.37–7.17 (m, 15H, Ar-H), 3.77 (d, *J* = 13.9 Hz, 2H, CH_2_-eq), 3.59–354 (m, 7H, CH_2_-eq, CH_2_-Bn, CH_2_-ax, NH), 3.11 (d, *J* = 13.9 Hz, 2H, CH_2_-ax). ^13^C-NMR (101 MHz, CDCl_3_) δ (ppm) 208.5 (1C, C=O), 136.7 (2C, Ph-*ipso*), 136.3 (1C, PhBn-*ipso*), 127.8 (2C, Ar), 127.3 (2C, Ar), 127.0 (4C, Ar), 126.7 (2C, Ar), 126.3 (2C, Ar), 126.2 (1C, Ar), 64.9 (2C, CH_2_-bispidine), 61.7 (2C, CH_2_-bispidine), 61.2 (2C, CH_2_-Bn), 54.6 (2C, C-Ph bispidine). Elemental analysis: C_26_H_26_N_2_O: (382.51); calculated (%): C, 81.64; H, 6.85; N, 7.32; found (%): C, 81.21; H, 6.44; N, 7.62.

**3-Benzyl-1,5-diphenyl-7-(prop-2-yn-1-yl)-3,7-diazabicyclo[3.3.1]nonan-9-one (5)**

Compound **4** (1 eq., 0.1 M) was dissolved in acetone with an excess of sodium carbonate (5 eq.) as a heterogeneous inorganic base; the mixture was cooled to 0 °C, and 1.5 eq. of propargyl bromide were added dropwise under stirring. The reaction was left stirring at room temperature for 24 h. The solid was filtered off on a Buchner funnel, and the filtrate was dried under vacuum, affording the desired product **5**. Yield 82%. Yellow oil. ^1^H-NMR (400 MHz, CDCl_3_) δ (ppm) 7.49–7.16 (m, 15H, Ar-H), 3.72 (s, 2H, CH_2_-Bn), 3.68 (d, *J* = 10.8 Hz, 2H, CH_2_-eq), 3.58 (d, *J* = 2.4 Hz, 2H, CH_2_-propargyl), 3.54 (d, *J* = 10.9 Hz, 2H, CH_2_-eq), 3.27 (d, *J* = 10.8 Hz, 2H, CH_2_-ax), 3.15 (d, *J* = 10.9 Hz, 2H, CH_2_-ax), 2.34 (t, *J* = 2.4 Hz, 1H, CH-propargyl). ^13^C-NMR (101 MHz, CDCl_3_) δ (ppm) 210.4 (1C, C=O), 142.8 (2C, Ph-*ipso*), 139.6 (1C, PhBn-*ipso*), 128.7 (2C, Ar), 128.4 (2C, Ar), 127.7 (4C, Ar), 127.2 (2C, Ar), 127.0 (4C, Ar), 78.7 (1C, propargyl), 74.2 (1C, propargyl), 64.5 (2C, CH_2_-bispidine), 64.3 (2C, CH_2_-Bn), 61.6 (2C, CH_2_-bispidine), 54.3 (2C, C-Ph bispidine), 46.8 (1C, CH_2_-propargyl). Elemental analysis: C_29_H_28_N_2_O: (420.56); calculated (%): C, 82.82; H, 6.71; N, 6.66; found (%): C, 82.21; H, 6.48; N, 6.62.

**General procedure for the synthesis of azides (6a**–**6j)**

An excess of sodium azide (15 eq.) was added to a mixture of alkyl or benzyl bromide (1 eq., 0.2 M) in *N*,*N*-dimethylformamide (DMF) and reacted under microwave irradiation at 100 °C for 15 min. The crude was extracted from ethyl acetate and water (5 times); the organic phase was dried over anhydrous Na_2_SO_4_, filtered, and the liquid evaporated under vacuum to afford the azido-derivatives **6a**–**j** in quantitative yields.

**6a: (Azidomethyl)benzene.** ^1^H-NMR (400 MHz, CDCl_3_) δ (ppm) 7.34–7.19 (m, 5H, Ar), 4.27 (s, 2H, CH_2_). All the other data are in agreement with the literature (see Supplementary Materials for references).

**6b: 1-(Azidomethyl)-4-chlorobenzene.** ^1^H-NMR (400 MHz, CDCl_3_) δ (ppm) 7.35 (d, *J* = 8.4 Hz, 2H, Ar), 7.24 (d, *J* = 8.4 Hz, 2H, Ar), 4.30 (s, 2H, CH_2_). All the other data are in agreement with the literature (see Supplementary Materials for references).

**6c: 1-(Azidomethyl)-4-nitrobenzene.** ^1^H-NMR (400 MHz, CDCl_3_) δ (ppm) 8.25 (d, *J* = 8.8 Hz, 2H, Ar), 7.51 (d, *J* = 8.8 Hz, 2H, Ar), 4.51 (s, 2H, CH_2_). All the other data are in agreement with the literature (see Supplementary Materials for references).

**6d: 2-(Azidomethyl)pyridine.** ^1^H-NMR (400 MHz, CDCl_3_) δ (ppm) 8.61 (d, *J* = 4.3 Hz, 1H, pyr), 7.72 (td, *J* = 7.7, 1.8 Hz, 1H, pyr), 7.35 (d, *J* = 7.8 Hz, 1H, pyr), 7.24 (d, 1H, pyr), 4.49 (s, 2H, CH_2_). All the other data are in agreement with the literature (see Supplementary Materials for references).

**6e: 4-(Azidomethyl)pyridine.** ^1^H-NMR (400 MHz, CDCl_3_) δ (ppm) 8.63 (d, *J* = 7.5 Hz, 2H, pyr), 7.26 (d, *J* = 7.5 Hz, 2H, pyr), 4.41 (s, 2H, CH_2_). All the other data are in agreement with the literature (see Supplementary Materials for references).

**6f**:**1-Azidooctane.** ^1^H-NMR (400 MHz, CDCl_3_) δ (ppm) 3.25 (t, *J* = 7.0 Hz, 2H, CH_2_-N_3_), 1.63–1.35 (m, 2H, alk), 1.33–1.27 (m, 10H, alk), 0.89 (t, *J* = 6.8 Hz, 3H, CH_3_). All the other data are in agreement with the literature (see Supplementary Materials for references).

**6g: 1-Azidoundecane.** ^1^H-NMR (400 MHz, CDCl_3_) δ (ppm) 3.24 (t, *J* = 7.0 Hz, 2H, CH_2_-N_3_), 1.65–1.53 (m, 2H, alk), 1.40–1.21 (m, 16H, alk), 0.89 (t, *J* = 6.8 Hz, 3H, CH_3_). All the other data are in agreement with the literature (see Supplementary Materials for references).

**6h: 3-Azidopropan-1-ol.** ^1^H-NMR (400 MHz, CDCl_3_) δ (ppm) 3.73 (m, 2H, CH_2_-OH), 3.44 (t, *J* = 7.4 Hz, 2H, CH_2_-N_3_), 2.49 (br s, 1H, OH), 1.84 (q, *J* = 7.4 Hz, 2H, CH_2_). All the other data are in agreement with the literature (see Supplementary Materials for references).

**6i: 1,2-Bis(azidomethyl)benzene.** ^1^H-NMR (400 MHz, CDCl_3_) δ (ppm) 7.37 (s, 4H, Ar-H), 4.43 (s, 4H, CH_2_-N_3_). All the other data are in agreement with the literature (see Supplementary Materials for references).

**6j: 1,6-Diazidohexane.** ^1^H-NMR (400 MHz, CDCl_3_) δ (ppm) 3.24 (t, *J* = 7.4 Hz, 4H, CH_2_-N_3_), 1.57–1.48 (m, 4H, alk), 1.36–1.31 (m, 4H, alk). All the other data are in agreement with the literature (see Supplementary Materials for references).

**General procedure for the synthesis of bis-triazole-bispidines (7a–h)**

Compound **1** (1 eq., 0.1 M) was dissolved in a 1:1 acetone-water mixture. Then, the appropriate azide (**6a**–**h**) (2.1 eq.), CuSO_4_ (0.02 eq.), and sodium ascorbate (0.2 eq.) were added in sequence. The reaction was performed for 1 h under microwave (MW) irradiation at 50 °C. Then, acetone was removed under vacuum, and ethyl acetate was added to the aqueous medium to extract the desired compound in the organic phase. The complete removal of the copper catalyst was accomplished by washing the organic phase with a 1 M aqueous solution of ethylenediaminetetraacetic acid (EDTA). The organic phase was dried over anhydrous Na_2_SO_4_, filtered, and the liquid evaporated under vacuum. With this protocol, the desired product (**7a**–**h**) was obtained in good yield (73–95% yields, see Table 1).

**7a: 3,7-Bis((1-benzyl-1*H*-1,2,3-triazol-4-yl)methyl)-1,5-diphenyl-3,7-diazabicyclo[3.3.1]nonan-9-one.** Yield 95%. White solid. ^1^H-NMR (400 MHz, CDCl_3_) δ (ppm) 7.49 (s, 2H, triazole-H), 7.40–7.08 (m, 20 H, Ar-H), 5.52 (s, 4H, CH_2_-Bn), 3.87 (s, 4H, CH_2_-Bn), 3.50 (d, *J* = 10.8 Hz, 4H, CH_2_-eq), 3.13 (d, *J* = 10.8 Hz, 4H, CH_2_-ax). ^13^C-NMR (101 MHz, CDCl_3_) δ (ppm) 210.5 (1C, C=O), 145.1 (2C, triazole-*ipso*), 142.2 (2C, Ph-*ipso*), 135.5 (2C, Bn-*ipso*), 134.9 (2C, Ar), 129.3 (2C, Ar), 128.9 (2C, Ar), 128.8 (2C, Ar), 128.4 (2C, Ar), 128.3 (2C, Ar), 128.1 (2C, Ar), 128.0 (2C, Ar), 126.9 (2C, Ar), 126.8 (2C, Ar), 122.7 (2C, CH-triazole), 64.8 (4C, CH_2_-bispidine), 54.3 (4C, CH_2_-triazole + CH_2_-Bn), 52.6 (2C, C-Ph bispidine). Elemental analysis: C_39_H_38_N_8_O: (634.79); calculated (%): C, 73.79; H, 6.03; N, 17.65; found (%): C, 73.02; H, 5.97; N, 18.23; melting point: 271 °C.

**7b: 3-((1-(3-Chlorobenzyl)-1*H*-1,2,3-triazol-4-yl)methyl)-7-((1-(4-chlorobenzyl)-1*H*-1,2,3-triazol-4-yl)methyl)-1,5-diphenyl-3,7-diazabicyclo[3.3.1]nonan-9-one.** Yield 75%. White solid. ^1^H-NMR (400 MHz, CDCl_3_) δ (ppm) 7.52 (s, 2H, triazole-H), 7.36–7.08 (m, 18 H, Ar-H), 5.50 (s, 4H, CH_2_-Bn), 3.88 (s, 4H, CH_2_-Bn), 3.51 (d, *J* = 10.7 Hz, 4H, CH_2_-eq), 3.13 (d, *J* = 10.7 Hz, 4H, CH_2_-ax). ^13^C-NMR (101 MHz, CDCl_3_) δ (ppm) 210.4 (1C, C=O), 145.3 (2C, triazole-*ipso*), 142.1 (2C, Ph-*ipso*), 134.9 (2C, Bn-*ipso*), 133.4 (2C, Ar-Cl), 129.5 (4C, Ar), 129.4 (4C, Ar), 128.0 (4C, Ar), 126.9 (4C, Ar), 126.8 (2C, Ar), 122.7 (2C, CH-triazole), 64.9 (4C, CH_2_-bispidine), 54.4 (2C, CH_2_-triazole) 53.5 (2C, CH_2_-Bn), 52.6 (2C, C-Ph bispidine). Elemental analysis: C_39_H_36_Cl_2_N_8_O: (703.67); calculated (%): C, 66.57; H, 5.16; N, 15.92; found (%): C, 67.00; H, 5.55; N, 16.21; melting point: 244 °C.

**7c: 3-((1-(3-Nitrobenzyl)-1*H*-1,2,3-triazol-4-yl)methyl)-7-((1-(4-nitrobenzyl)-1*H*-1,2,3-triazol-4-yl)methyl)-1,5-diphenyl-3,7-diazabicyclo[3.3.1]nonan-9-one.** Yield 78%. White solid. ^1^H-NMR (400 MHz, CDCl_3_) δ (ppm) 8.21 (d, *J* = 8.7 Hz, 4H, *o*-NO_2_Ar), 7.64 (s, 2H, triazole-H), 7.41 (d, *J* = 8.6 Hz, 4H, *m*-NO_2_Ar), 7.31–7.10 (m, 10H, Ar-H), 5.67 (s, 4H, CH_2_-Bn), 3.92 (s, 4H, CH_2_-Bn), 3.57 (d, *J* = 10.4 Hz, 4H, CH_2_-eq), 3.16 (d, *J* = 10.4 Hz, 4H, CH_2_-ax). ^13^C-NMR (101 MHz, CDCl_3_) δ (ppm) 210.2 (1C, C=O), 148.1 (2C, Ar-NO_2_), 145.5 (2C, triazole-*ipso*), 141.8 (4C, Ph-*ipso* + Bn-*ipso*), 128.5 (2C, Ar), 127.9 (4C, Ar), 126.9 (4C, Ar), 126.8 (4C, Ar), 124.3 (4C, Ar), 123.0 (2C, CH-triazole), 64.8 (4C, CH_2_-bispidine), 54.3 (2C, CH_2_-triazole) 53.1 (2C, CH_2_-Bn), 52.4 (2C, C-Ph bispidine). Elemental analysis: C_39_H_36_N_10_O_5_: (724.78); calculated (%): C, 64.63; H, 5.01; N, 19.33; found (%): C, 64.02; H, 4.87; N, 19.65; melting point: 267 °C.

**7d: 1,5-Diphenyl-3,7-bis((1-(pyridin-2-ylmethyl)-1*H*-1,2,3-triazol-4-yl)methyl)-3,7-diazabicyclo[3.3.1]nonan-9-one.** Yield 84%. White solid. ^1^H-NMR (400 MHz, CDCl_3_) δ (ppm) 8.58 (d, *J* = 4.3 Hz, 2H, CH-pyr), 7.73 (s, 2H, triazole-H), 7.65 (td, *J* = 7.7, 1.8 Hz, 2H, CH-pyr), 7.40–7.09 (m, 14H, Ar-H), 5.67 (s, 4H, CH_2_-Bn), 3.92 (s, 4H, CH_2_-Bn), 3.54 (d, *J* = 10.4 Hz, 4H, CH_2_-eq), 3.15 (d, *J* = 10.4 Hz, 4H, CH_2_-ax). ^13^C-NMR (101 MHz, DMSO-d_6_) δ (ppm) 211.2 (1C, C=O), 155.7 (2C, pyridine-*ipso*), 149.8 (2C, pyr), 143.9 (2C, triazole-*ipso*), 143.4, (2C, Ph-*ipso*), 128.2 (2C, Ar), 127.2 (4C, Ar), 126.8 (4C, Ar), 125.3 (2C, Ar), 123.6 (4C, Ar), 122.3 (2C, CH-triazole), 64.4 (4C, CH_2_-bispidine), 54.8 (2C, CH_2_-triazole) 54.5 (2C, CH_2_-Bn), 51.9 (2C, C-Ph bispidine). Elemental analysis: C_37_H_36_N_10_O: (636.76); calculated (%): C, 69.79; H, 5.70; N, 22.00; found (%): C, 70.59; H, 6.03; N, 22.14; melting point: 241 °C.

**7e: 1,5-Diphenyl-3,7-bis((1-(pyridine-4-ylmethyl)-1*H*-1,2,3-triazol-4-yl)methyl)-3,7-diazabicyclo[3.3.1]nonan-9-one.** Yield 83%. White solid. ^1^H-NMR (400 MHz, CDCl_3_) δ (ppm) 8.57 (dd, *J* = 4.5, 1.6 Hz, 2H, CH-pyr), 7.61 (s, 2H, triazole-H), 7.25–7.11 (m, 14H, Ar-H), 7.06 (d, *J* = 6.0 Hz, 2H, CH-pyr), 5.53 (s, 4H, CH_2_-Bn), 3.91 (s, 4H, CH_2_-Bn), 3.55 (d, *J* = 10.4 Hz, 4H, CH_2_-eq), 3.15 (d, *J* = 10.4 Hz, 4H, CH_2_-ax). ^13^C-NMR (101 MHz, CDCl_3_) δ (ppm) 210.2 (1C, C=O), 150.6 (4C, pyr), 145.4 (2C, pyridine-*ipso*), 143.7 (2C, triazole-*ipso*), 141.9 (2C, Ph-*ipso*), 127.9 (4C, Ar), 126.8 (6C, Ar), 123.1 (4C, Ar), 122.0 (2C, CH-triazole), 64.8 (4C, CH_2_-bispidine), 54.3 (2C, CH_2_-triazole) 52.7 (2C, CH_2_-Bn), 52.4 (2C, C-Ph bispidine). Elemental analysis: C_37_H_36_N_10_O: (636.76); calculated (%): C, 69.79; H, 5.70; N, 22.00; found (%): C, 70.53; H, 6.12; N, 22.11; melting point: 257 °C.

**7f: 3,7-Bis((1-octyl-1*H*-1,2,3-triazol-4-yl)methyl)-1,5-diphenyl-3,7-diazabicyclo[3.3.1]nonan-9-one.** Yield 86%. Yellow oil. ^1^H-NMR (400 MHz, CDCl_3_) δ (ppm) 7.56 (s, 2H, triazole-H), 7.29–7.15 (m, 10H, Ar-H), 4.35 (t, *J* = 7.3 Hz, 4H, alk-CH_2_-triazole), 3.91 (s, 4H, N-CH_2_-triazole), 3.56 (d, *J* = 10.8 Hz, 4H, CH_2_-eq), 3.16 (d, *J* = 10.8 Hz, 4H, CH_2_-ax), 1.98–1.82 (m, 4H, alk), 1.37–1.19 (m, 20H, alk), 0.86 (t, *J* = 6.7 Hz, 6H, alk). ^13^C-NMR (101 MHz, CDCl_3_) δ (ppm) 210.4 (1C, C=O), 144.5 (2C, triazole-*ipso*), 142.3 (2C, Ph-*ipso*), 127.8 (4C, Ar), 126.8 (4C, Ar), 126.6 (2C, Ar), 122.4 (2C, CH-triazole), 64.7 (4C, CH_2_-bispidine), 54.3 (2C, CH_2_-triazole), 52.5 (2C, C-Ph bispidine), 50.4 (2C, alk), 31.7 (2C, alk), 30.3 (2C, alk), 29.0 (2C, alk), 28.9 (2C, alk), 26.5 (2C, alk), 22.5 (2C, alk), 14.0 (2C, alk). Elemental analysis: C_41_H_58_N_8_O: (678.97); calculated (%): C, 72.53; H, 8.61; N, 16.50; found (%): C, 72.91; H, 8.11; N, 15.99.

**7g: 1,5-Diphenyl-3,7-bis((1-undecyl-1*H*-1,2,3-triazol-4-yl)methyl)-3,7-diazabicyclo[3.3.1]nonan-9-one.** Yield 73%. Yellow oil. ^1^H-NMR (400 MHz, DMSO-d_6_) δ (ppm) 8.13 (s, 2H, triazole-H), 7.36–7.07 (m, 10H, Ar-H), 4.35 (t, *J* = 6.9 Hz, 4H, alk-CH_2_-triazole), 3.85 (s, 4H, N-CH_2_-triazole), 3.50 (d, *J* = 10.8 Hz, 4H, CH_2_-eq), 3.03 (d, *J* = 10.7 Hz, 4H, CH_2_-ax), 1.82 (m, 4H, alk), 1.26–1.11 (m, 32H, alk), 0.85 (t, *J* = 6.9 Hz, 6H, alk). ^13^C-NMR (101 MHz, DMSO-d_6_) δ (ppm) 211.1 (1C, C=O), 143.8 (2C, triazole-*ipso*), 143.4 (2C, Ph-*ipso*), 128.1 (4C, Ar), 127.2 (4C, Ar), 126.7 (2C, Ar), 124.15 (2C, CH-triazole), 64.45 (4C, CH_2_-bispidine), 54.53 (2C, CH_2_-triazole), 52.02 (2C, C-Ph bispidine), 49.76 (2C, alk), 31.75 (2C, alk), 29.38 (6C, alk), 29.31 (2C, alk), 29.13 (2C, alk), 28.84 (2C, alk), 26.27 (2C, alk), 22.54 (2C, alk), 14.39 (2C, alk). Elemental analysis: C_47_H_70_N_8_O: (763.13); calculated (%): C, 73.97; H, 9.25; N, 14.68; found (%): C, 73.09; H, 8.99; N, 14.18.

**7h: 3,7-Bis((1-(3-hydroxypropyl)-1*H*-1,2,3-triazol-4-yl)methyl)-1,5-diphenyl-3,7-diazabicyclo[3.3.1]nonan-9-one.** Yield 85%. White solid. ^1^H-NMR (400 MHz, CDCl_3_) δ (ppm) 7.68 (s, 2H, triazole-H), 7.32–7.10 (m, 10H, Ar-H), 4.52 (t, *J* = 6.6 Hz, 4H, alk-CH_2_-triazole), 3.90 (s, 4H, N-CH_2_-triazole), 3.60–3.56 (m, 4H, -CH_2_-OH), 3.54 (d, *J* = 10.9 Hz, 4H, CH_2_-eq), 3.14 (d, *J* = 10.8 Hz, 4H, CH_2_-ax), 2.96 (br s, 2H, OH), 2.14–2.06 (m, 4H, alk-CH_2_). ^13^C- NMR (101 MHz, CDCl_3_) δ (ppm) 210.5 (1C, C=O), 144.5 (2C, triazole-*ipso*), 142.0 (2C, Ph-*ipso*), 128.0 (4C, Ar), 127.0 (4C, Ar), 126.9 (2C, Ar), 123.8 (2C, CH-triazole), 64.8 (4C, CH_2_-bispidine), 58.4 (2C, CH_2_-OH), 54.3 (2C, CH_2_-triazole), 52.4 (2C, C-Ph bispidine), 47.0 (2C, alk), 32.7 (2C, alk). Elemental analysis: C_31_H_38_N_8_O_3_: (570.70); calculated (%): C, 65.24; H, 6.71; N, 19.63; found (%): C, 65.87; H, 625; N, 19.18; melting point: 275 °C.

**General procedure for the synthesis of mono-triazole-bispidines (8a and 8b)**

Compound **5** (1 eq., 0.1 M) was dissolved in a 1:1 acetone-water mixture. Then, azide **6a** or **6g** (1.1 eq.), CuSO_4_ (0.02 eq.), and sodium ascorbate (0.2 eq.) were added in sequence. The reaction was performed under microwave irradiation for 1 h at 50 °C. Then, the mixture was dissolved in ethyl acetate and washed with 1 M aqueous ammonia solution to accomplish the complete removal of the copper catalyst. The organic phase was dried over anhydrous Na_2_SO_4_, filtered, and the liquid evaporated under vacuum to give the desired pure product.

**8a: 3-Benzyl-7-((1-benzyl-1*H*-1,2,3-triazol-4-yl)methyl)-1,5-diphenyl-3,7-diaza-bicyclo[3.3.1]nonan-9-one.** Yield 91%. White solid. ^1^H-NMR (400 MHz, CDCl_3_) δ (ppm) 8.21 (s, 1H, triazole-H), 7.41–7.08 (m, 20H, Ar-H), 5.63 (s, 2H, triazole-CH_2_-Bn), 3.89 (s, 2H, CH_2_-Bn), 3.71 (s, 2H, N-CH_2_-triazole), 3.55 (d, *J* = 10.7 Hz, 2H, CH_2_-eq), 3.45 (d, *J* = 10.6 Hz, 2H, CH_2_-eq), 3.06 (d, *J* = 10.6 Hz, 2H, CH_2_-ax), 2.97 (d, *J* = 10.6 Hz, 2H, CH_2_-ax). ^13^C-NMR (101 MHz, DMSO-d_6_) δ (ppm) 211.2 (1C, C=O), 143.8 (1C, triazole-*ipso*), 143.4 (1C, Ph-*ipso*), 138.6 (1C, Ph-*ipso*), 136.7 (1C, Ph-*ipso*), 129.3 (4C, Ar), 129.1 (2C, Ar), 128.7 (2C, Ar), 128.5 (2C, Ar), 128.1 (4C, Ar), 127.6 (2C, Ar), 127.2 (2C, Ar), 126.8 (2C, Ar), 124.6 (1C, CH-triazole), 64.5 (4C, CH_2_-bispidine), 61.1 (1C, N-CH_2_-triazole), 60.2 (1C, CH_2_ bispidine), 54.5 (1C, CH_2_-Bn), 53.2 (1C, CH_2_-triazole), 51.9 (2C, C-Ph bispidine). Elemental analysis: C_36_H_35_N_5_O: (553.71); calculated (%): C, 78.09; H, 6.37; N, 12.65; found (%): C, 78.97; H, 6.81; N, 12.99; melting point: 259 °C.

**8b: 3-Benzyl-1,5-diphenyl-7-((1-undecyl-1*H*-1,2,3-triazol-4-yl)methyl)-3,7-diaza-bicyclo[3.3.1]nonan-9-one.** Yield 94%. Yellow oil. ^1^H-NMR (400 MHz, DMSO-d_6_) δ (ppm) 8.13 (s, 1H, triazole-H), 7.42–7.11 (m, 15H, Ar-H), 4.36 (t, *J* = 6.9 Hz, 2H, alk-CH_2_-triazole), 3.88 (s, 2H, CH_2_-Bn), 3.72 (s, 2H, N-CH_2_-triazole), 3.54 (d, *J* = 10.8 Hz, 2H, CH_2_-eq), 3.46 (d, *J* = 10.8 Hz, 2H, CH_2_-eq), 3.05 (d, *J* = 10.8 Hz, 2H, CH_2_-ax), 2.99 (d, *J* = 10.8 Hz, 2H, CH_2_-ax), 1.82 (dd, *J* = 14.1, 7.0 Hz, 2H, alk), 1.17 (d, *J* = 14.3 Hz, 16H, alk), 0.84 (t, *J* = 6.8 Hz, 3H, alk). ^13^C-NMR (101 MHz, DMSO-d_6_) δ (ppm) 211.2 (1C, C=O), 143.4 (1C, triazole-*ipso*), 143.3 (1C, Ph-*ipso*), 138.6 (1C, Ph-*ipso*), 129.3 (4C, Ar), 128.7 (2C, Ar), 128.1 (2C, Ar), 127.6 (4C, Ar), 127.2 (2C, Ar), 126.7 (1C, Ar), 124.2 (1C, CH-triazole), 64.6 (2C, CH_2_-bispidine), 64.4 (2C, CH_2_-bispidine), 61.1 (1C, N-CH_2_-triazole), 54.5 (2C, CH_2_-Bn + CH_2_-triazole), 51.9 (1C, C-Ph bispidine), 49.7 (1C, alk), 31.7 (1C, alk), 30.2 (1C, alk), 29.3 (2C, alk), 29.1 (3C, alk), 28.8 (1C, alk), 26.2 (1C, alk), 22.5 (1C, alk), 14.4 (1C, alk). Elemental analysis: C_40_H_51_N_5_O: (617.88); calculated (%): C, 77.76; H, 8.32; N, 11.33; found (%): C, 78.26; H, 8.70; N, 11.67.

**General procedure for the synthesis of the bispidine-metal complexes**

The 1:1 bispidine–metal complexes were prepared by stirring at room temperature a 0.1 M solution of the ligand in acetonitrile with a stoichiometric amount of the salt for 24 h. The resulting precipitate was filtered off on a Buchner funnel and submitted to ESI-MS analysis and NMR in CD_3_CN.

**Henry reaction’s optimized procedure**

Aldehyde (1 eq, 0.2 M) was dissolved in methanol; 15% mol **7a·Zn** catalyst was added, and then nitromethane (5 eq) was slowly dripped into the mixture. The reaction was left stirring for 1 h at 80 °C under microwave irradiation; the crude was then filtered, and the liquid phase submitted to GC-MS analysis for the conversion quantification.

### 4.3. Crystallographic Data

Poor-quality crystals of **8a** were obtained as transparent plates by the slow evaporation of an acetone/ethanol 1:1 solution at room temperature. Diffraction data were collected on a Bruker-AXS CCD-based three circle diffractometer, working at 150 K with graphite-monochromatized Mo-Kα X-radiation (λ = 0.7107 Å), employing a 0.55 × 0.22 × 0.02 mm crystal. The structure was solved by direct method via SIR-2014 [30] and completed by iterative cycles of full-matrix least squares refinement on F_o_^2^ and ΔF synthesis using SHELXL-2019/3 [31] on the WinGX v2021.3 suite [32]. H atoms were generated stereochemically and refined by a riding model; U_iso_(H) was defined as 1.2 Ueq of the parent carbon atom for phenyl and methylene residues. The analyzed crystal was a two-component pseudo-merohedral twin in the monoclinic system, found using the TwinRotMat routine in PLATON [22]. Twin law [−1 0 0 0 −1 0 1 0 1] reduced the R1 residual [I > 2σ(I)] from 0.103 to 0.0629, and the mass ratio of the twin components was refined to 0.894(5):0.106.

Crystal data for **8a**. Formula: C_36_H_35_N_5_O_1_; MW = 553.69 g/mol; Bravais lattice: monoclinic; Space group: Cc (n° 9); *Z* = 4; Cell parameters: *a* = 15.354(2) Å, *b* = 15.453(2) Å, *c* = 51.948(10) Å, β = 98.65(3)°; V = 12185(4) Å^3^; D_calc_ =1.207 Mg/m^3^; T = 150(2)K; μ = (MoKα) 0.074 mm^−1^; 2θ_min_ = 1.189°; 2θ_max_ = 25.850°; Limiting indices = −17 ≤ h ≤ 17, −18 ≤ k ≤ 18, −63 ≤ l ≤ 61; Crystal size: 0.55 × 0.22 × 0.02 mm; F(000) = 4704; R_int_ = 0.0422; Data/restraints/parameters: 17072/5/1514; R = 0.0629 for 13880 reflections with I > 2σ(I) (R = 0.0885 for all 17072 unique/27192 collected reflections); wR2 = 0.1009 for reflections with I > 2σ(I) (wR2 = 0.1110 for all unique reflections); GOOF: 1.139; Residual positive and negative electron densities in the final map: 0.226 and −0.268 eÅ^−3^.

## Figures and Tables

**Table 1:** Library of the synthesized bis-triazole bispidines.

Compound	R	Yield (%)
**7a**	benzyl	95
**7b**	*p*-Cl-benzyl	75
**7c**	*p*-NO_2_-benzyl	78
**7d**	2-(azidomethyl)pyridine	84
**7e**	4-(azidomethyl)pyridine	83
**7f**	*n*-octyl	86
**7g**	*n*-undecyl	73
**7h**	3-hydroxypropyl	85
**7i**	-CH_2_PhCH_2_-	traces
**7j**	-CH_2_(CH_2_)_4_CH_2_-	traces

**Table 2:** Comparison of the free ligands **7a**,**b**,**d**,**h** and their metal complexes: list of the ∆δ in the ^1^H-NMR spectra.

Entry	Compound	∆δ for H_2,4,6,8_ eq (ppm)	∆δ for H_2,4,6,8_ ax (ppm)	∆δ for H_A_ (ppm)	∆δ for H_B_ (ppm)	∆δ for H_C_ (ppm)
1	**7b·Zn(II)**	0.46	0.44	0.18	0.50	0.15
2	**7d·Zn(II)**	0.50	0.61	0.27	0.24	0.15
3	**7d·La(III)**	0.45	0.70	0.43	0.23	0.03
4	**7a·La(III)**	0.46	0.71	0.42	0.24	0.04
5	**7h·La(III)**	0.45	0.70	0.40	0.40	0.05

**Table 3:** List of the ∆δ in the ^1^H-NMR spectrum for complex **8a·Pd(II)**.

∆δ for H_2,4,6,8_ eq (ppm)	∆δ for H_2,4,6,8_ ax (ppm)	∆δ for H_A-A_ (ppm)	∆δ for H_B_ (ppm)	∆δ for H_C_ (ppm)	∆δ for H_D_ (ppm)
0.82–0.84	0.80	0.66	0.28	0.18	0.68

**Table 4:** Screening of the Henry reaction using **7a·Zn** triazole bispidine as catalysts. Conditions: aldehyde (1 eq), nitromethane (5 eq), methanol (0.2 M), 1 h, 80 °C under microwave irradiation.

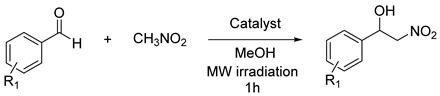			
Entry	Aldehyde	Catalyst Loading	Yield (%)
1 ^a^	*p*-NO_2_-PhCHO	Zn (2% mol)	7
2 ^a^	*p*-NO_2_-PhCHO	7a·Zn (2% mol)	79
3 ^a^	*p*-NO_2_-PhCHO	7a·Zn (15% mol)	99
4 ^a^	*p*-NO_2_-PhCHO	7a·Zn (15% mol) + TEA (2% mol)	81
5 ^a^	*p*-NO_2_-PhCHO	7a·Cu (2% mol)	22
6 ^a^	*p*-NO_2_-PhCHO	7a·Cu (15% mol)	56
7	*p*-Br-PhCHO	7a·Zn (15% mol)	92
8	*p*-F-PhCHO	7a·Zn (15% mol)	91
9	*p*-CF_3_-PhCHO	7a·Zn (15% mol)	94
10	*o*-NO_2_-PhCHO	7a·Zn (15% mol)	81
11	2-naphthaldehyde	7a·Zn (15% mol)	88

^a^ reactions were performed without the use of MW at RT for 24 h.

## Data Availability

CCDC-2063528 contains the supplementary crystallographic data for this paper. These data can be obtained free of charge via https://www.ccdc.cam.ac.uk/structures/ (accessed on 13 July 2023) or by e-mailing data_request@ccdc.cam.ac.uk, or by contacting The Cambridge Crystallographic Data Centre, 12 Union Road, Cambridge CB2 1EZ, UK; fax: +44(0)1223-336033.

## Supplementary Materials

The following are available online at https://www.mdpi.com/article/10.3390/molecules28176351/s1, Figures S1–S25: NMR data for the characterization of the synthesized products. Figures S26–S29: NMR titration spectra of bispidine-metal complexes. Figures S30–S33: Comparison of the experimental ESI-MS spectra of bispidine-metal complexes and the simulation of the isotopic pattern for the elemental composition. Table S1: Comparison of the free ligands **7a**,**b**,**d**,**h** and their metal complexes: list of the ∆δ in the ^1^H-NMR spectra. CCDC-2063528 contains the supplementary crystallographic data for this paper.

## Sample Availability

Not available.
