# Supplementary material for: Synthesis and Characterization of New Triazole-Bispidinone Scaffolds and Their Metal Complexes for Catalytic Applications
Source: Molecules. 2023 Aug 30;28(17):6351. doi: 10.3390/molecules28176351 (PMC10489160; doi:10.3390/molecules28176351)
Supplement: Supplementary file 1 [file molecules-28-06351-s001.zip › molecules-2532845-supplementary.pdf]

# Supplementary Materials

# Synthesis and characterization of new triazole-bispidinone scaffolds and their metal complexes for catalytic applications


Arianna Rossetti,[1,2,*] Alessandro Sacchetti,[1,2] Fiorella Meneghetti,[3,*] Greta Colombo Dugoni,[1] Matteo Mori,[3] Carlo Castellano[4]

1 Department of Chemistry, Materials and Chemical Engineering "G. Natta", Politecnico di Milano, via Mancinelli 7, 20131 Milano, Italy
2 INSTM - Local Unit c/o Politecnico di Milano, via Mancinelli 7, 20131 Milano, Italy
3 Department of Pharmaceutical Sciences, University of Milan, via L. Mangiagalli 25, 20133 Milano, Italy
4 Department of Chemistry, University of Milan, via C. Golgi 19, 20133 Milano, Italy
* Correspondence: arianna.rossetti@polimi.it; fiorella.meneghetti@unimi.it

# Table of Contents


1. **NMR DATA** **Pag. S3**

2. **NMR TITRATION OF BISPIDINE-METAL COMPLEXES** **Pag. S41**

3. **ESI-MS ANALYSES** **Pag. S44**

# 1. NMR DATA

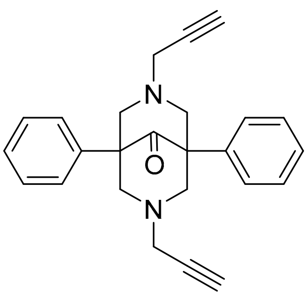
N
O
N

**1**

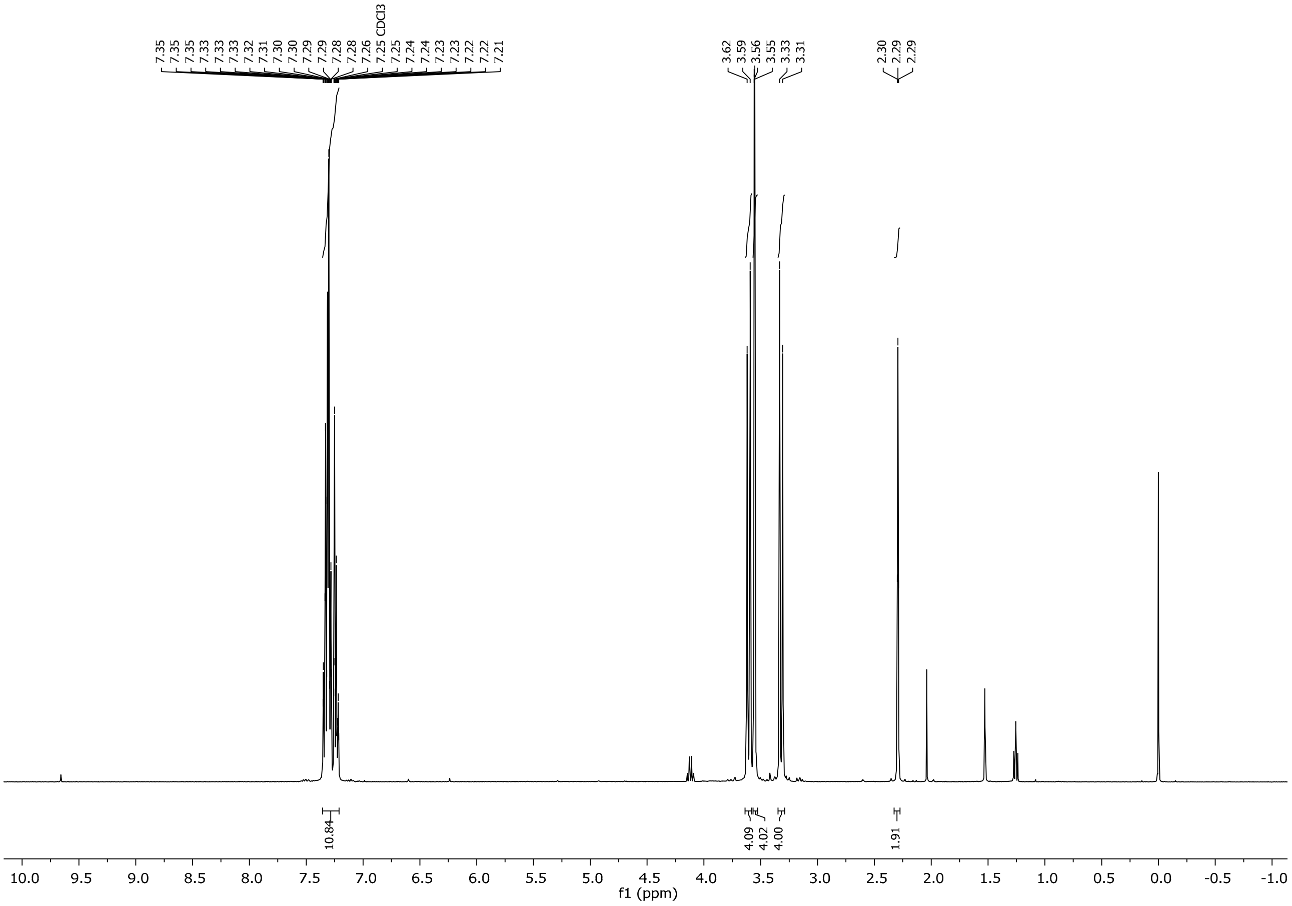
7.35
7.35
7.35
7.33
7.33
7.33
7.32
7.31
7.30
7.29
7.29
7.28
7.28
7.26
7.25 CDCl3
7.25
7.24
7.24
7.23
7.23
7.22
7.22
7.21
3.62
3.59
3.56
3.55
3.33
3.31
2.30
2.29
2.29
10.84
4.09
4.02
4.00
1.91
10.0 9.5 9.0 8.5 8.0 7.5 7.0 6.5 6.0 5.5 5.0 4.5 4.0 3.5 3.0 2.5 2.0 1.5 1.0 0.5 0.0 -0.5 -1.0
f1 (ppm)

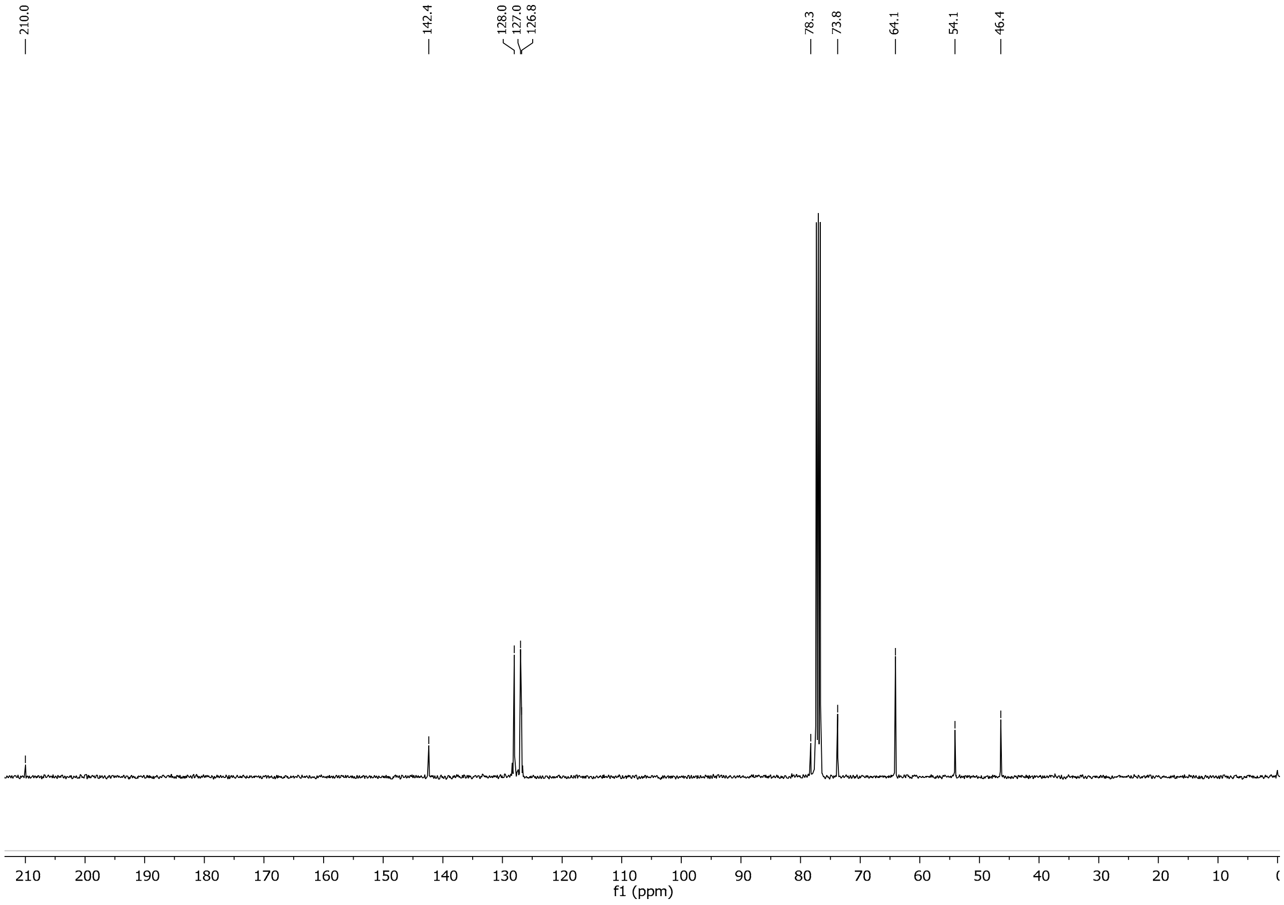


**Figure S1.** $^{1}$H and $^{13}$C-NMR spectra of compound **1**.

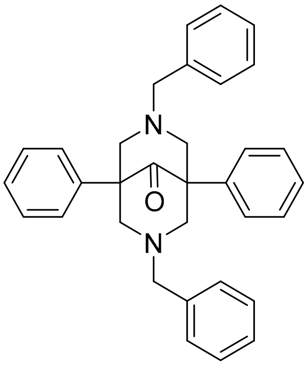


**2**

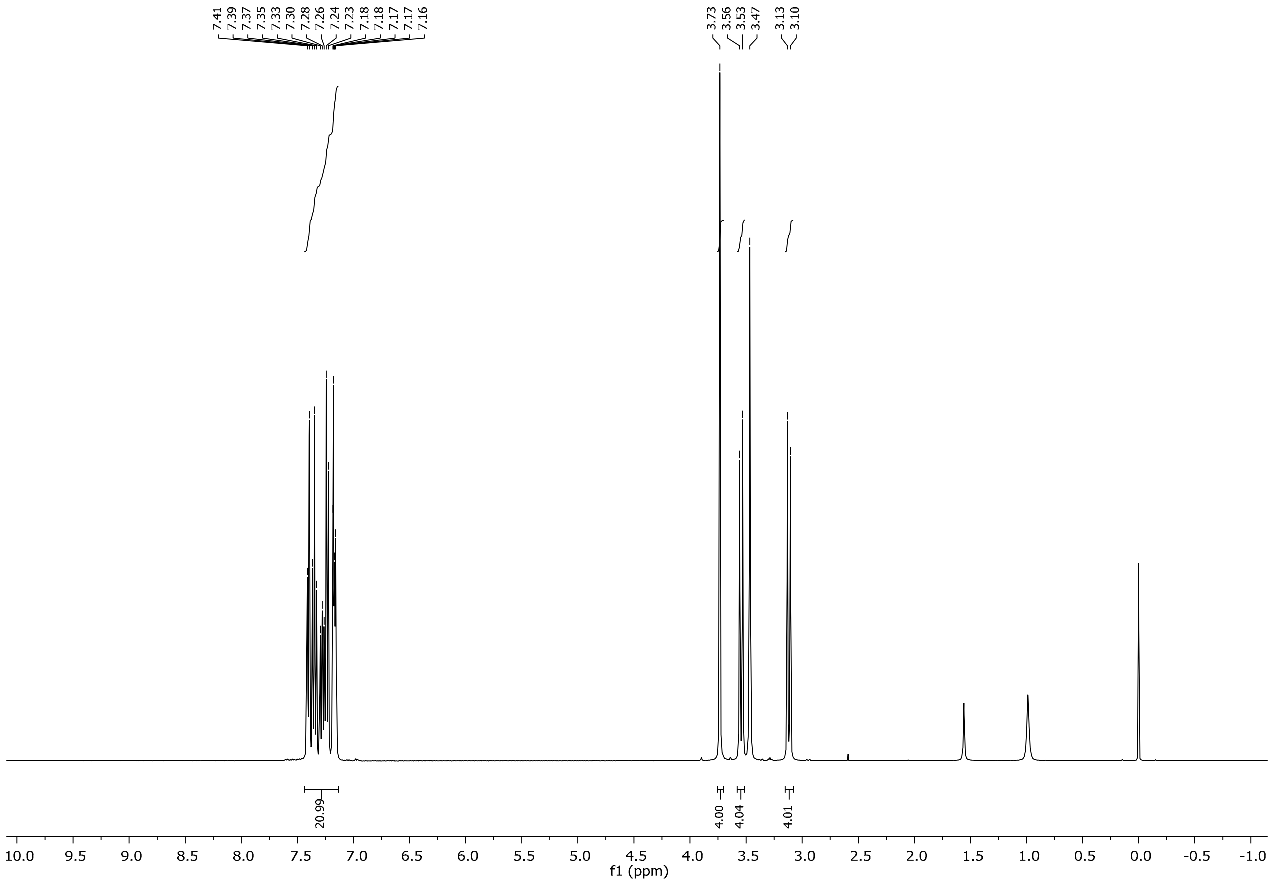


**Figure S2.** $^1$H-NMR spectrum of compound **2** in accordance with literature. [Black, D.S.C.; Deacon, G.B.; Rose, M. Synthesis and metal complexes of symmetrically N-substituted bispidinones. *Tetrahedron* **1995**, *51*, 2055–2076, doi:10.1016/0040-4020(94)01069-C.]

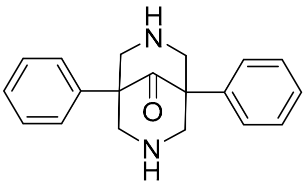


**3**

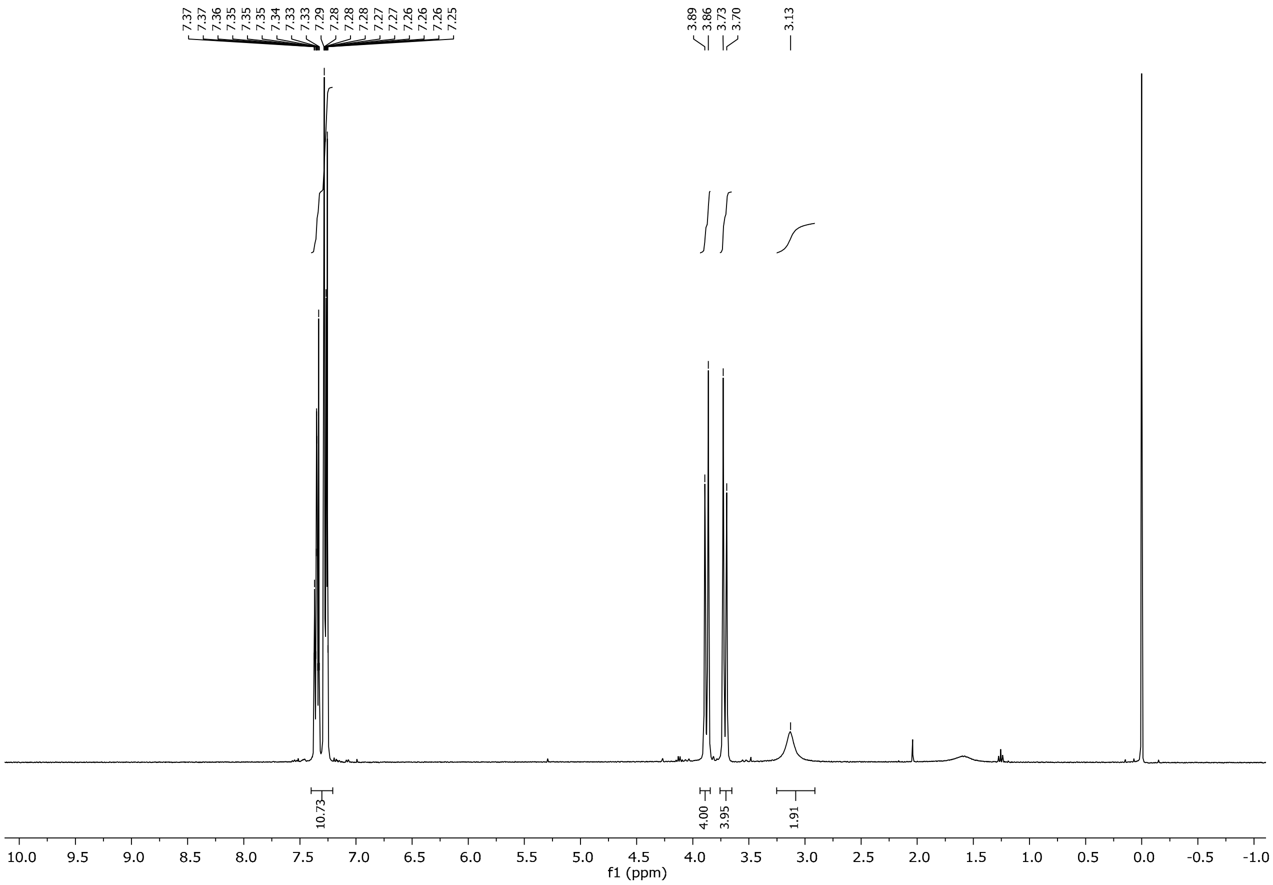


**Figure S3.** $^{1}H$-NMR spectrum of compound **3** in accordance with literature. [Black, D.S.C.; Deacon, G.B.; Rose, M. Synthesis and metal complexes of symmetrically N-substituted bispidinones. *Tetrahedron* **1995**, *51*, 2055–2076, doi:10.1016/0040-4020(94)01069-C.]

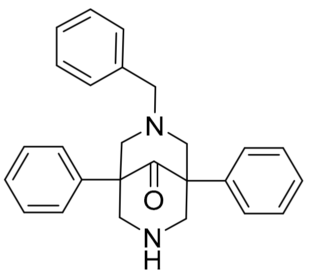
N
O
N
H

**4**

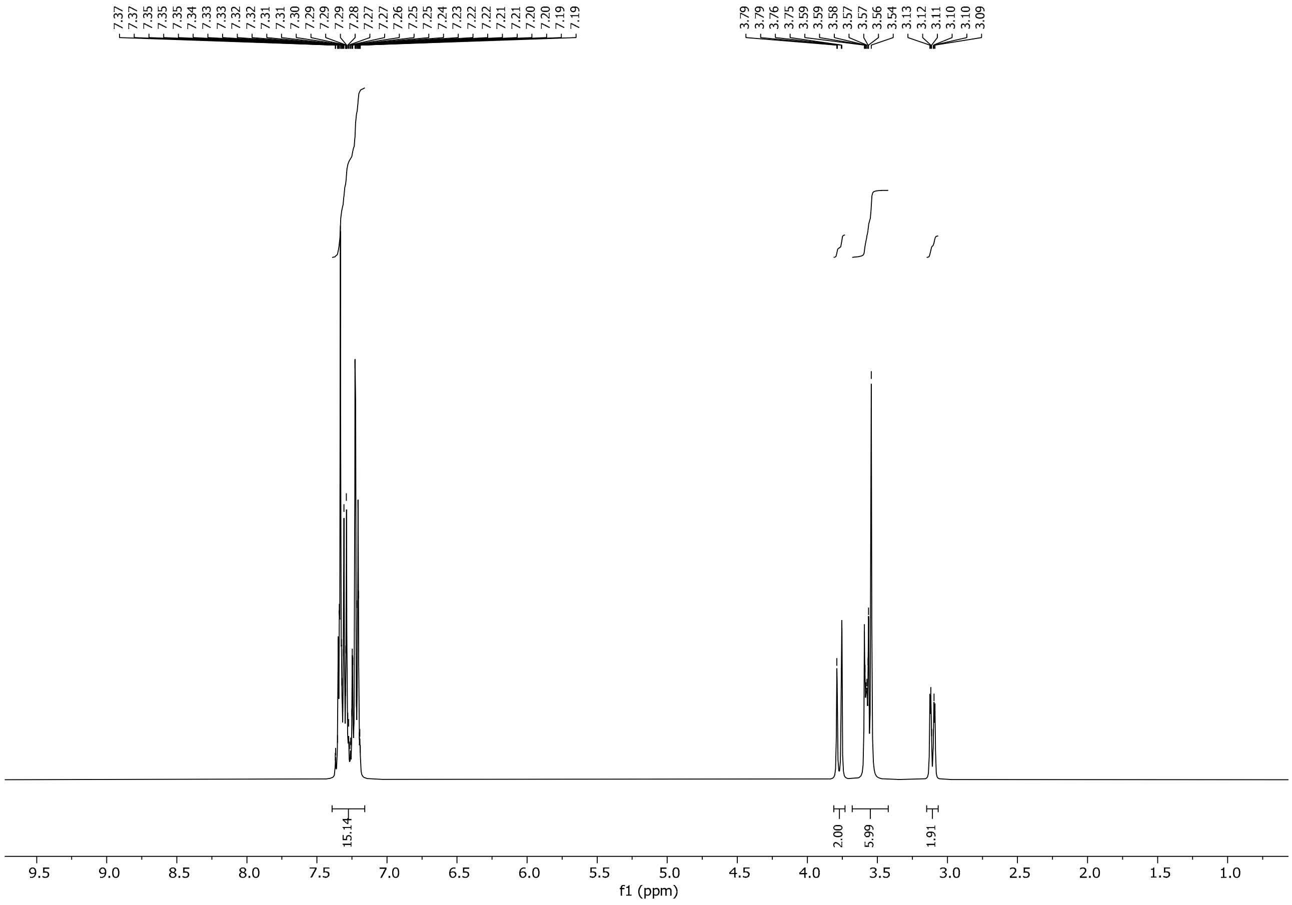
7.37 7.37 7.35 7.35 7.35 7.34 7.33 7.33 7.32 7.32 7.31 7.31 7.30 7.29 7.29 7.29 7.28 7.27 7.27 7.26 7.25 7.25 7.24 7.23 7.22 7.22 7.21 7.21 7.20 7.19 7.19
3.79 3.79 3.76 3.75 3.59 3.59 3.58 3.57 3.57 3.56 3.54 3.13 3.12 3.11 3.10 3.10 3.09
15.14
2.00
5.99
1.91
9.5 9.0 8.5 8.0 7.5 7.0 6.5 6.0 5.5 5.0 4.5 4.0 3.5 3.0 2.5 2.0 1.5 1.0
f1 (ppm)

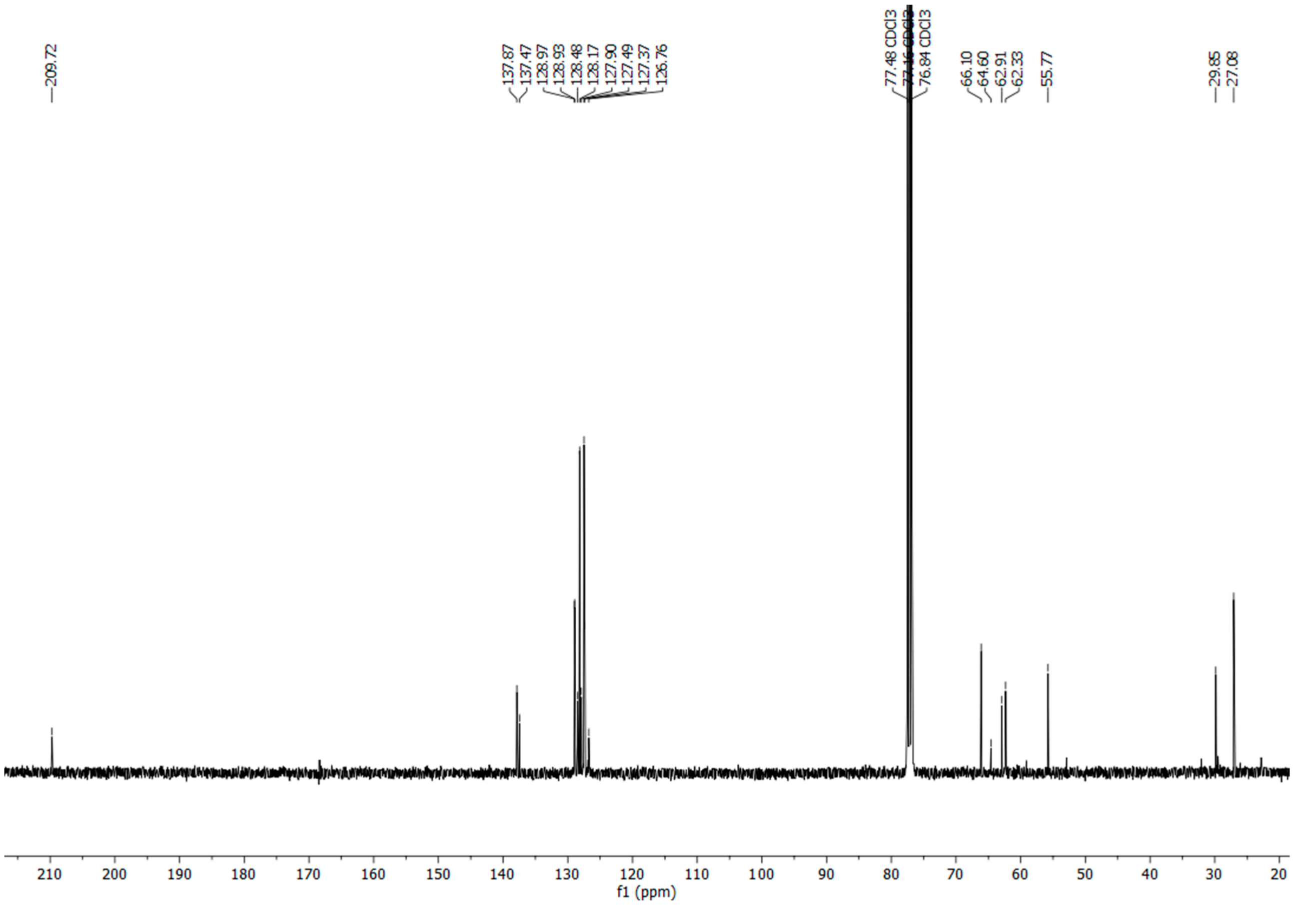


**Figure S4.** $^1$H and $^{13}$C-NMR spectra of compound **4**.

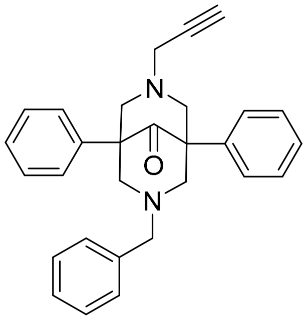

**5**

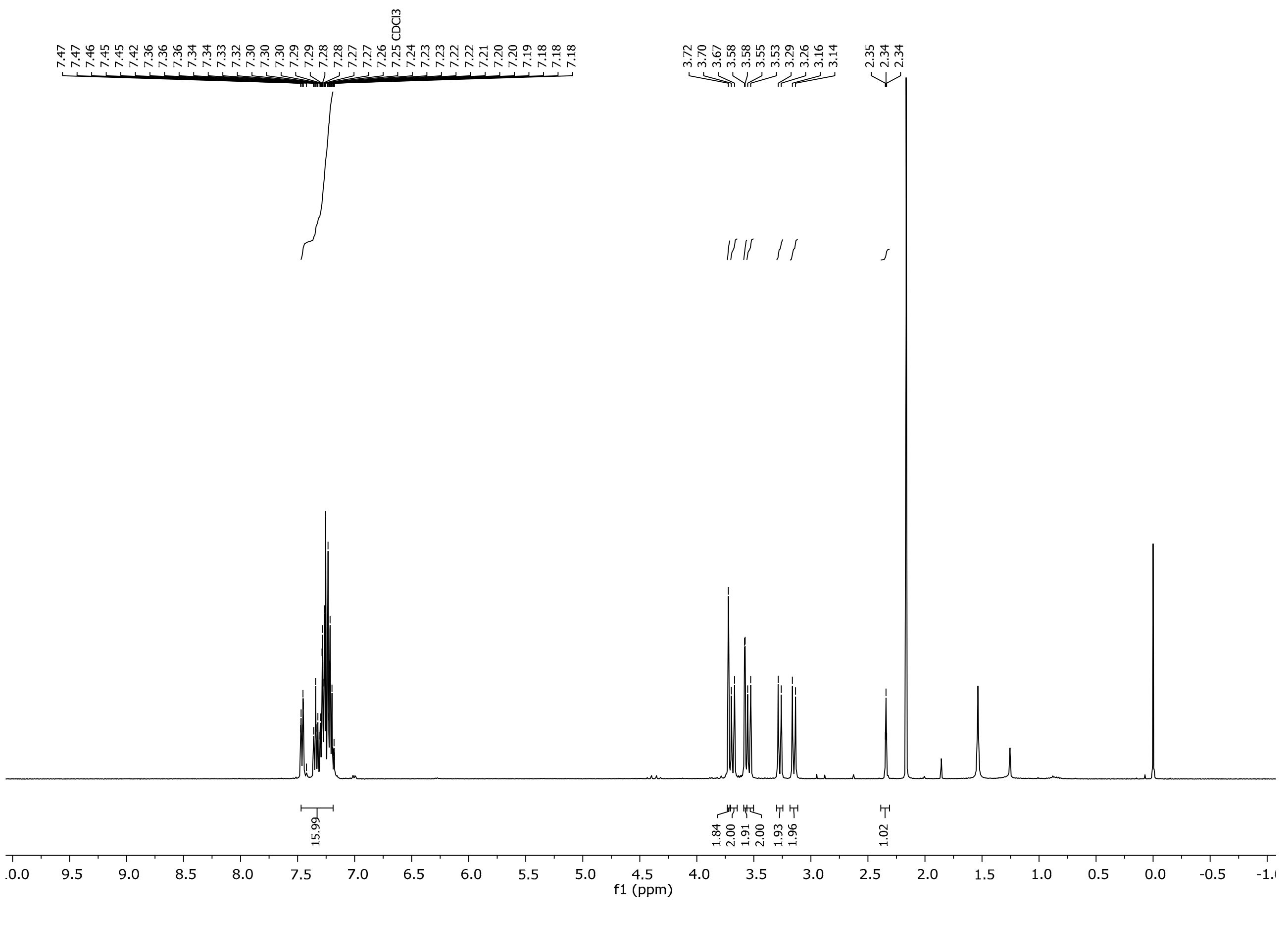
7.47 7.47 7.46 7.45 7.45 7.42 7.36 7.36 7.36 7.34 7.34 7.33 7.32 7.30 7.30 7.30 7.29 7.29 7.28 7.28 7.27 7.27 7.26 7.25 CDCl3 7.24 7.23 7.23 7.22 7.22 7.21 7.20 7.20 7.19 7.18 7.18 7.18
3.72 3.70 3.67 3.58 3.58 3.55 3.53 3.29 3.26 3.16 3.14
2.35 2.34 2.34
15.99
1.84 2.00 1.91 2.00 1.93 1.96
1.02
f1 (ppm)

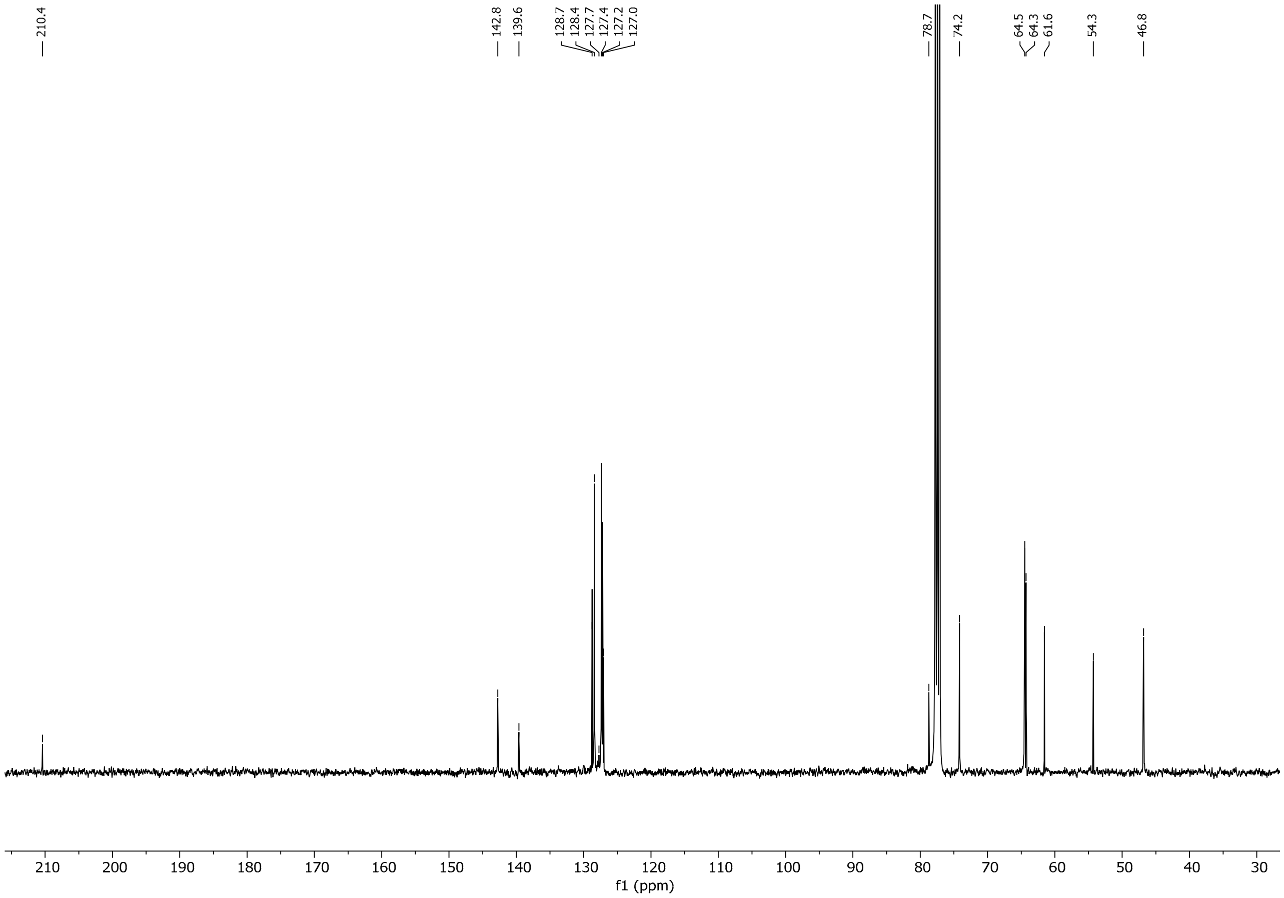


**Figure S5.** $^{1}$H and $^{13}$C-NMR spectra of compound **5**.

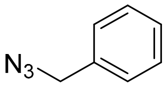


**6a**

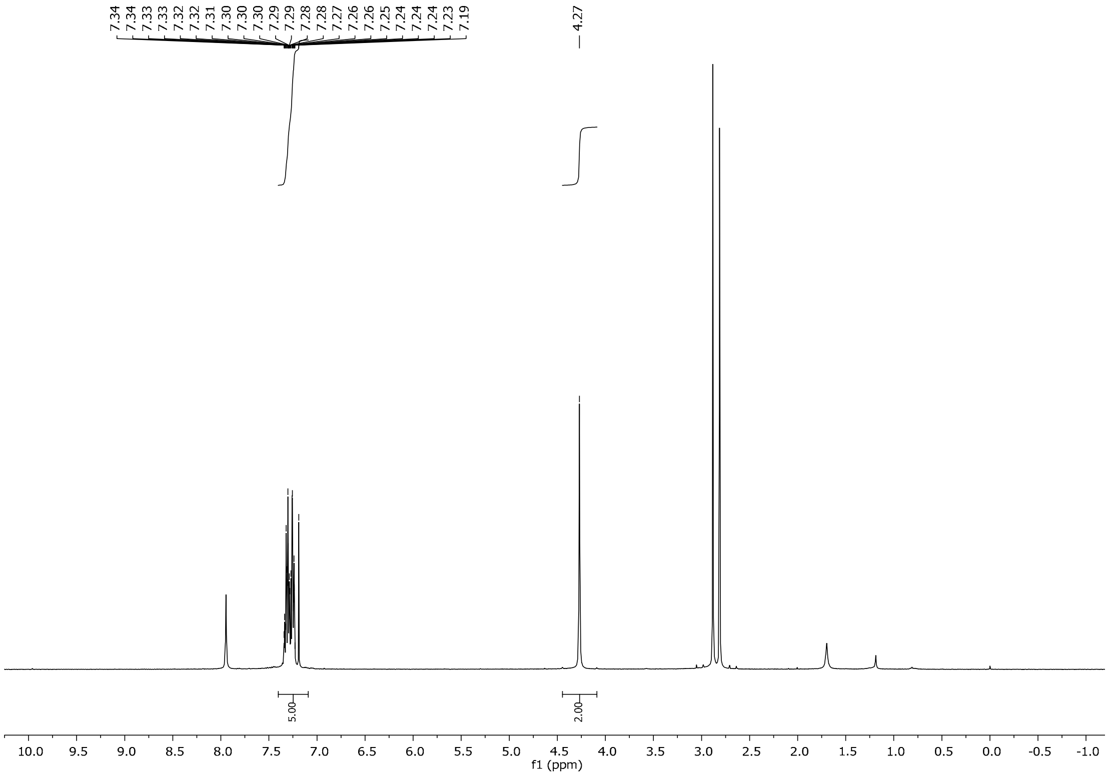


**Figure S6.** $^{1}$H-NMR spectrum of compound **6a** in accordance with literature. [Zhong, Z.; Chesti, J.; Armstrong, A.; Bull, J.A. Synthesis of Sulfoximine Propargyl Carbamates under Improved Conditions for Rhodium Catalyzed Carbamate Transfer to Sulfoxides *J. Org. Chem.* **2022** *87*, 16115-16126; doi: 10.1021/acs.joc.2c02083В.]

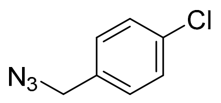


**6b**

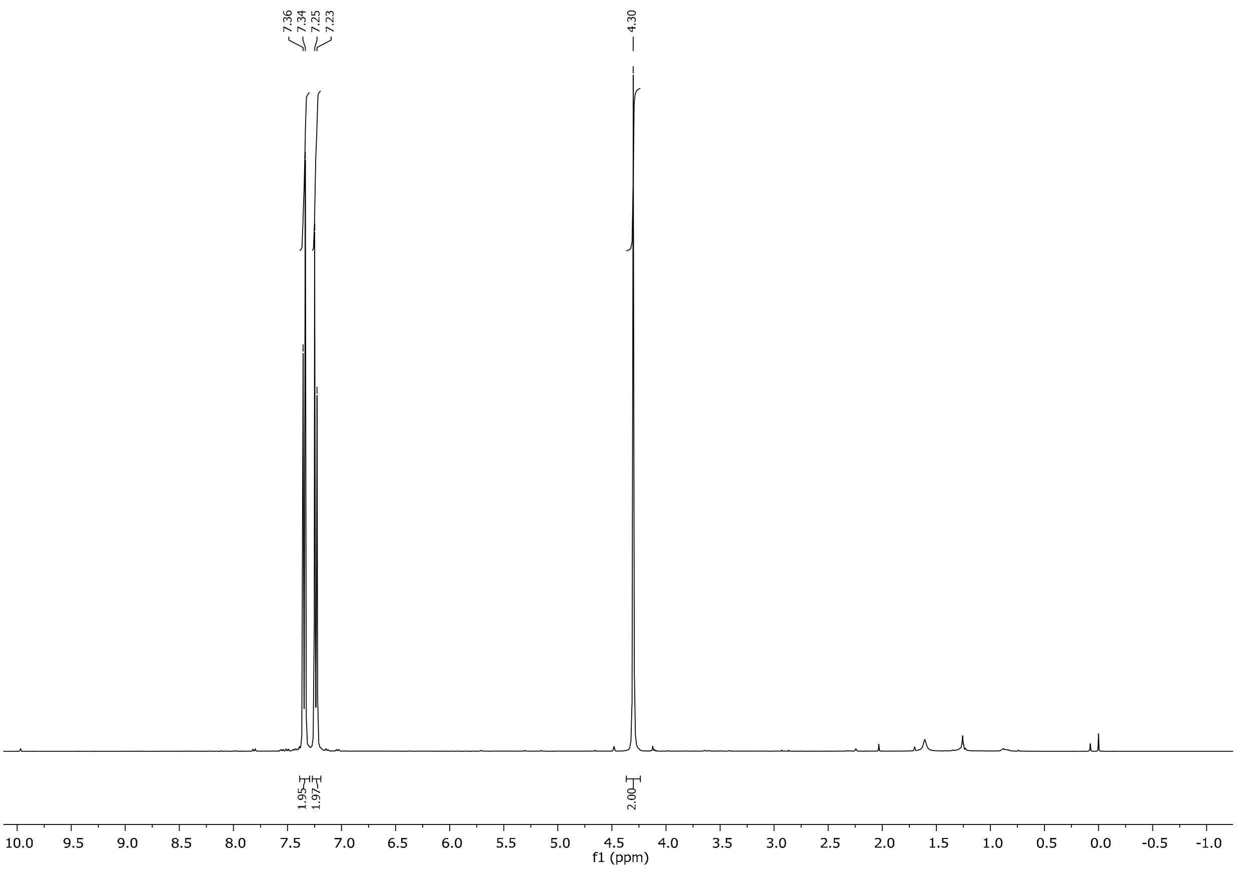


**Figure S7.** $^1$H-NMR spectrum of compound **6b** in accordance with literature. [Vairoletti, F.; Paulino, M.; Mahler, G.; Salinas, G.; Saiz, C. Structure-Based Bioisosterism Design, Synthesis, Biological Evaluation and In Silico Studies of Benzamide Analogs as Potential Anthelmintics. *Molecules* **2022**, *27*, 2659; doi: 10.3390/molecules27092659.]

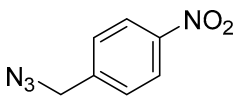


**6c**

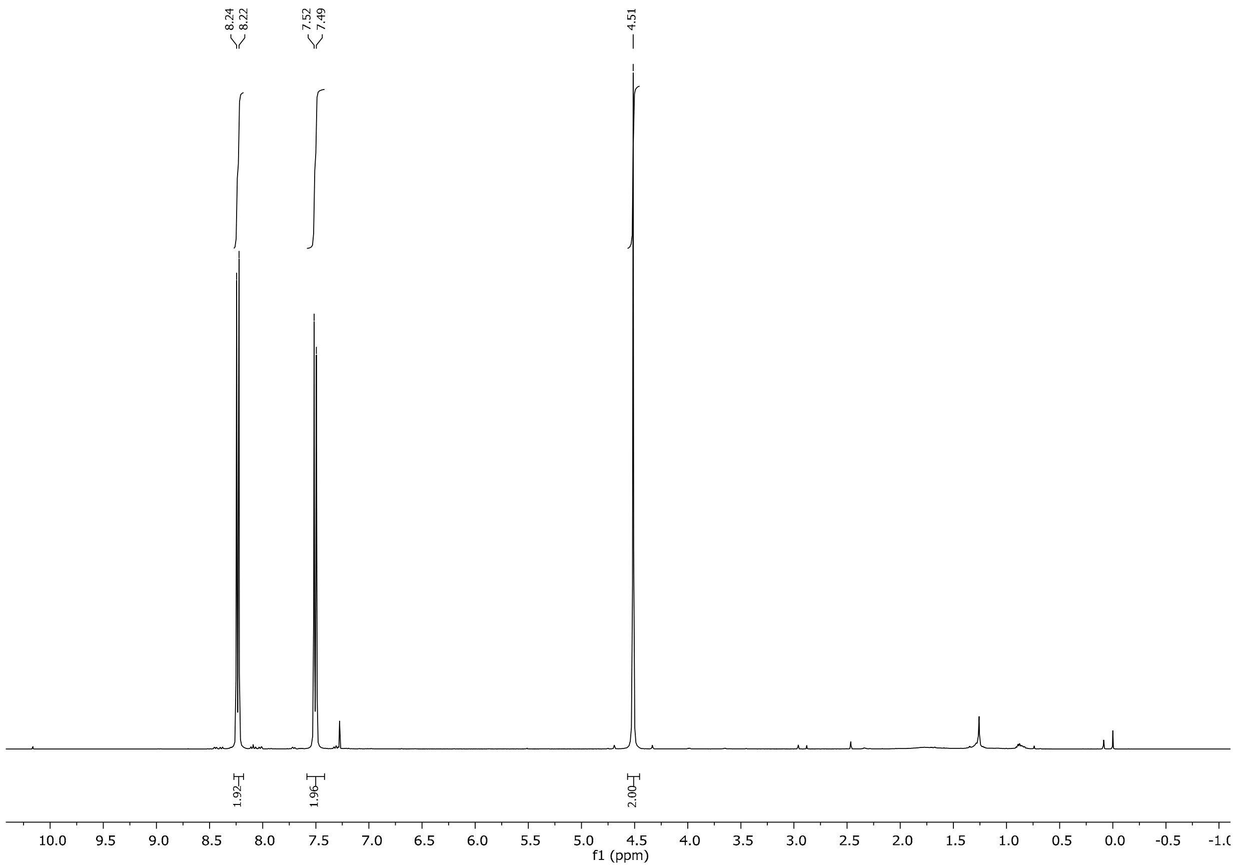


**Figure S8.** $^1$H-NMR spectrum of compound **6c** in accordance with literature. [Rabet, P.T.G.; Fumagalli, G.; Boyd, S.; Greaney, M.F. Benzylic C–H Azidation Using the Zhdankin Reagent and a Copper Photoredox Catalyst, *Org. Lett*. **2016** *18*, 1646-1649; doi: 10.1021/acs.orglett.6b00512.]

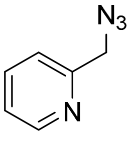


**6d**

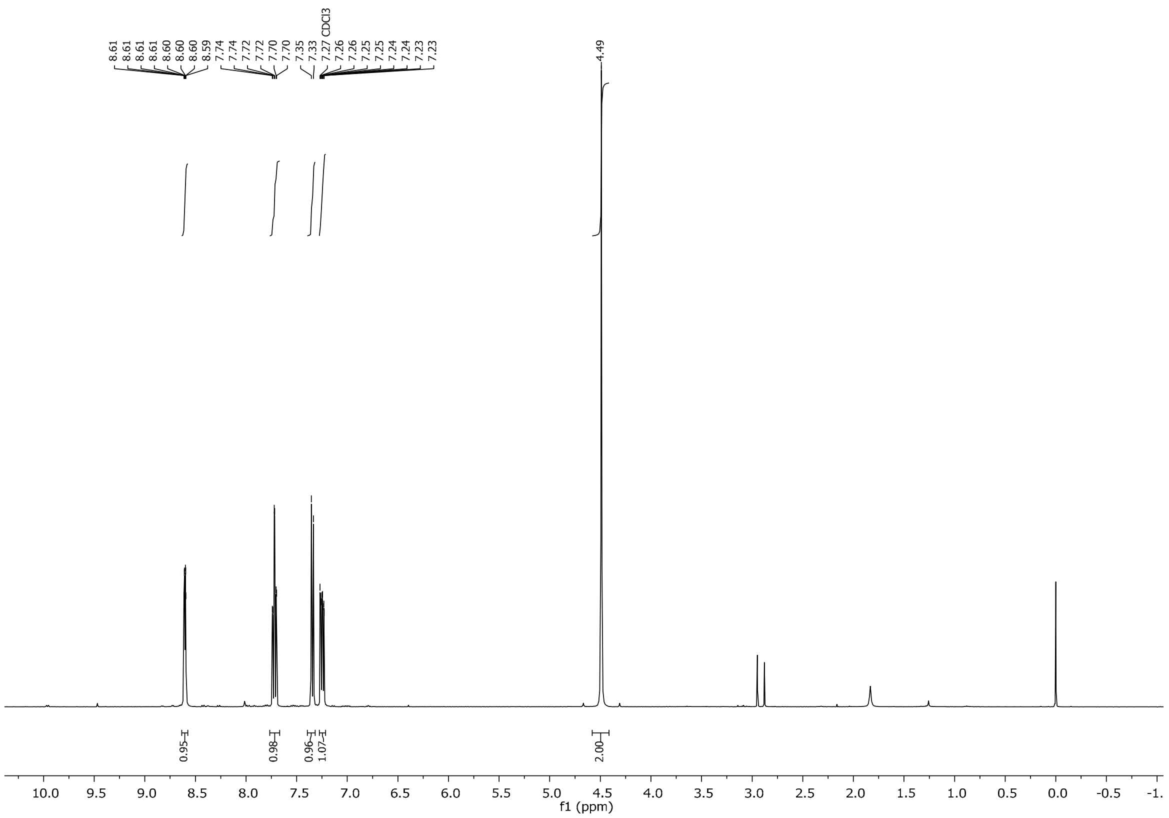


**Figure S9.** $^{1}$H-NMR spectrum of compound **6d** in accordance with literature. [Sacchetti, A.; Urra-Mancilla, C.; Colombo Dugoni, G. Synthesis of DPA-triazole structures and their application as ligand for metal catalyzed organic reactions, *Tetrahedron*, **2022**, 132581; doi: 10.1016/j.tet.2021.132581.]

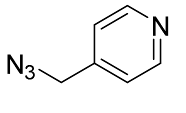


**6e**

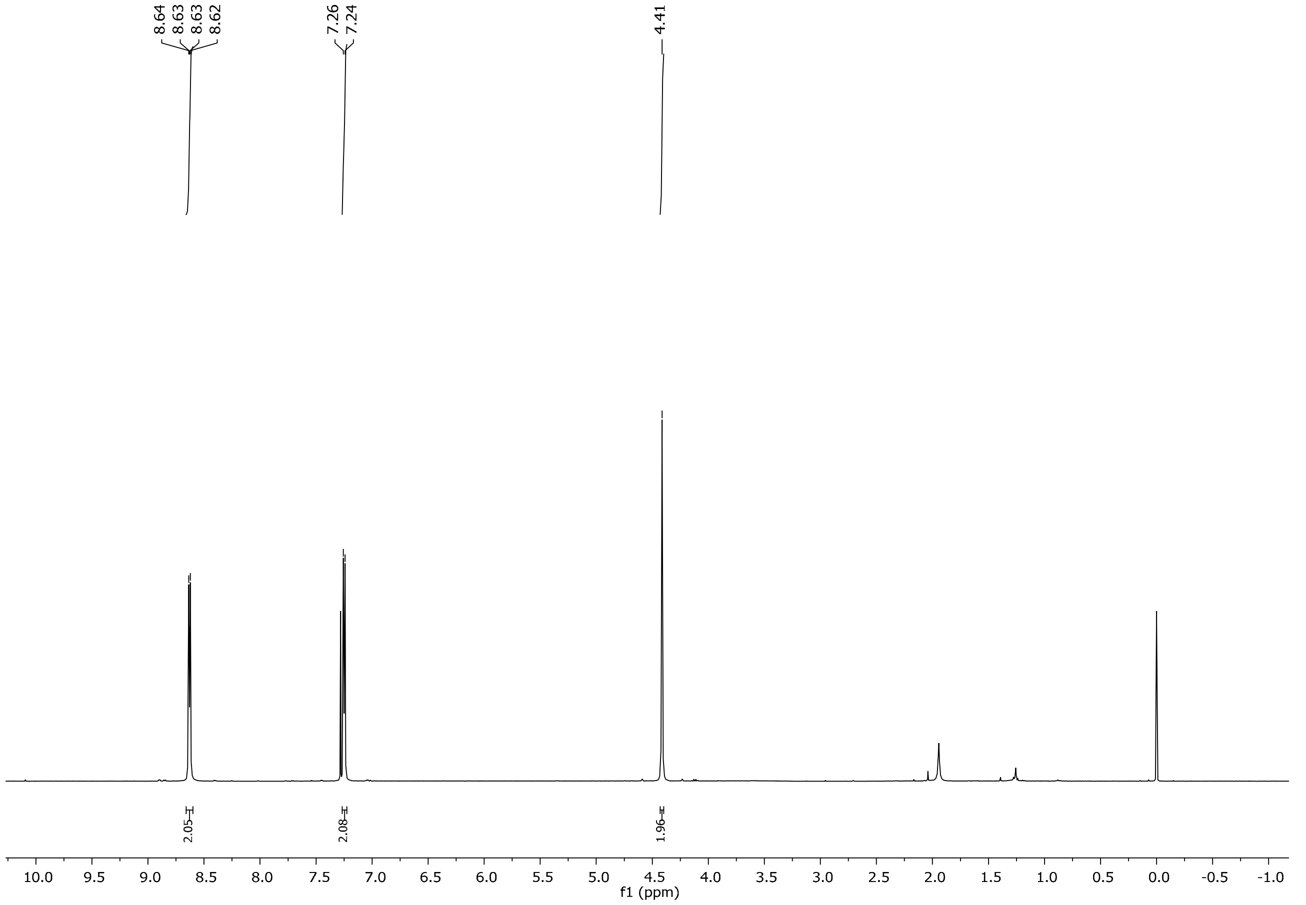


**Figure S10.** $^1$H-NMR spectrum of compound **6e** in accordance with literature. [Sacchetti, A.; Urra-Mancilla, C.; Colombo Dugoni, G. Synthesis of DPA-triazole structures and their application as ligand for metal catalyzed organic reactions, *Tetrahedron*, **2022**, 132581; doi: 10.1016/j.tet.2021.132581.]

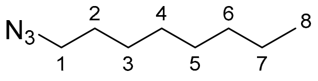


**6f**

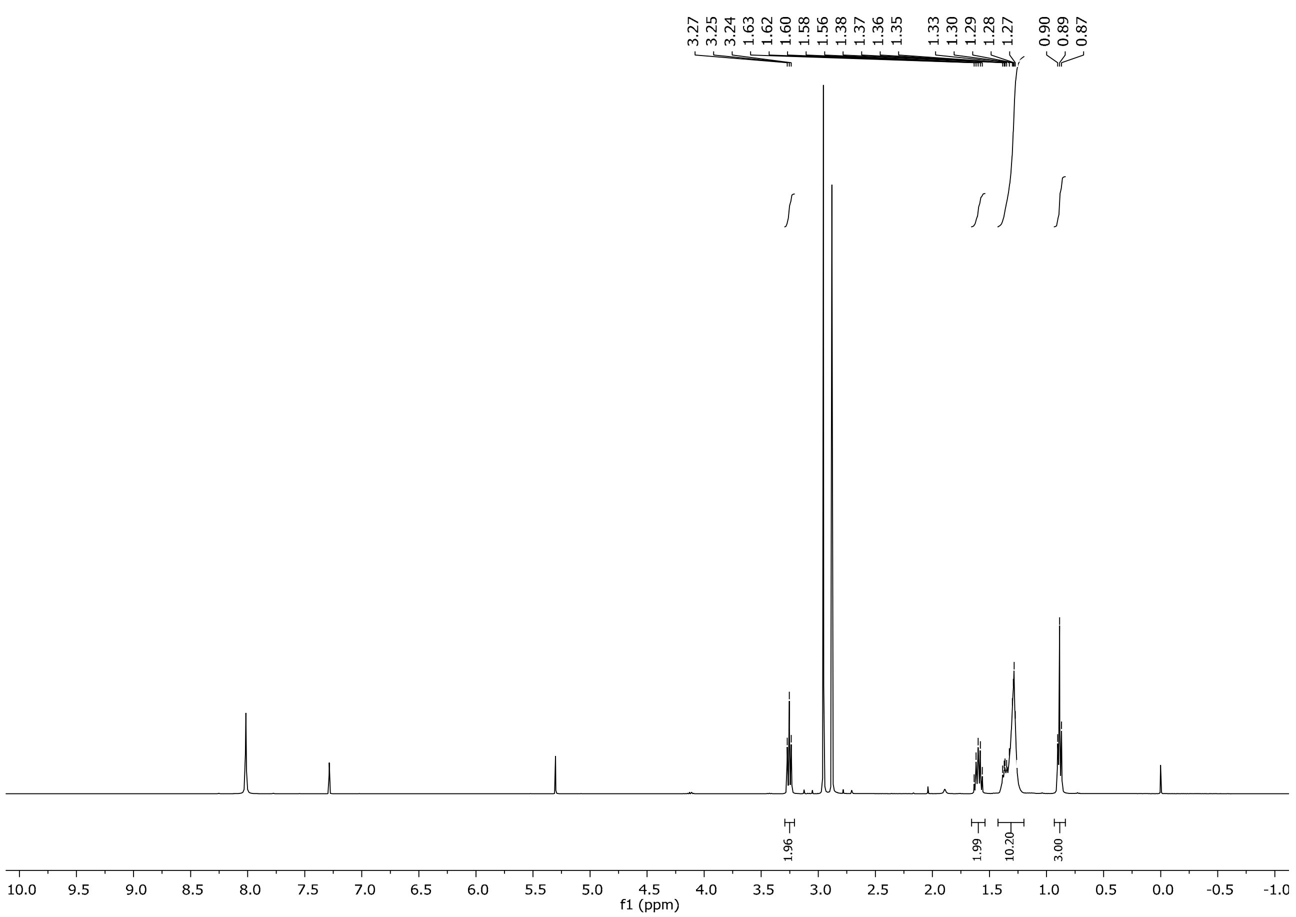


**Figure S11.** $^1$H-NMR spectrum of compound **6f** in accordance with literature. [Proietti, G.; Prathap, K.J.; Ye, X.; Olsson, R.T.; Dinér, P. Nickel Boride Catalyzed Reductions of Nitro Compounds and Azides: Nanocellulose-Supported Catalysts in Tandem Reactions. *Synthesis* **2022**; *54*, 133-146; doi: 10.1055/a-1579-2190.]

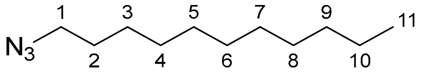


**6g**

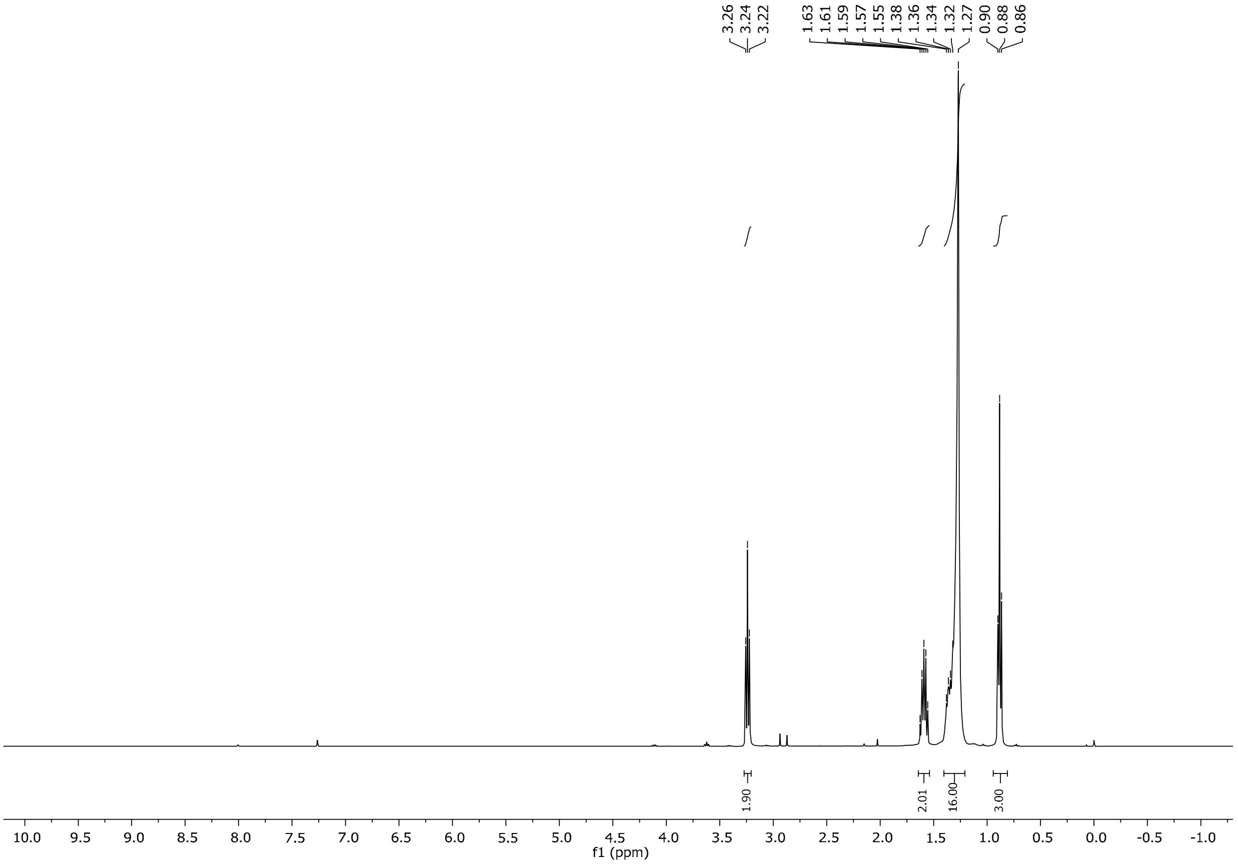


**Figure S12.** $^1$H-NMR spectrum of compound **6g** in accordance with literature. [Natarajan, B.; Jayaraman, N. Synthesis and studies of Rh(I) catalysts within and across poly(alkyl aryl ether) dendrimers, *J. Organometallic Chem.*, 696, **2011**, 722-730; doi: 10.1016/j.jorganchem.2010.09.054.]

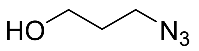


**6h**

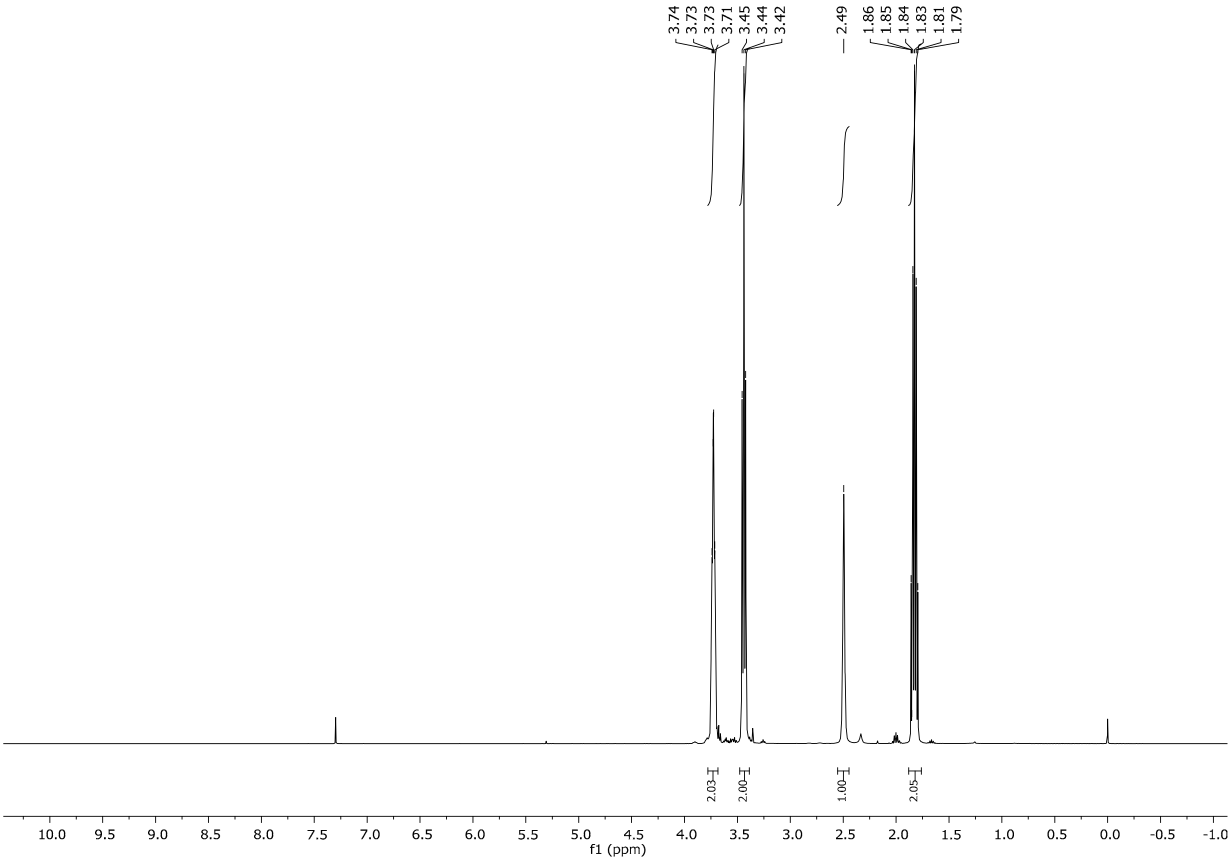


**Figure S13.** $^1$H-NMR spectrum of compound **6h** in accordance with literature. [Macerata, E.; Mossini, E.; Scaravaggi, S.; Mariani, M.; Mele, A.; Panzeri, W.; Boubals, N.; Berthon, L.; Charbonnel, M-C.; Sansone, F.; Arduini, A.; Casnati, A. Hydrophilic Clicked 2,6-Bis-triazolyl-pyridines Endowed with High Actinide Selectivity and Radiochemical Stability: Toward a Closed Nuclear Fuel Cycle *J. Am. Chem. Soc.* **2016** *138* (23), 7232-7235; doi: 10.1021/jacs.6b03106.]

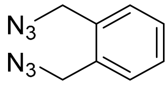


**6i**

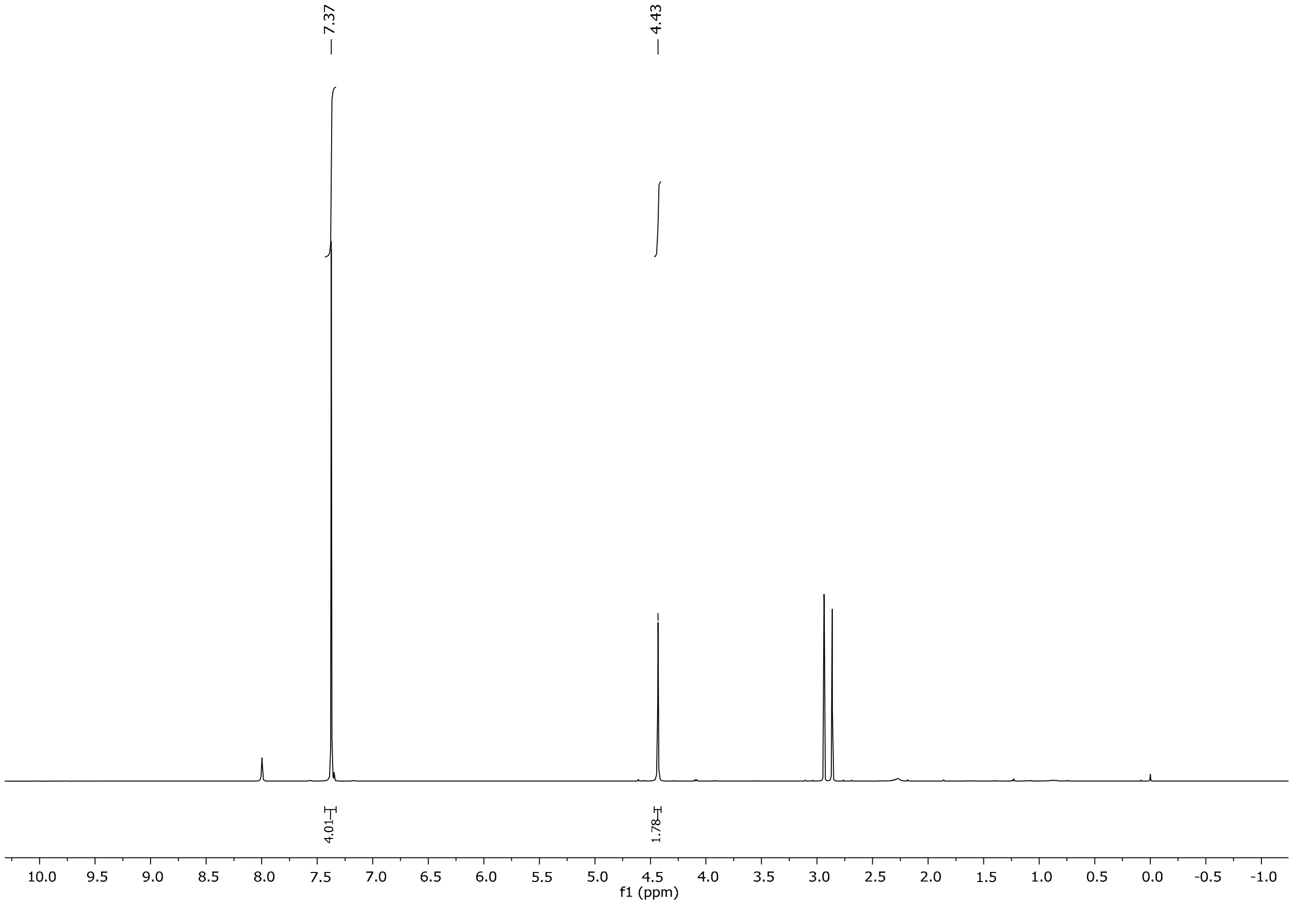


**Figure S14.** $^{1}H$-NMR spectrum of compound **6i** in accordance with literature. [Guo, Z.-F.; Yan, H.; Li, Z.-F.; Lu, Z.-L. Synthesis of mono- and di-[12]aneN3 ligands and study on the catalytic cleavage of RNA model 2-hydroxypropyl-p-nitrophenyl phosphate with their metal complexes, *Org. Biomol. Chem.*, **2011**, *9*, 6788-6796; doi: 10.1039/C1OB05942D.]

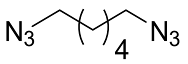


**6j**

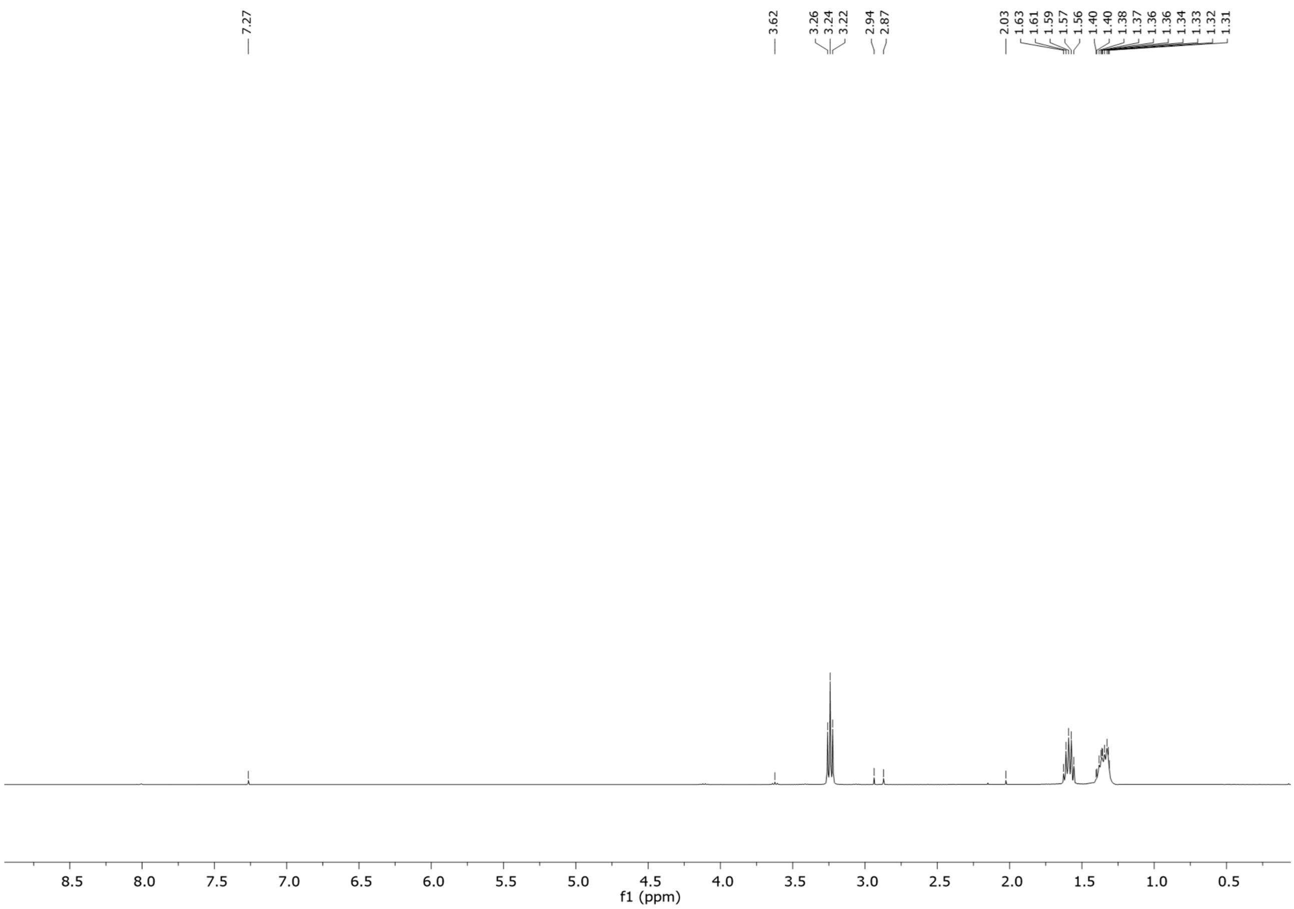


**Figure S15.** $^1$H-NMR spectrum of compound **6j** in accordance with literature. [Peng, L.; Zhao, Y.; Okuda, Y.; Le, L.; Tang, Z.; Yin, S.-F.; Qiu, R.; Orita, A. Process-Divergent Syntheses of 4- and 5-Sulfur-Functionalized 1,2,3-Triazoles via Copper-Catalyzed Azide–Alkyne Cycloadditions of 1-Phosphinyl-2-sulfanylethynes *The Journal of Organic Chemistry* **2023** *88* (5), 3089-3108; doi: 10.1021/acs.joc.2c02876.]

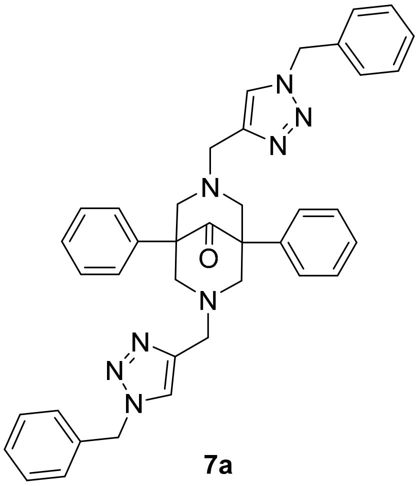

**7a**

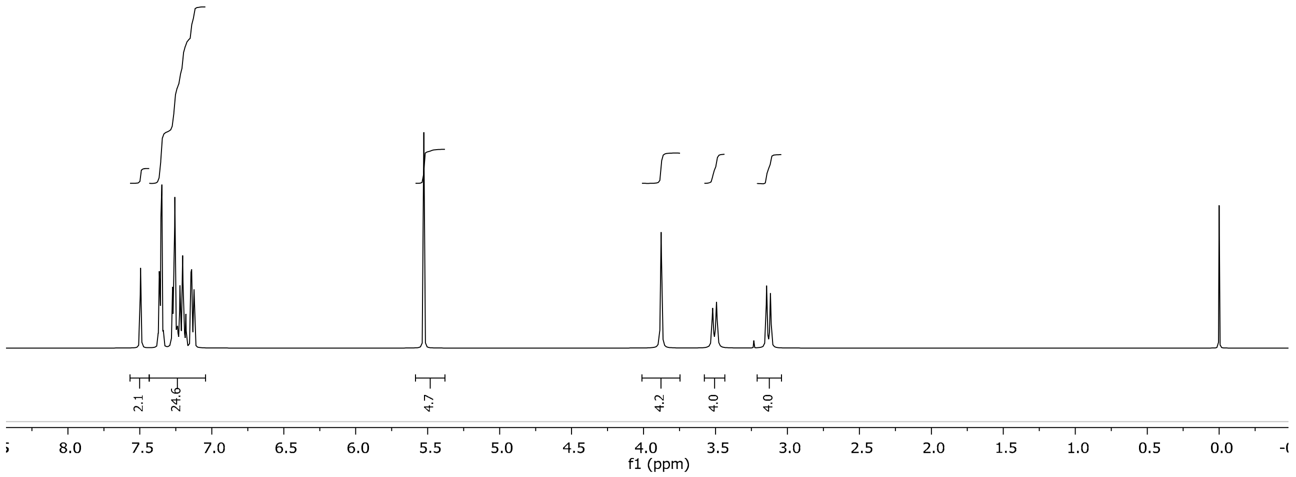

2.1
24.6
4.7
4.2
4.0
4.0
8.0
7.5
7.0
6.5
6.0
5.5
5.0
4.5
4.0
3.5
3.0
2.5
2.0
1.5
1.0
0.5
0.0
f1 (ppm)

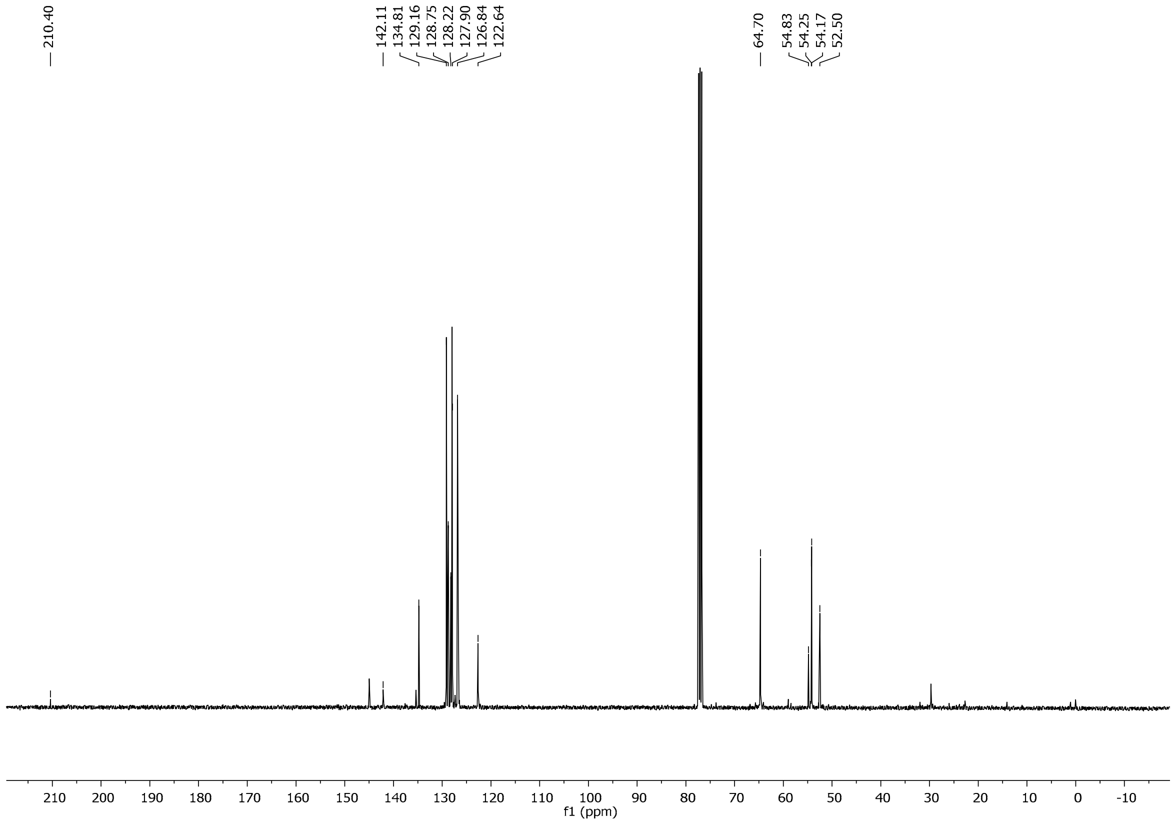


**Figure S16.** $^{1}$H and $^{13}$C-NMR spectra of compound **7a**.

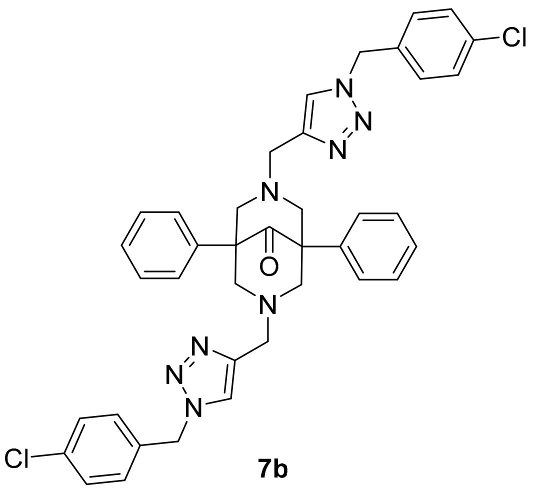

**7b**

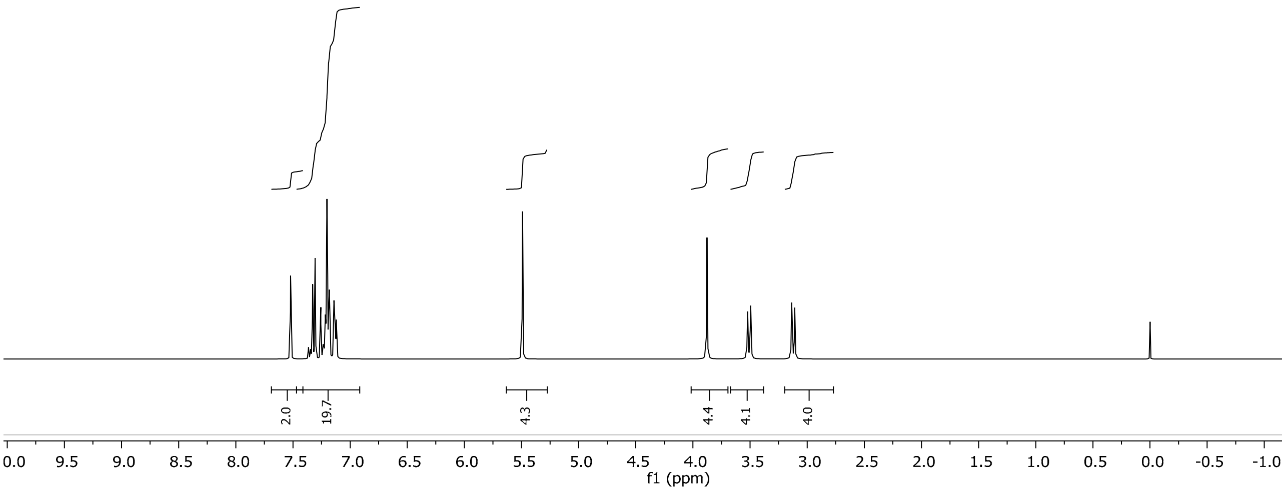
2.0
19.7
4.3
4.4
4.1
4.0
0.0 9.5 9.0 8.5 8.0 7.5 7.0 6.5 6.0 5.5 5.0 4.5 4.0 3.5 3.0 2.5 2.0 1.5 1.0 0.5 0.0 -0.5 -1.0
f1 (ppm)

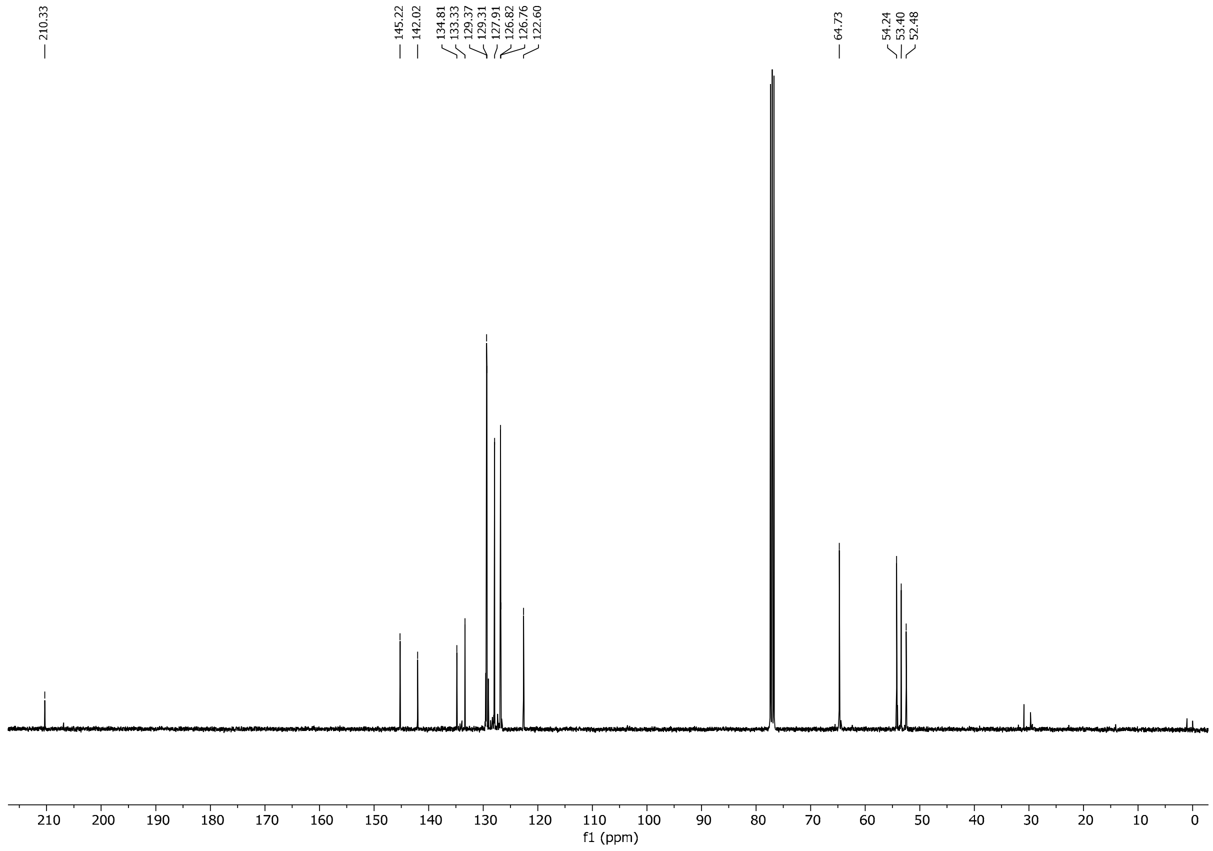


**Figure S17.** $^{1}$H and $^{13}$C-NMR spectra of compound **7b**.

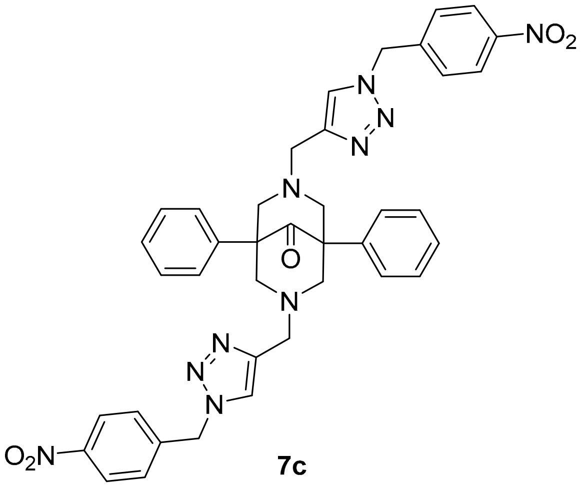
NO2
N
N
N
N
O
N
N
N
N
O2N

7c

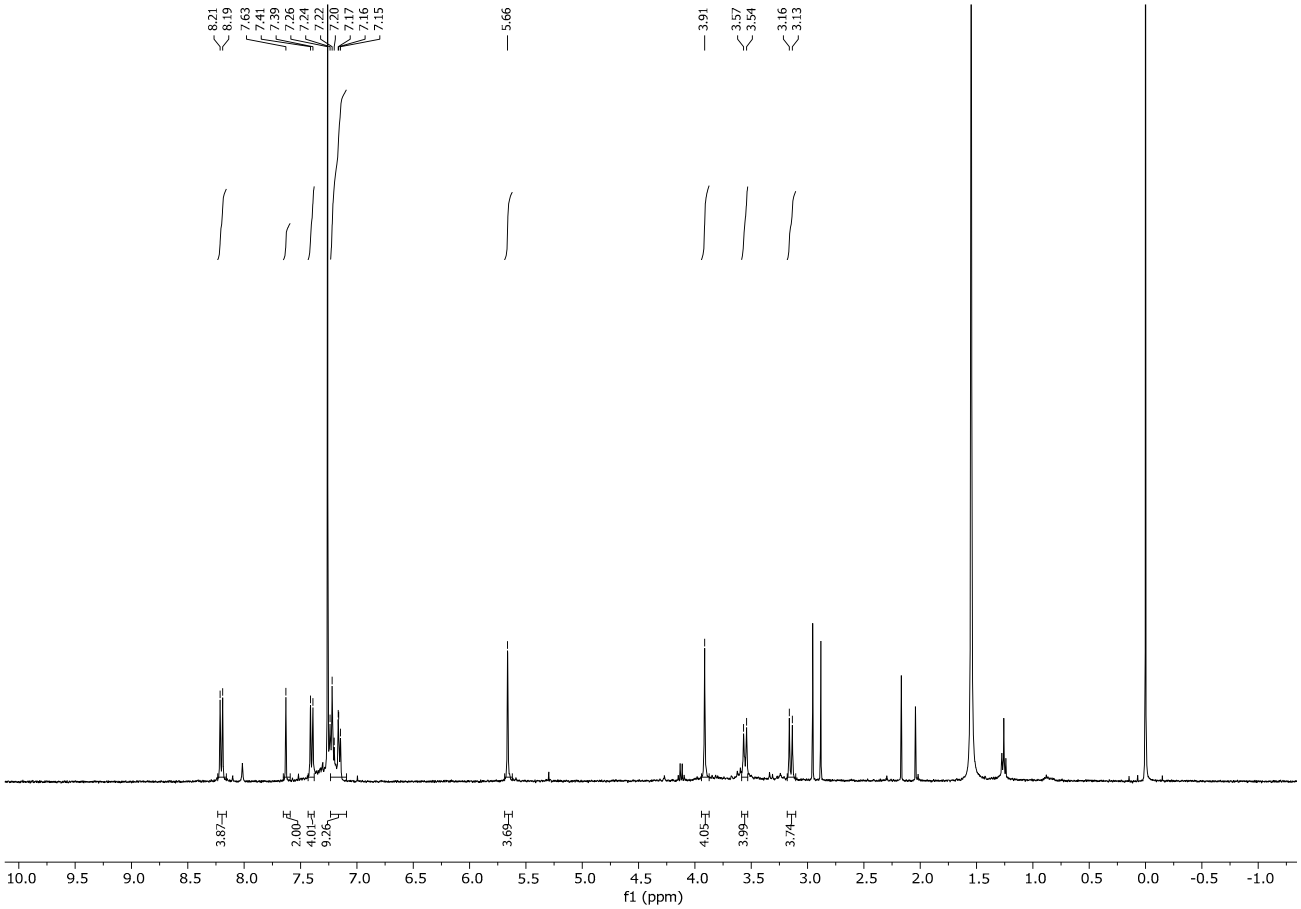
8.21
8.19
7.63
7.41
7.39
7.26
7.24
7.22
7.20
7.17
7.16
7.15
5.66
3.91
3.57
3.54
3.16
3.13
3.87
2.00
4.01
9.26
3.69
4.05
3.99
3.74
10.0 9.5 9.0 8.5 8.0 7.5 7.0 6.5 6.0 5.5 5.0 4.5 4.0 3.5 3.0 2.5 2.0 1.5 1.0 0.5 0.0 -0.5 -1.0
f1 (ppm)

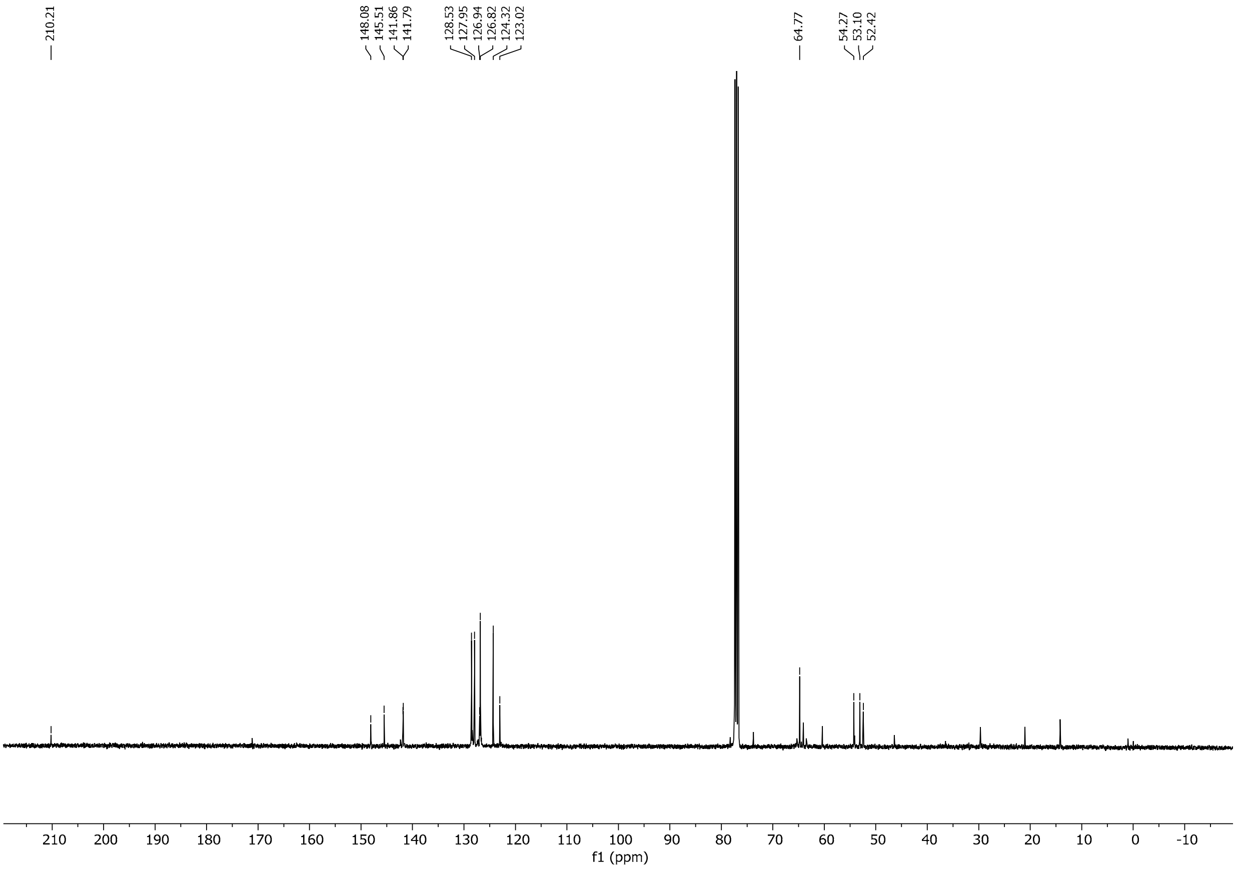


**Figure S18.** $^{1}$H and $^{13}$C-NMR spectra of compound **7c**.

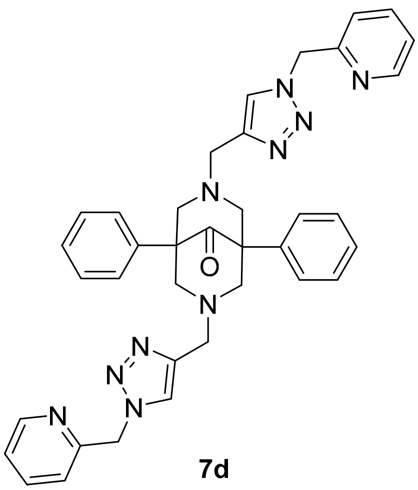
N
N
N
N
O
N
N
N
N
N
N

**7d**

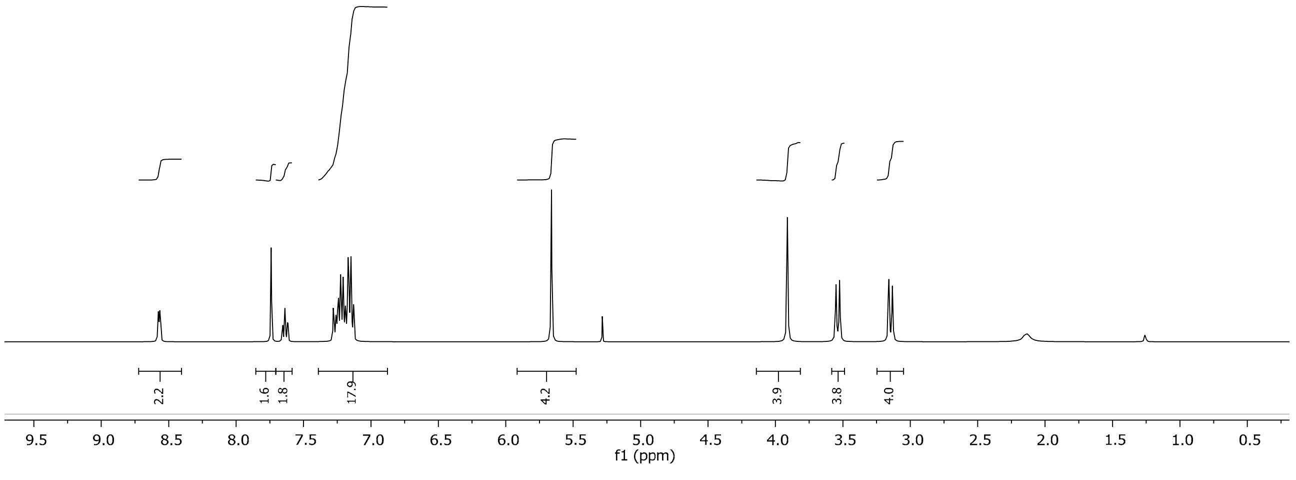
2.2
1.6
1.8
17.9
4.2
3.9
3.8
4.0
9.5
9.0
8.5
8.0
7.5
7.0
6.5
6.0
5.5
5.0
4.5
4.0
3.5
3.0
2.5
2.0
1.5
1.0
0.5
f1 (ppm)

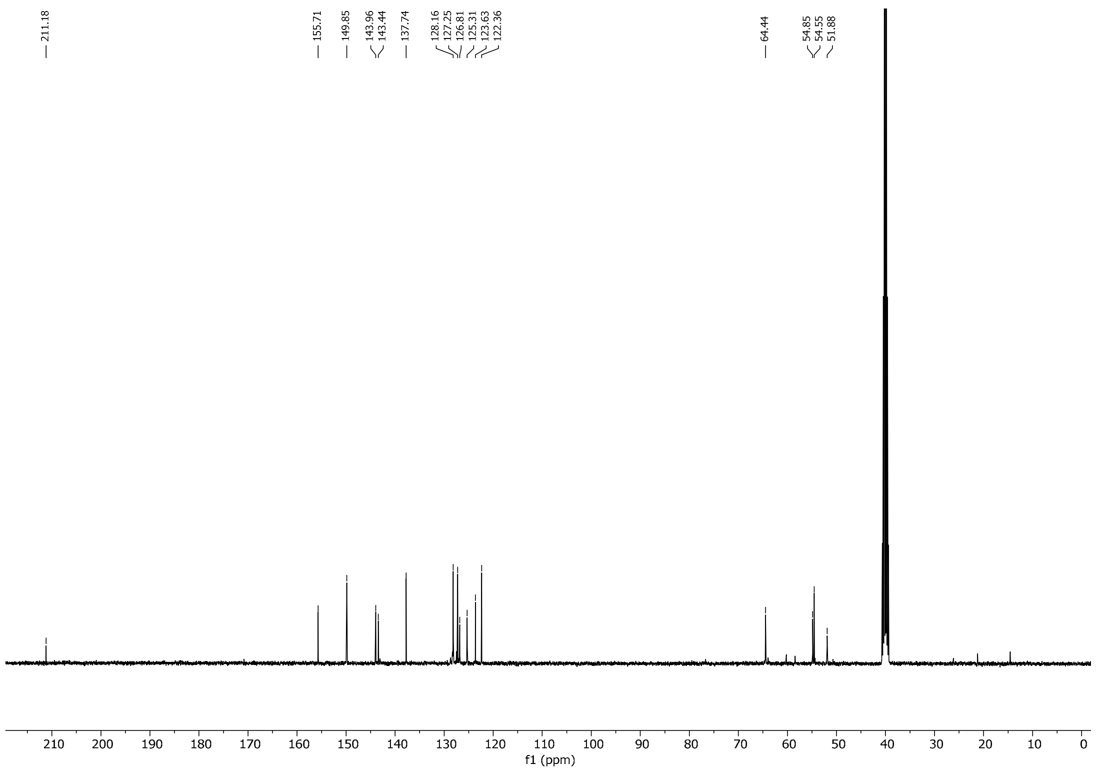


**Figure S19.** $^{1}$H and $^{13}$C-NMR spectra of compound **7d**.

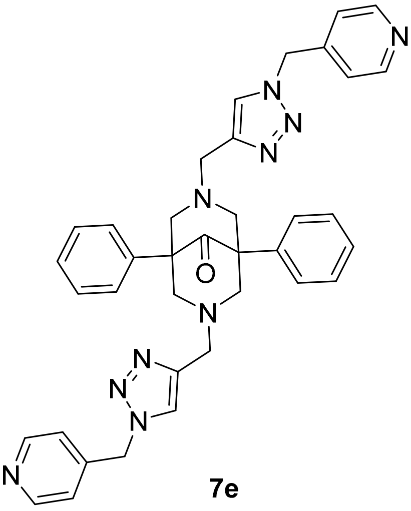

N
N
N
N
N
O
N
N
N
N
N


**7e**

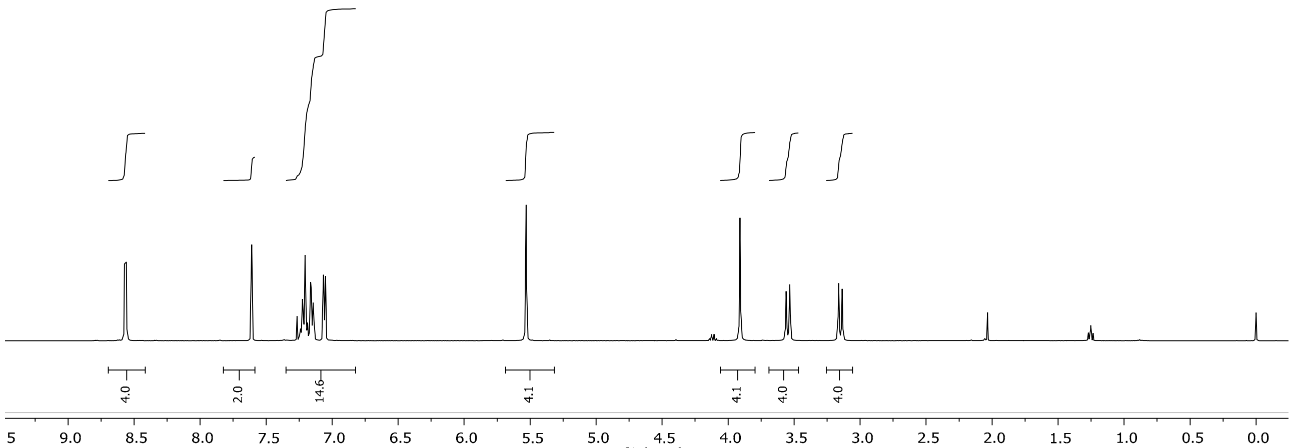

4.0
2.0
14.6
4.1
4.1
4.0
4.0
5
9.0
8.5
8.0
7.5
7.0
6.5
6.0
5.5
5.0
4.5
4.0
3.5
3.0
2.5
2.0
1.5
1.0
0.5
0.0
f1 (ppm)

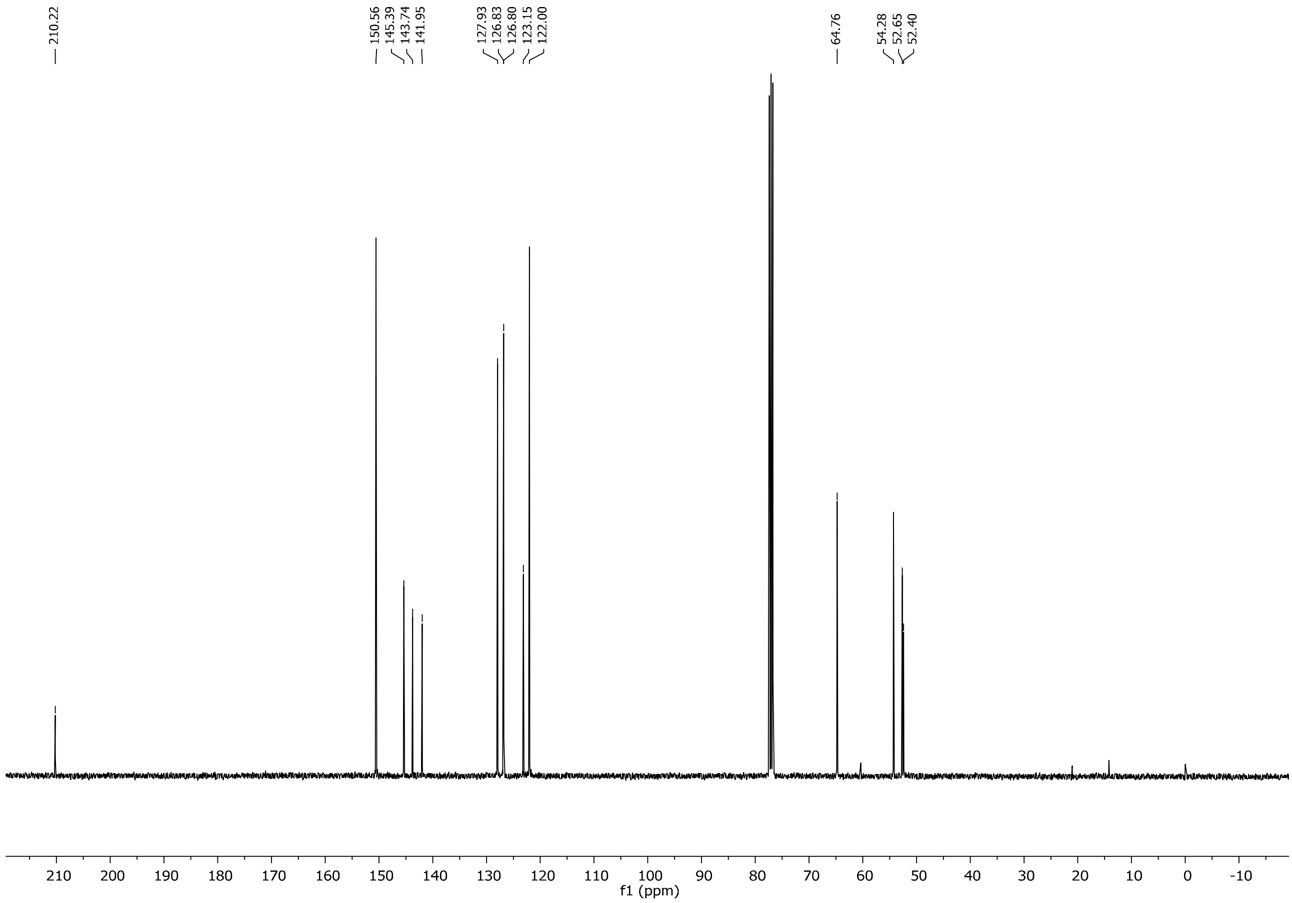

**Figure S20.** $^{1}H$ and $^{13}C$-NMR spectra of compound **7e**.

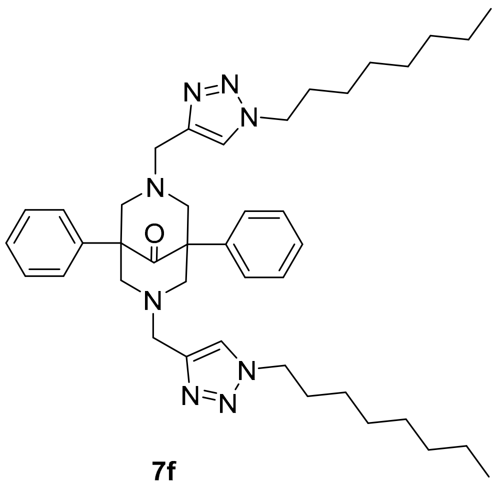

**7f**

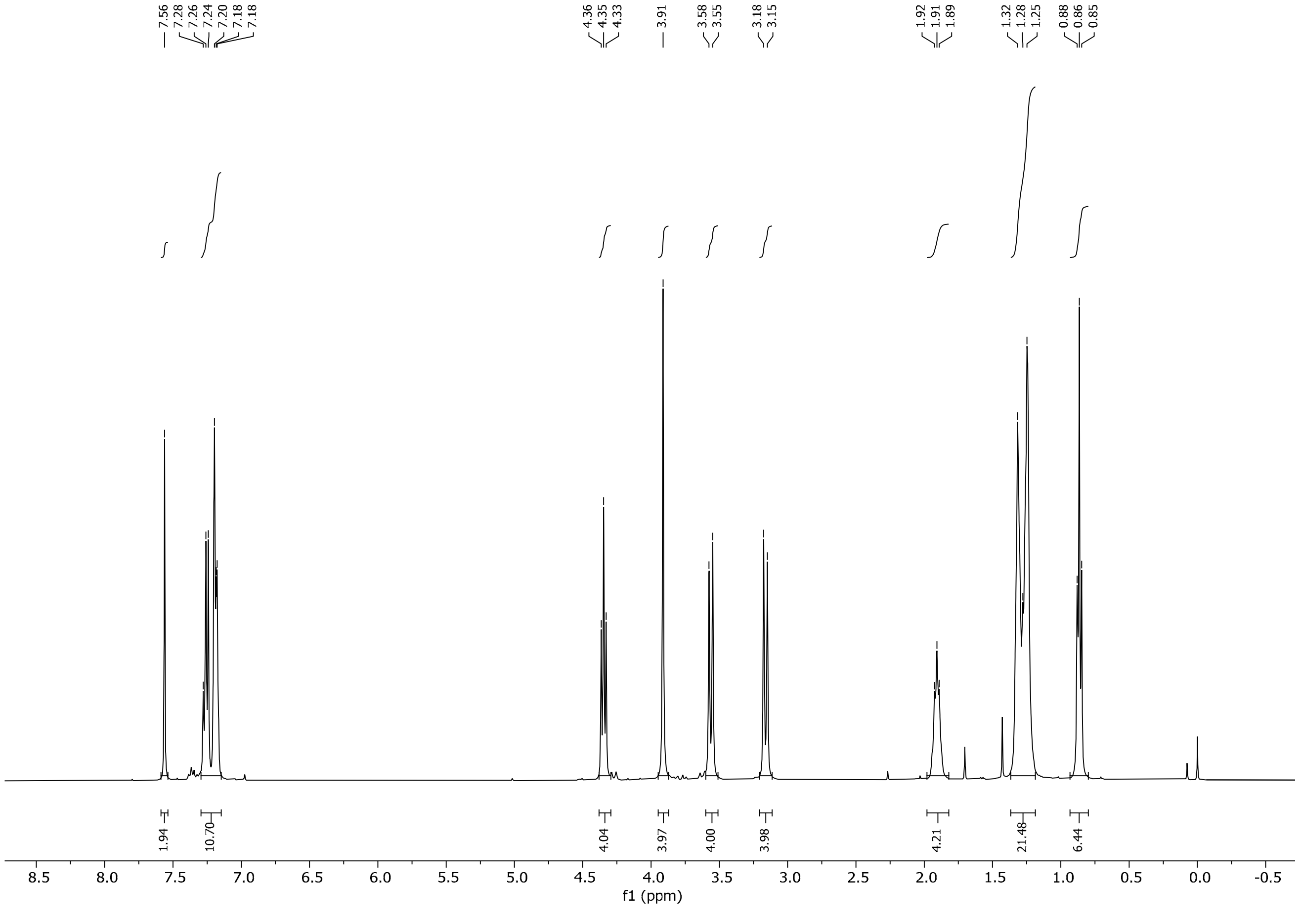
7.56
7.28
7.26
7.24
7.20
7.18
7.18
4.36
4.35
4.33
3.91
3.58
3.55
3.18
3.15
1.92
1.91
1.89
1.32
1.28
1.25
0.88
0.86
0.85
1.94
10.70
4.04
3.97
4.00
3.98
4.21
21.48
6.44
8.5
8.0
7.5
7.0
6.5
6.0
5.5
5.0
4.5
4.0
3.5
3.0
2.5
2.0
1.5
1.0
0.5
0.0
-0.5
f1 (ppm)

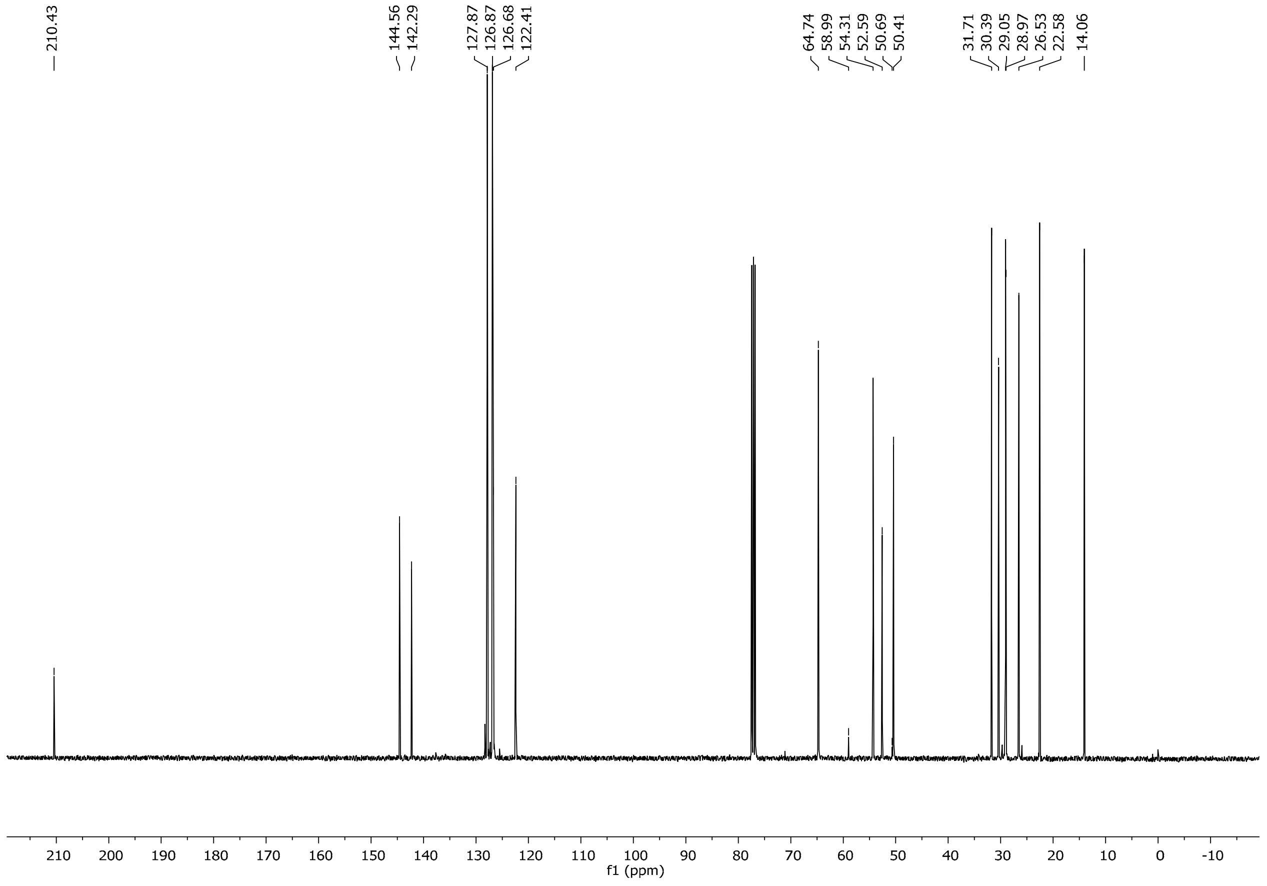


**Figure S21.** $^{1}H$ and $^{13}C$-NMR spectra of compound **7f**.

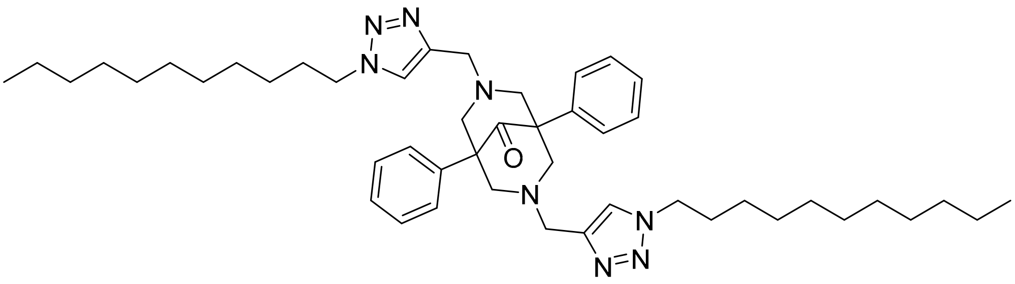
N=N
N
N
N
O
N
N
N=N

**7g**

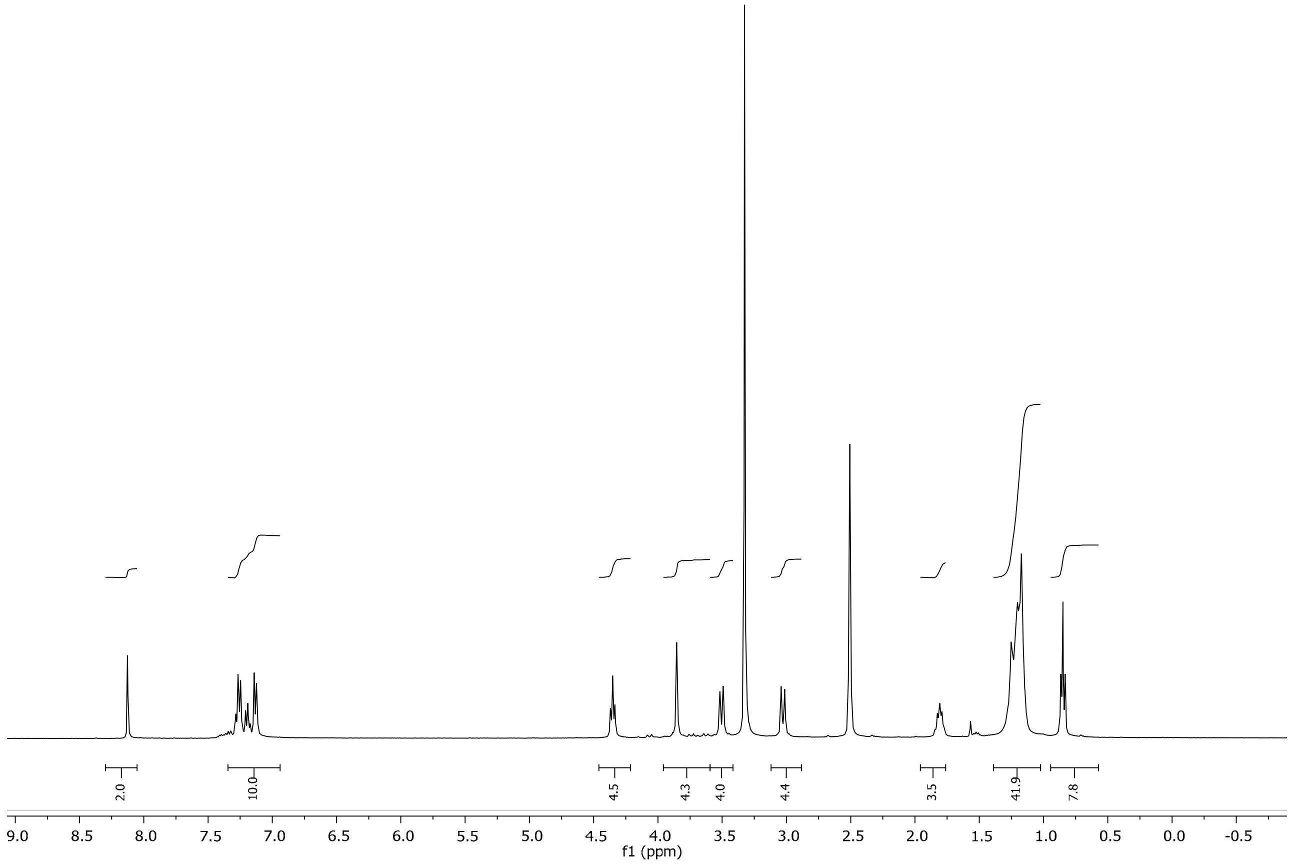
2.0
10.0
4.5
4.3
4.0
4.4
3.5
41.9
7.8
9.0
8.5
8.0
7.5
7.0
6.5
6.0
5.5
5.0
4.5
4.0
3.5
3.0
2.5
2.0
1.5
1.0
0.5
0.0
-0.5
f1 (ppm)

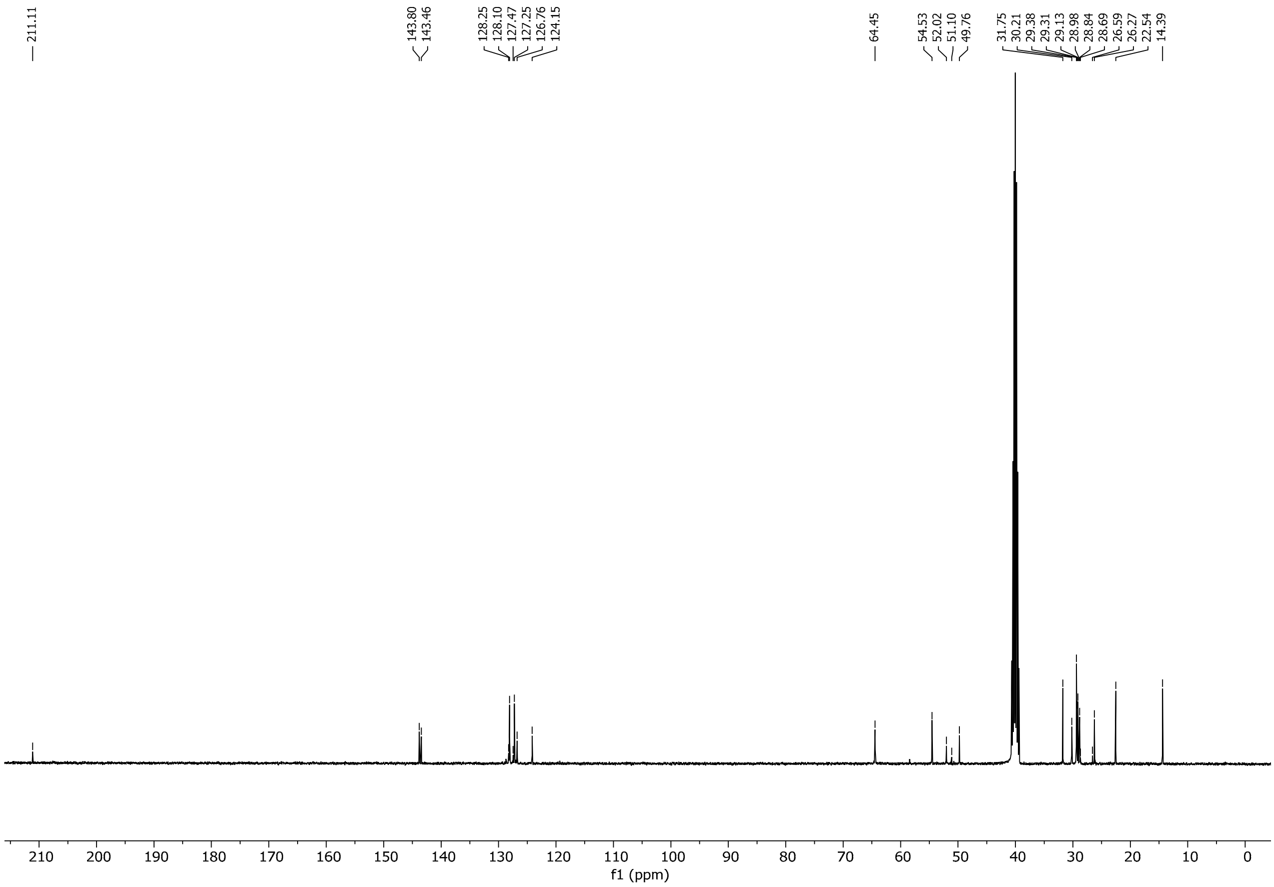


**Figure S22.** $^{1}$H and $^{13}$C-NMR spectra of compound **7g**.

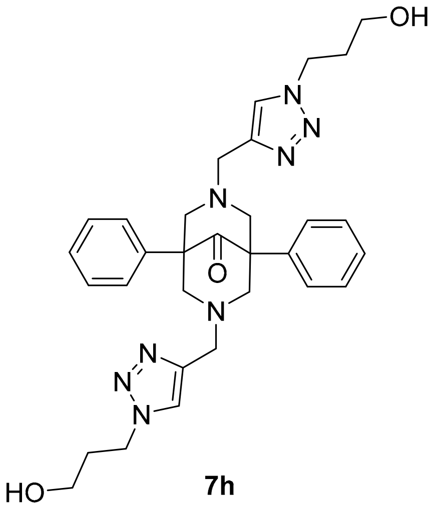
OH
N
N
N
N
O
N
N
N
N
HO

**7h**

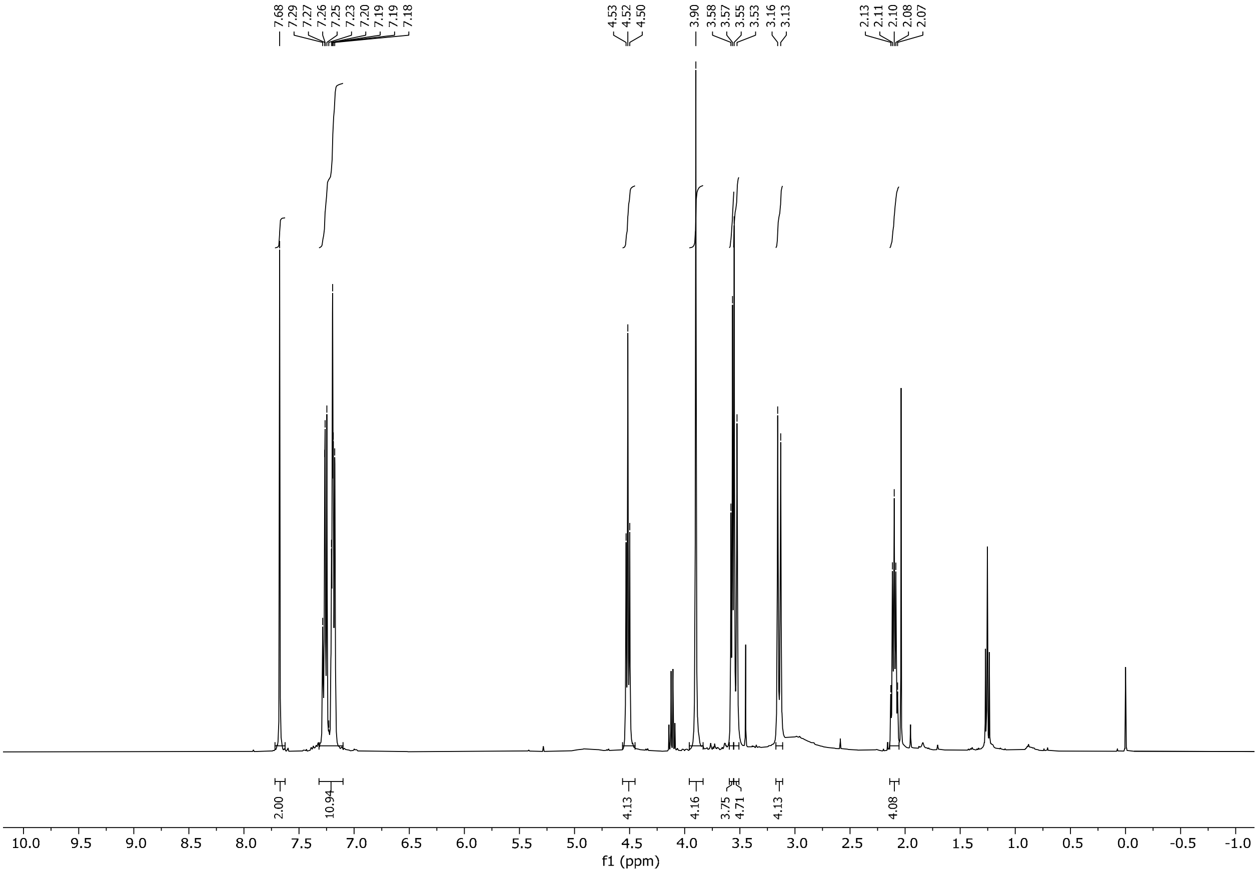
7.68
7.29
7.27
7.26
7.25
7.23
7.20
7.19
7.19
7.18
4.53
4.52
4.50
3.90
3.58
3.57
3.55
3.53
3.16
3.13
2.13
2.11
2.10
2.08
2.07
2.00
10.94
4.13
4.16
3.75
4.71
4.13
4.08
10.0 9.5 9.0 8.5 8.0 7.5 7.0 6.5 6.0 5.5 5.0 4.5 4.0 3.5 3.0 2.5 2.0 1.5 1.0 0.5 0.0 -0.5 -1.0
f1 (ppm)

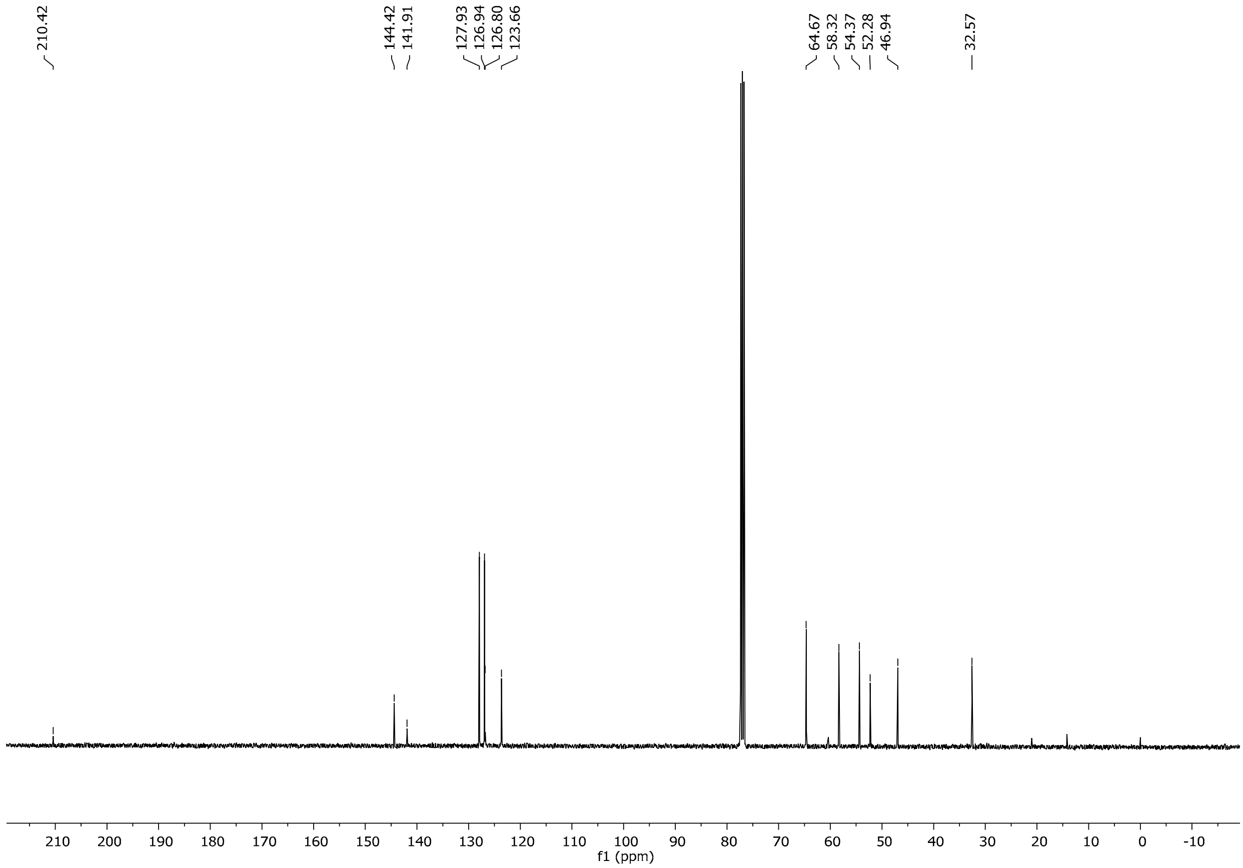


**Figure S23.** $^{1}H$ and $^{13}C$-NMR spectra of compound **7h**.

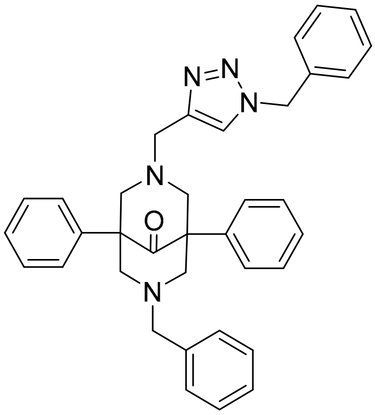

**8a**

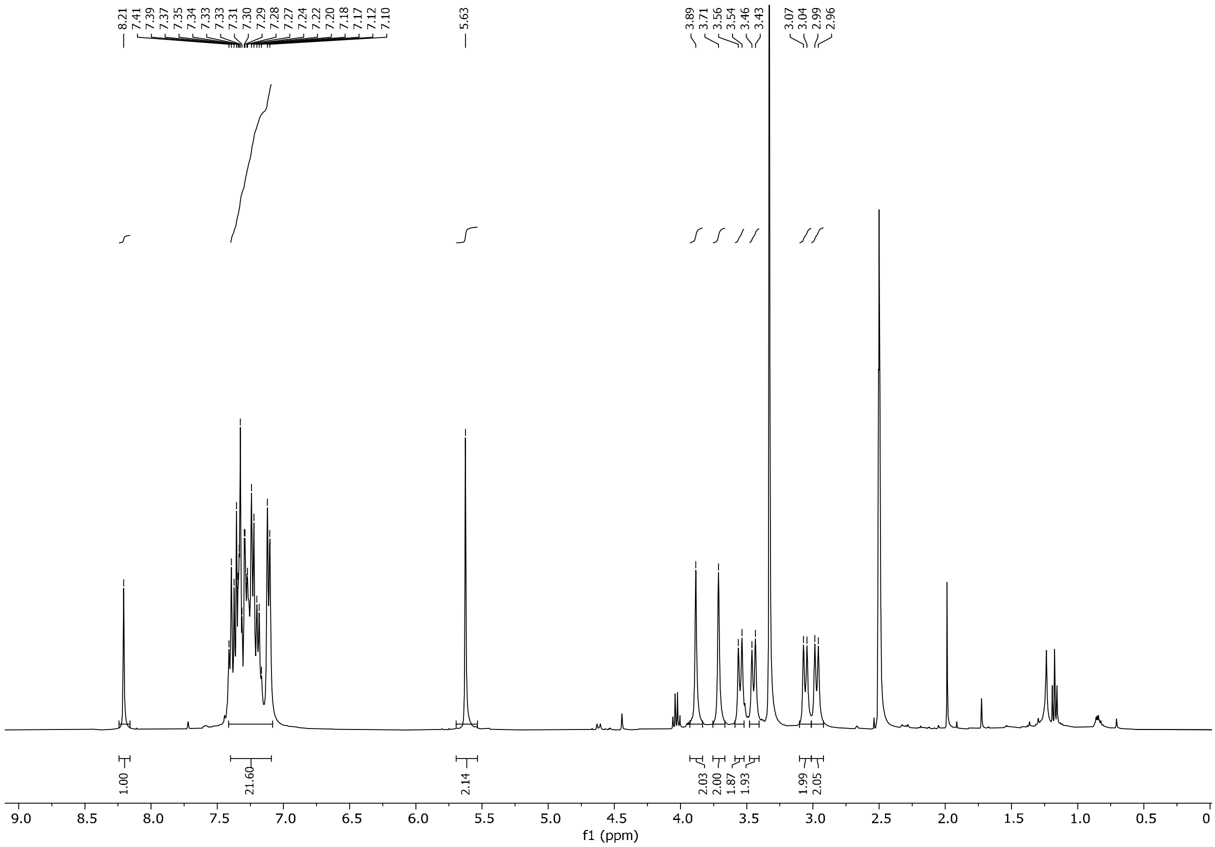
8.21
7.41 7.39 7.37 7.35 7.34 7.33 7.33 7.31 7.30 7.29 7.28 7.27 7.24 7.22 7.20 7.18 7.17 7.12 7.10
5.63
3.89 3.71 3.56 3.54 3.46 3.43
3.07 3.04 2.99 2.96
1.00
21.60
2.14
2.03 2.00 1.87 1.93
1.99 2.05
9.0 8.5 8.0 7.5 7.0 6.5 6.0 5.5 5.0 4.5 4.0 3.5 3.0 2.5 2.0 1.5 1.0 0.5 0
f1 (ppm)

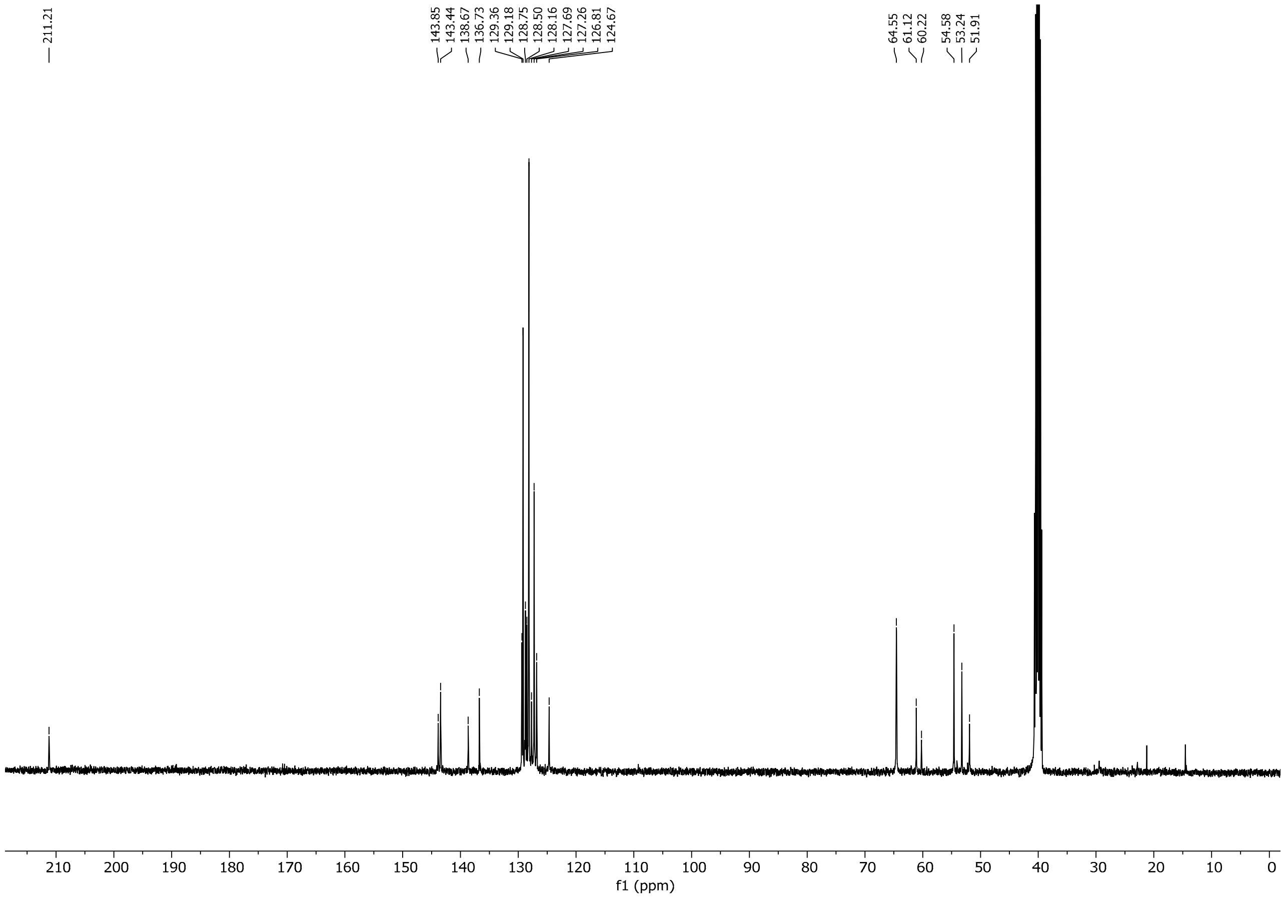


**Figure S24.** $^1$H and $^{13}$C-NMR spectra of compound **8a**.

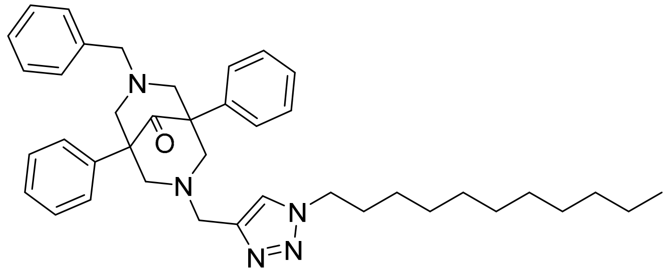

**8b**

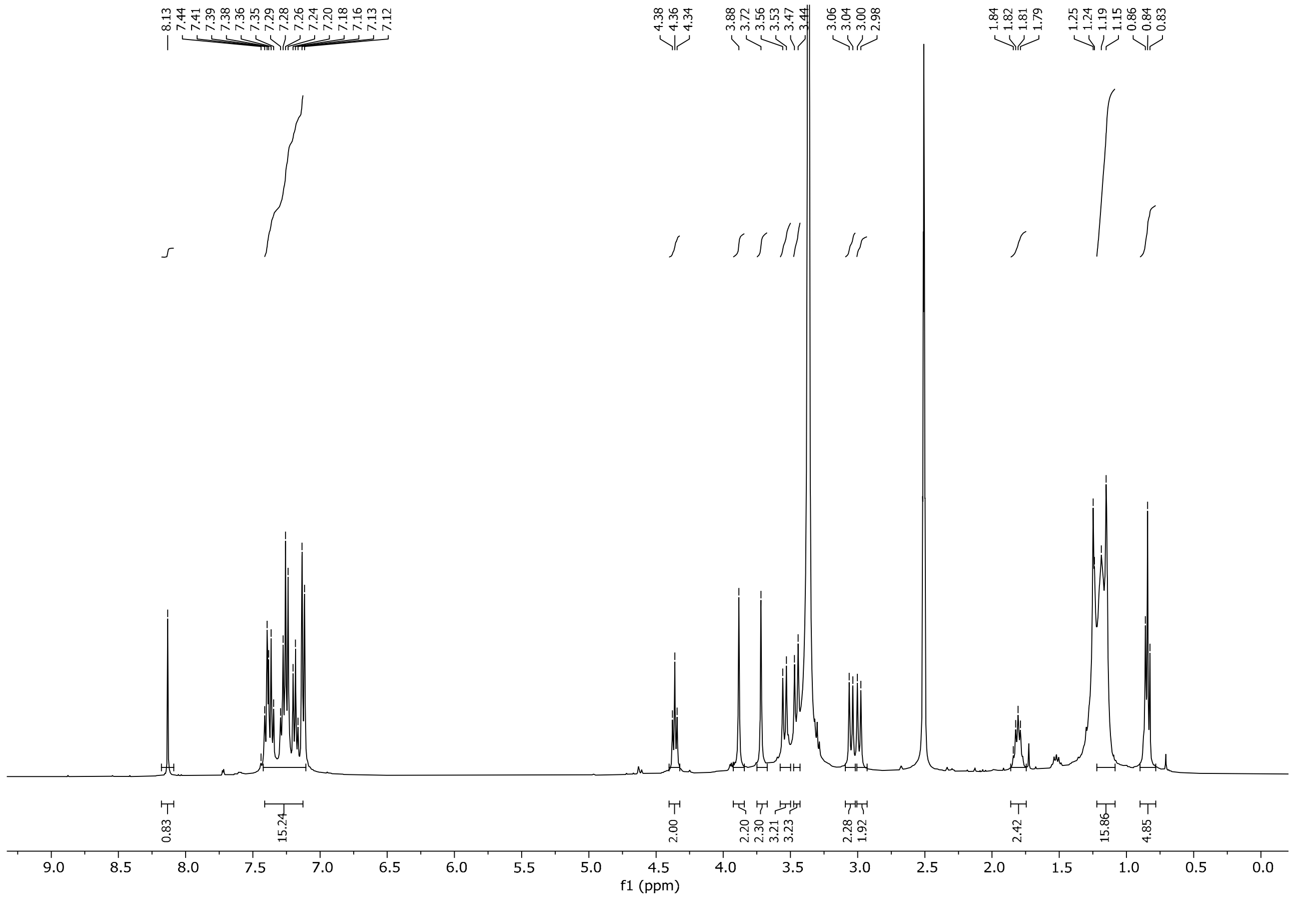

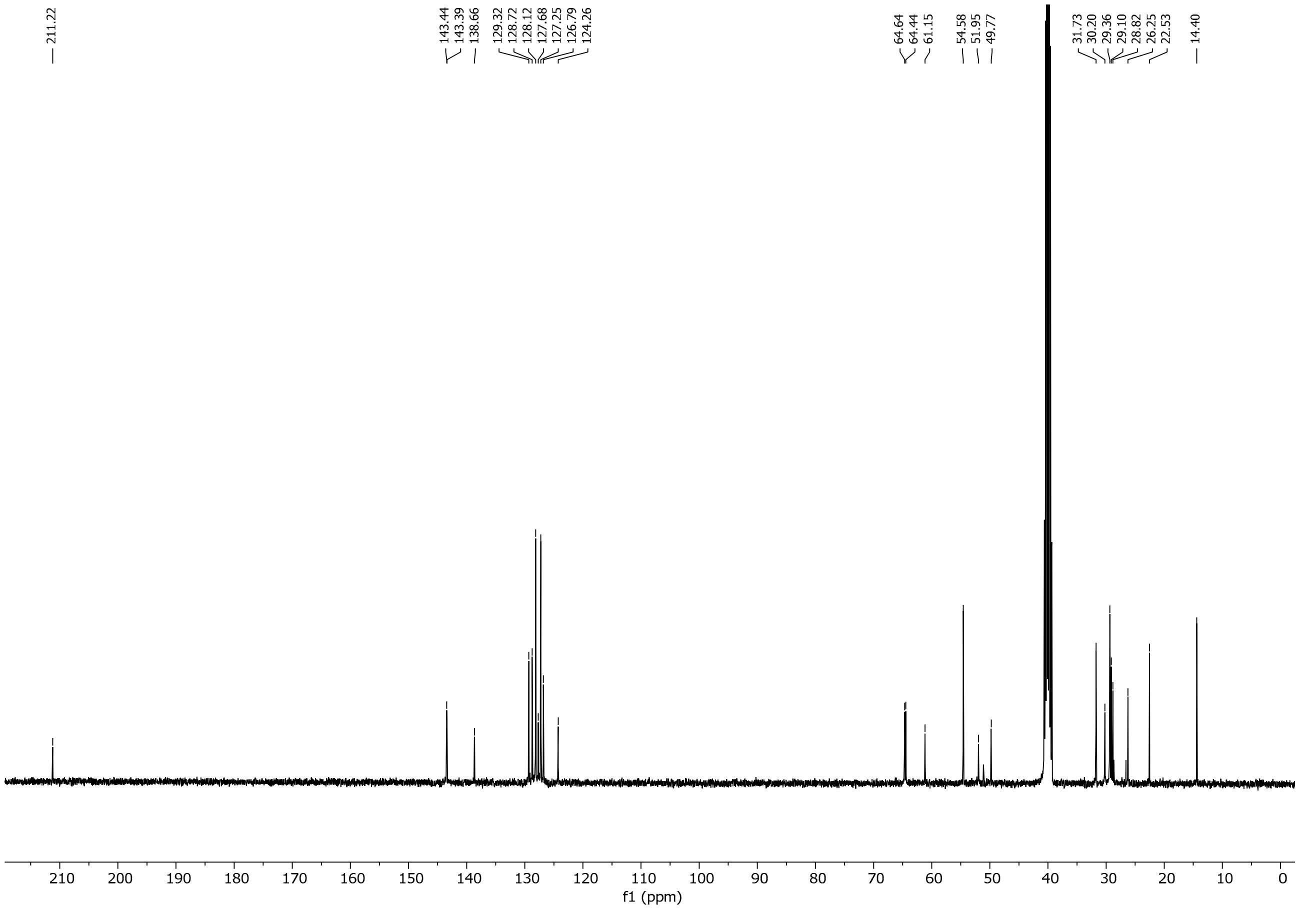


**Figure S25.** $^{1}H$ and $^{13}C$-NMR spectra of compound **8b**.

# 2. NMR TITRATION OF BISPIDINE-METAL COMPLEXES

**Table S1.** Comparison of the free ligands **7a,b,d,h** and their metal complexes: list of the Δδ in the $^{1}$H-NMR spectra.

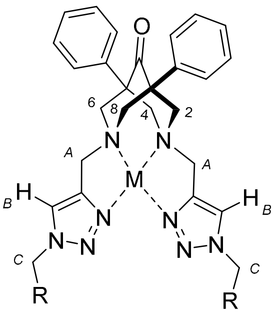


| Entry | Compound | Δδ for $H_{2,4,6,8}$ eq (ppm) | Δδ for $H_{2,4,6,8}$ ax (ppm) | Δδ for $H_A$ (ppm) | Δδ for $H_B$ (ppm) | Δδ for $H_C$ (ppm) |
|---|---|---|---|---|---|---|
| **1** | **7b·Zn(II)** | 0.46 | 0.44 | 0.18 | 0.50 | 0.15 |
| **2** | **7d·Zn(II)** | 0.50 | 0.61 | 0.27 | 0.24 | 0.15 |
| **3** | **7d·La(III)** | 0.45 | 0.70 | 0.43 | 0.23 | 0.03 |
| **4** | **7a·La(III)** | 0.46 | 0.71 | 0.42 | 0.24 | 0.04 |
| **5** | **7h·La(III)** | 0.45 | 0.70 | 0.40 | 0.40 | 0.05 |

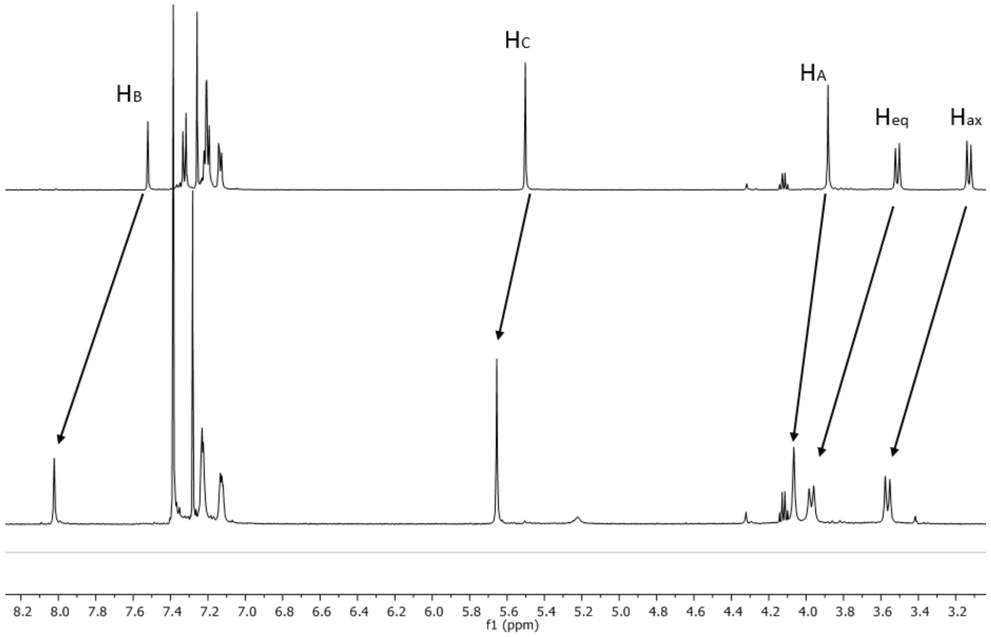


**Figure S26.** $^{1}$H-NMR spectrum of compound **7b** and its Zn(II)-complex, **7b-Zn(II)**.

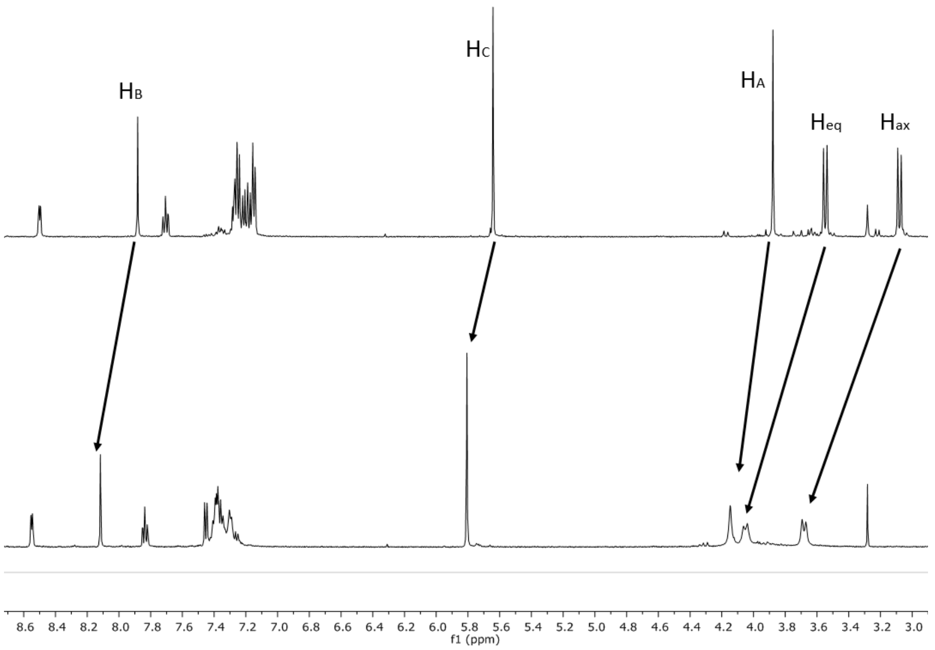


**Figure S27.** $^{1}$H-NMR spectrum of compound **7d** and its Zn(II)-complex **7d-Zn(II)**.

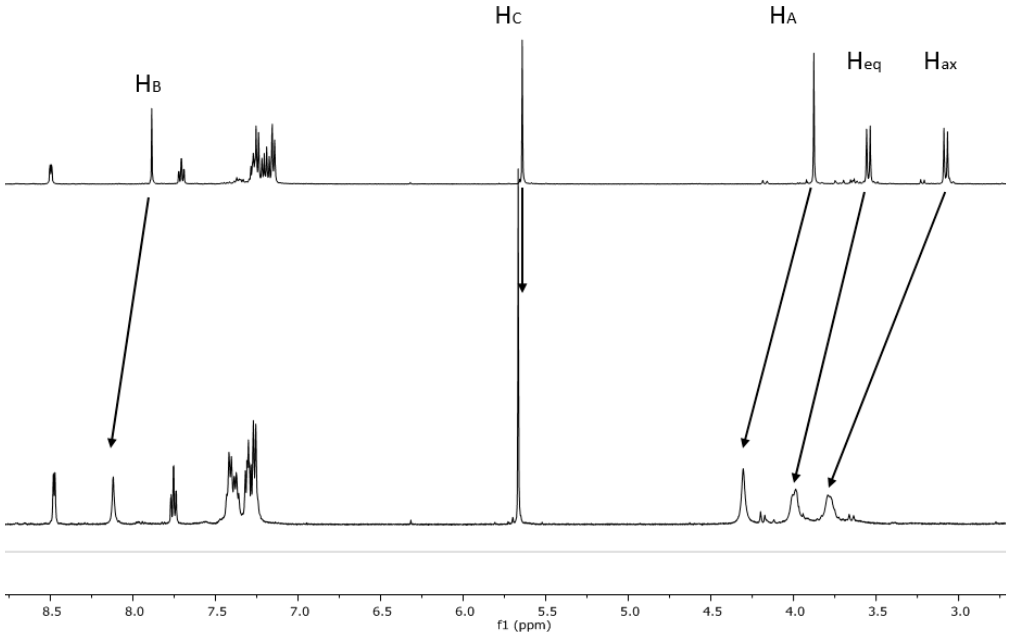


**Figure S28.** $^{1}$H-NMR spectrum of compound **7d** and its La(III)-complex **7d-La(III)**.

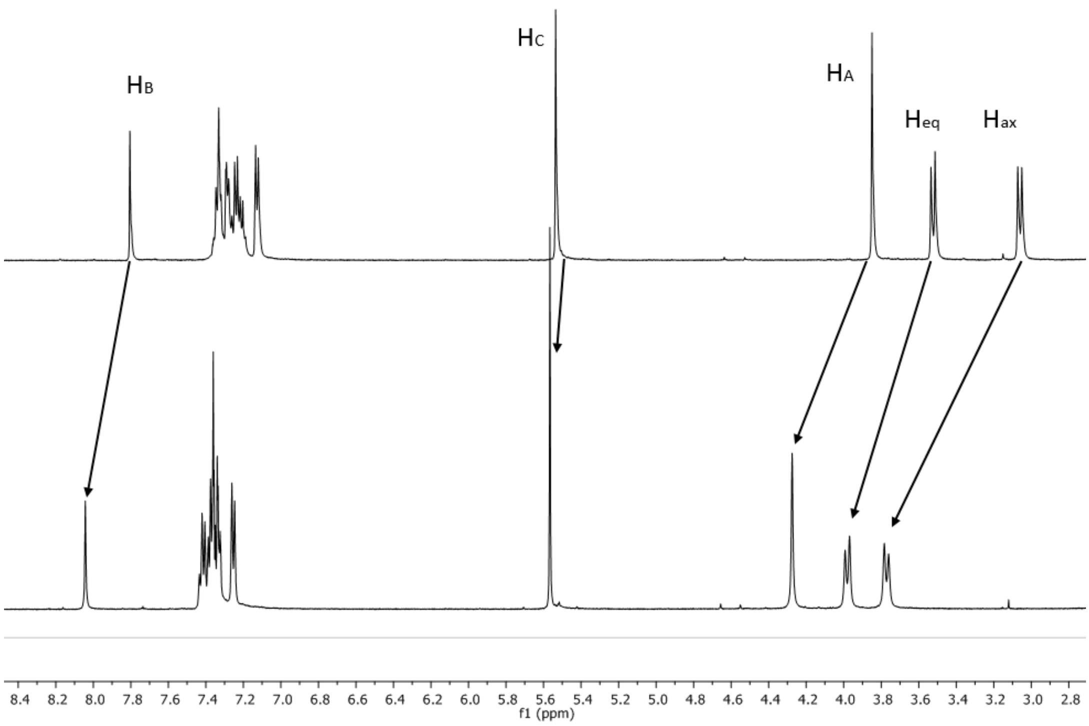


**Figure S29.** $^{1}H$-NMR spectrum of compound **7a** and its La(III) complex **7a·La(III)**.

# 3. ESI-MS ANALYSIS

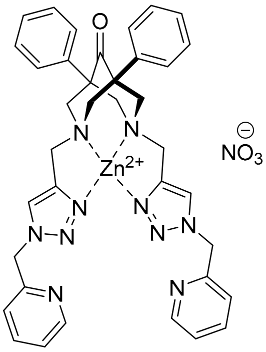


[7d-Zn(II)·$NO_3$]$^+$

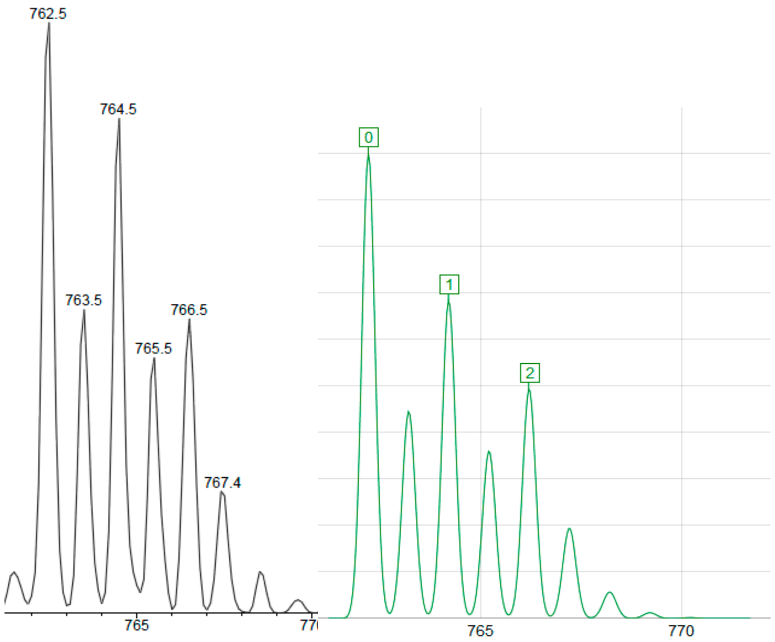


**Figure S30.** Comparison of the experimental ESI-MS spectrum of compound **[7d-Zn(II)·$NO_3$]$^+$** (left) and the simulation of the isotopic pattern for the elemental composition [$C_{37}H_{36}N_{11}O_4Zn$]$^+$ (right).

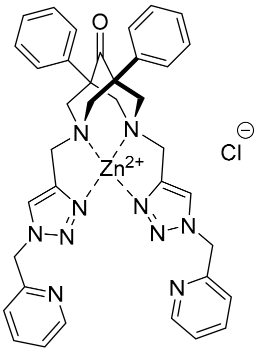


[7d-Zn(II)·Cl]$^+$

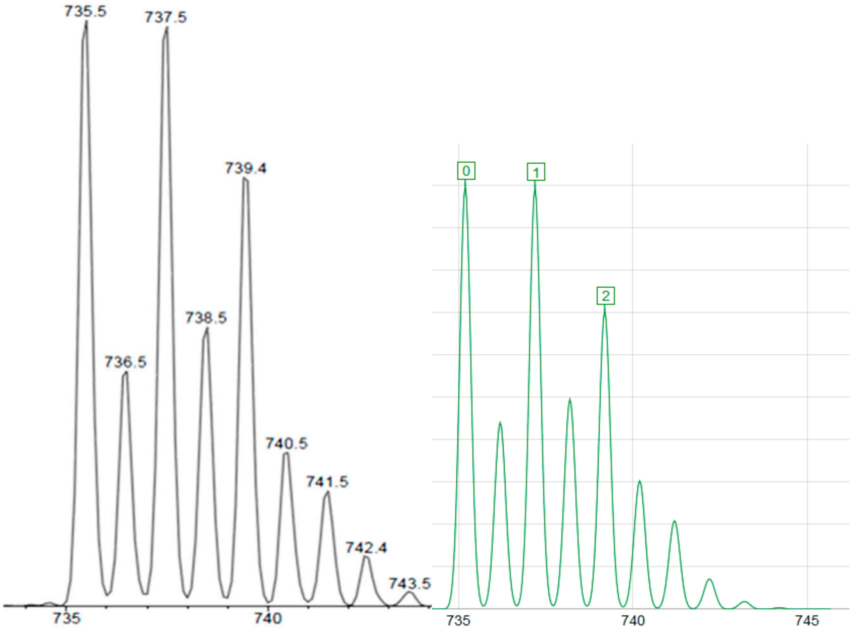


**Figure S31.** Comparison of the experimental ESI-MS spectrum of compound **[7d-Zn(II)·Cl]$^+$** (left) and the simulation of the isotopic pattern for the elemental composition [$C_{37}H_{36}ClN_{10}OZn$]$^+$ (right).

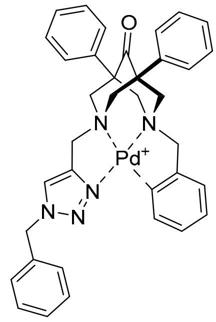


**[8a-Pd(II)]+**

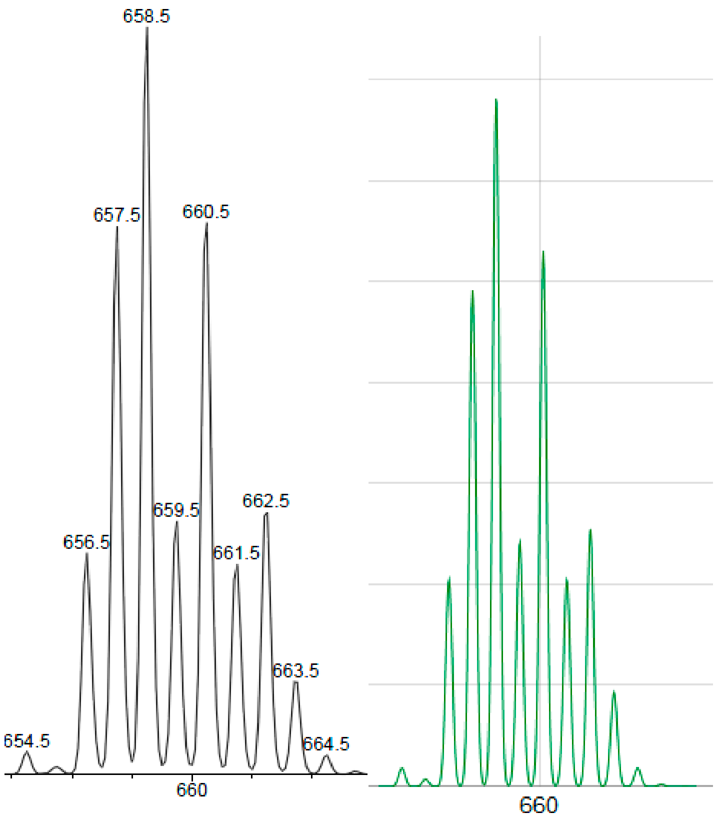


**Figure S32.** Comparison of the experimental ESI-MS spectrum of compound **[8a·Pd(II)]+** (left) and the simulation of the isotopic pattern for the elemental composition [$C_{36}H_{34}N_5OPd$]+ (right).

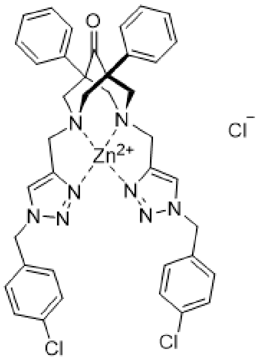


**[7b·Zn(II)·Cl]+**

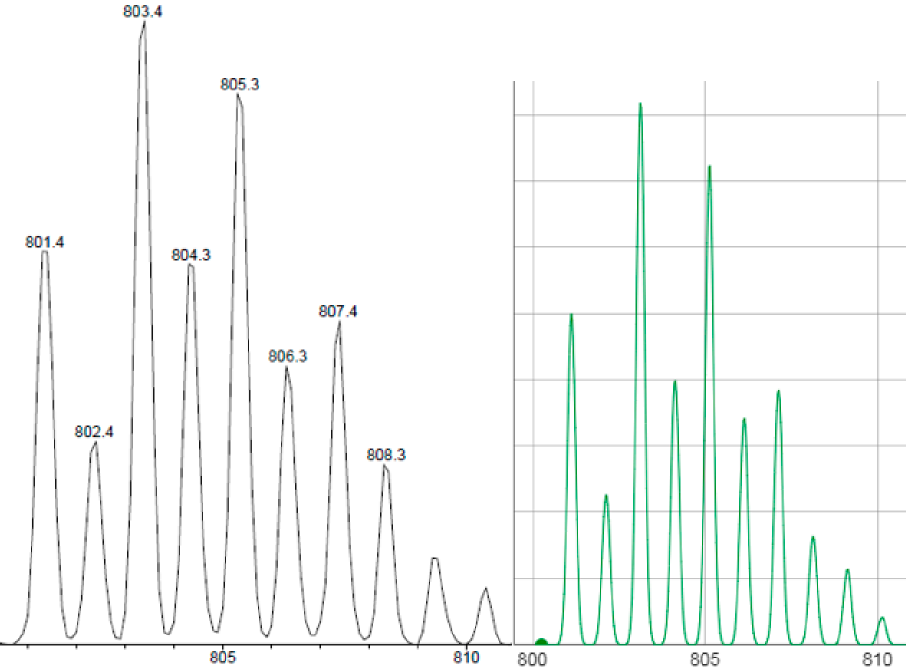


**Figure S33.** Comparison of the experimental ESI-MS spectrum of compound **[7b·Zn(II)·Cl]+** (left) and the simulation of the isotopic pattern for the elemental composition [$C_{39}H_{36}Cl_3N_8OZn$]+ (right).
